# Study on the anti-slide mechanism of double-row circular pile by model test using PIV, transparent soil material and 3D printing technology

**DOI:** 10.1371/journal.pone.0309727

**Published:** 2024-09-25

**Authors:** Lu Cong, Yanchao Wang, Yaohu Hao, Xuanyu Yang, Xuekai Gao, Bichang Zhang

**Affiliations:** 1 Department of Civil Engineering, Shanxi University, Taiyuan, Shanxi Province, PR China; 2 Transportation Industry Key Laboratory of Highway Construction and Maintenance Technology in Loess Area, Shanxi Province Transportation Technology Research and Development CO., LTD., Taiyuan, Shanxi Province, PR China; 3 Shanxi Intelligent Transportation Laboratory, Shanxi Intelligent Transportation Research Institute CO., LTD., Taiyuan, Shanxi Province, PR China; University of Vigo, SPAIN

## Abstract

Landslides are common geological hazards that cause significant losses. Anti-slide piles are commonly used in landslide engineering, and model testing is one of the means to study pile-supported structures. However, model tests face several challenges, including difficulty in controlling the experimental process, challenges in repeated tests, and difficulty in monitoring soil deformation around piles. To address these issues, this study presents a model test method using particle image velocimetry (PIV), transparent soil, and 3D printing technology. Using this method, a series of model tests were conducted, including single-row and double-row anti-slide piles. The experimental results indicate that, compared with single-row piles, double-row piles exhibit better supporting effects. In the pile‒soil interaction, the displacement of the extrusion of soil between piles was controlled under the combined action of the front and back rows of piles. The inclination angle of a single-row pile after the test was 8°, whereas that of a double-row pile was reduced by 62.5%. With respect to the displacement of the soil behind the piles, the phenomenon of a “displacement triangle” behind the piles was observed. An analysis of the change process in this area revealed that the relative displacement caused by pile‒soil interactions is mainly distributed in the surface layer of the soil. The experiments demonstrate that this system is suitable for pile-supported structure model tests.

## 1 Introduction

Landslides are significant geological hazards that threaten infrastructure, human life, and the environment. Among various countermeasures, anti-slide pile structures have gained attention because of their effectiveness in mitigating landslide risk. Model tests serve as invaluable tools for understanding the behavior of pile systems under landslide conditions, aiding in the design of resilient structures [1,2].

Recent studies have focused on enhancing the understanding of pile‒soil interaction mechanisms and optimizing pile design parameters to improve stability against landslides. Comprehensive model tests have been conducted to investigate the load-carrying capacity and deformation characteristics of different pile types under various landslide scenarios. These findings highlighted the importance of the pile diameter and embedment depth in enhancing resistance against lateral soil movements [3,4]. In addition to conventional pile configurations, innovative approaches have been explored to increase landslide resistance. The studies by Iskander et al. [5] and Zhang et al. [6] introduced the concept of utilizing composite materials in pile construction, which demonstrated improved structural performance and reduced material costs. Furthermore, numerical modeling techniques have been integrated with experimental tests to provide insights into the dynamic behavior of pile-supported structures during landslide events [7]. Such interdisciplinary approaches offer valuable predictions for real-world applications. Moreover, advancements in monitoring technologies have enabled real-time assessment of pile performance during model tests. Zhang et al. [8] and Liu et al. [9] integrated fiber optic sensors with pile samples to monitor strain and deformation characteristics under simulated landslide conditions. This approach offers precise measurements, enhances the reliability of experimental results, and facilitates the validation of numerical models.

Physical model testing in geotechnical engineering presents several significant challenges that hinder its effectiveness and efficiency. First, there is a trend toward larger-scale model tests, with dimensions ranging from one meter to several meters [10–12]. However, conducting such large-scale tests is time-consuming, often spans several weeks or even months, and requires substantial resources. Second, maintaining control over the experimental process is difficult, leading to poor repeatability and reproducibility of the results [13,14]. Third, monitoring soil deformation during interactions with structural elements poses significant challenges, particularly in detecting subtle changes [15].

Similar issues are encountered in model tests of anti-slide piles for landslide prevention, exacerbating the inherent challenges of physical model testing. Monitoring deformations around pile foundations is challenging, especially when attempting to detect small-scale soil movements [16]. Additionally, failures in model tests can occur because of the mismatch between the strength of the pile model materials and the required deformations. For example, when attempting to simulate specific landslide forces, the excessive strength of the materials may prevent the desired deformations, leading to test failures and necessitating repetitive testing efforts, which are time-consuming and resource-intensive [17].

Particle image velocimetry (PIV) is a transient, multipoint, nonintrusive laser fluid mechanics measurement method developed in the late 1970s [18]. It has been continuously improved and developed over the past few decades. The characteristics of PIV technology surpass the limitations of single-point velocity measurement techniques. It can record velocity distribution information at numerous spatial points simultaneously, providing rich spatial structure and flow characteristics of the flow field. With the application of PIV technology, the transparent soil model test method provides a visual way to investigate the interaction between soil and structures and deformation in soil or rock masses under different conditions [19,20]. Transparent soil modeling has been successfully applied to study permeability and its mechanism in soil and rock masses with different particle size distributions and shapes [21]. In addition, transparent soil testing has been conducted to examine the response of soil to anti-slide piles [22,23].

3D printing technology and additive manufacturing are commonly used interchangeably to describe computer-guided techniques for creating objects from electronic data sources. Today, 3D printing is considered a revolutionary technology, often referred to as the fourth industrial revolution [24,25]. For the past few years, 3D printing technology has been widely used in architectural design, industrial manufacturing, aerospace, biological engineering, cultural relic protection, and other industries because of its low cost, high efficiency, strong design, and reliable quality [26]. In the field of model tests for geotechnical engineering, 3D printing technology has been successfully applied in rock mechanics analysis and rock engineering [27–30]. Experimental studies of 3D-printed geocells and geogrids have been conducted on footing systems [31,32]. The stability of tunnels was studied by performing model tests with 3D-printed rockbolts [33]. 3D printing technology has been employed in physical experiments conducted to reinforce soil with root systems [34–36]. Model tests with 3D-printed anchors were carried out to research slope stability [37]. However, in the field of model tests for anti-slide piles, the application of 3D printing technology is still relatively rare.

In summary, there is an urgent need for downsizing and intelligent monitoring of model tests, as well as the ability to conduct tests that are repeatable and time and cost-saving. To overcome the current challenges in model tests of anti-slide piles for landslide prevention, including difficulty in controlling the experimental process, challenges in repeating tests, and difficulty in monitoring soil deformation around piles, a new model test system was developed using PIV, transparent soil, and 3D printing technology. The model test method in this paper can be used for analyzing the deformation evolution of anti-slide piles, studying the interaction between piles and soil, and demonstrating geotechnical experiments for teaching purposes.

## 2 The problem in the design process of anti-slide piles

In the process of landslide engineering design, a single row of large-diameter anti-slide piles is insufficient to support the sliding force [38]. Furthermore, the shear and bending moments of these anti-slide piles often meet the design requirements, yet there is often a significant displacement of the top of the pile. This ultimately leads to the failure of the calculations. At this point, it is assumed that the design of a row of small-diameter anti-slide piles in front of the large-diameter anti-slide piles can either reduce the displacement of the top of the pile or enhance the effectiveness in supporting the landslide. This is the basic hypothesis of this paper.

Moreover, many scholars have proposed the use of h-type anti-slip piles, with the same shape and size as the front and back rows of anti-slide piles, to support landslides [39–43]. However, the anti-slip pile structure proposed in this paper differs from those previously studied in that it has not been adequately studied. The evolution of soil displacement behind piles remains poorly defined. This is also the original intention of this paper.

## 3 Model test system

### 3.1 Structure of the model test system

**Fig 1:**
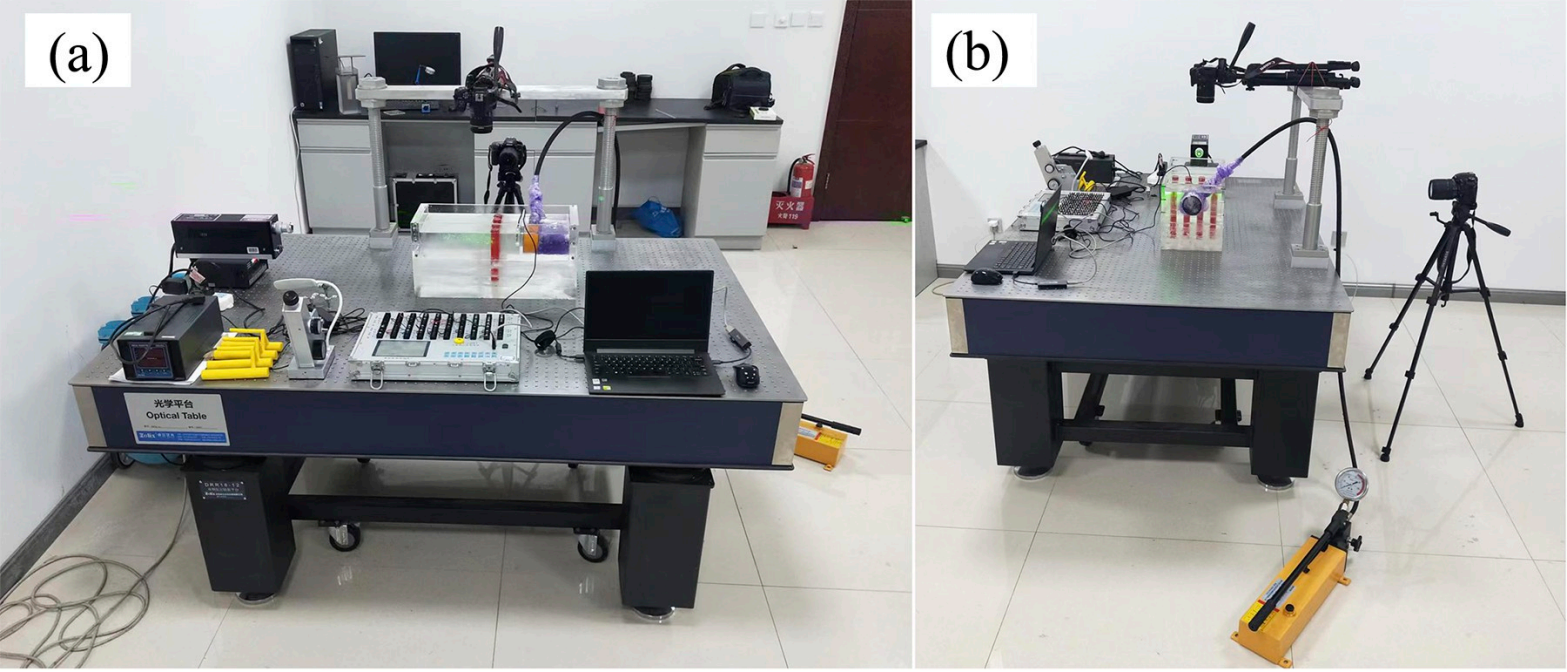
The anti-slide pile model test system: (a) front view; (b) side view.

The anti-slide pile model test system developed in this study (Fig 1) includes the following components: (1) a model box (transparent soil, 3D printed anti-slide piles); (2) a loading system (hydraulic jack); (3) a monitoring system (camera, PIV system, computer); and (4) a vibration isolation platform.

### 3.2 Transparent soil

#### 3.2.1 Materials

The solid granular material used in this study is fused quartz sand, which resembles white sugar in appearance and has irregularly angular grains; its main components include SiO_**2**_, Al_**2**_O_**3**_, CaO, MgO, Na_**2**_O, etc., with SiO_**2**_ contents greater than 99.9%. Fused quartz sand exhibits stable physical and chemical properties, with extremely low thermal conductivity and excellent thermal stability. This sand has uniform particles, high purity, minimal impurities, colorless transparency, and good optical properties, with a refractive index of 1.4585. Unlike silica gel, it lacks fine pores internally, making it more suitable for simulating natural sand. Fused quartz sand has a Mohs hardness of approximately 7.0, a pH value of approximately 6.0, and a specific gravity of 2.2, which are similar to the properties of natural sand. Numerous experimental results have shown that, compared with those of other transparent soil materials, the properties of fused quartz sand are closer to those of natural sand, and the transparency of soil prepared from it is relatively good, making it widely applicable in geotechnical engineering model tests. Many scholars have conducted a series of indoor model tests using transparent soil prepared from quartz sand and have reached several useful conclusions [21–23].

In this work, a mixture of #15 white oil and n-dodecane (normal dodecane) was used as the pore mixture. The #15 white oil is a colorless, odorless, transparent oily liquid with good chemical stability and photostability. N-dodecane is also colorless, odorless, transparent, commonly used as an intermediate in organic synthesis, and soluble in ethanol, diethyl ether, etc.

#### 3.2.2 Preparation protocol for transparent soil

Several types of solid granular materials and pore fluids with the same refractive index can be used to prepare corresponding transparent soils. Taking fused quartz sand with a particle size of approximately 0.5–1 mm and mixed oil as examples of solid granular material and pore fluid, respectively, the preparation process mainly includes six steps: selecting solid materials, selecting solution materials, measuring the refractive index, mixing uniformly, vacuuming, and solidifying. The shear strength parameters of the transparent soil from the undrained and unconsolidated straight shear tests were an internal friction angle of approximately 33° and a cohesion angle of approximately 0 kPa, similar to the data from Kong et al. [44].

The specific operation methods are as follows:

1. The quartz sand was washed and dried to remove moisture and impurities.
2. Dodecane and #15 white oil were mixed at a mass ratio of approximately 1:4 and stirred to mix evenly.
3. The refractive index of the mixed liquid was measured with an Abbe refractometer, and the mass of both was adjusted according to the temperature to achieve a refractive index of 1.4585.
4. Glass beads were poured slowly and evenly into the mixed liquid and stirred to ensure thorough mixing. The liquid level was slightly greater than that of the glass beads.
5. The mixture was placed in a vacuum chamber and allowed to stand to remove residual air.

**Fig 2:**
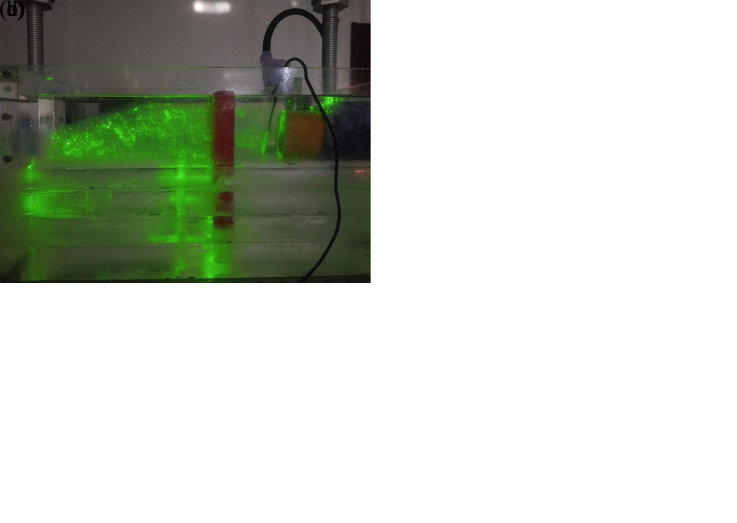
Transparency and speckle effects of the transparent soil in the model test: (a) transparency effect (top view); (b) speckle effect (top view); (c) transparency effect (side view); (d) speckle effect (side view).

Speckle images are produced by reflected and scattered light beams from rough surfaces through free space to observe planes when the laser illuminates the object surface. The prepared transparent soil interacts with the laser to produce a speckle field, which is suitable for subsequent image processing. The transparency and speckle effects of the transparent soil produced in this study are shown in Fig 2, which shows that, whether viewing from the top or side, the transparent soil is seen to exhibit good transparency and speckle effects.

#### 3.3.3 3D-Printed anti-slide pile

The anti-slide piles are produced via a 3D printer. The advantage of 3D printing lies in its ability to create anti-slide pile models of various shapes with high dimensional accuracy. This method enables rapid and repetitive printing, significantly enhancing production efficiency. 3D printers can produce models with different infill densities and patterns, which are factors controlling the strength of the anti-slide pile models. Moreover, the choice of 3D printing materials can also influence the strength of the models.

In this work, the model test was carried out via two types of 3D-printed anti-slide piles. The first type was a large-diameter single-row anti-slide pile, the diameter of the single-row anti-slide piles was 25 mm, and the length was 160 mm. To compare the supporting effect of the two-row anti-slide piles, the second type was designed as double-row anti-slide piles, consisting of front-row and back-row anti-slip piles, and the size of the back-row anti-slip piles was the same as that of the first type of anti-slide piles. The front-row anti-slip piles were small piles, with a diameter of 10 mm and a length of 100 mm, connected to the back row of piles by a 10 mm diameter round connecting beam. The pile center distance between the front and back rows is 50 mm.

**Fig 3:**
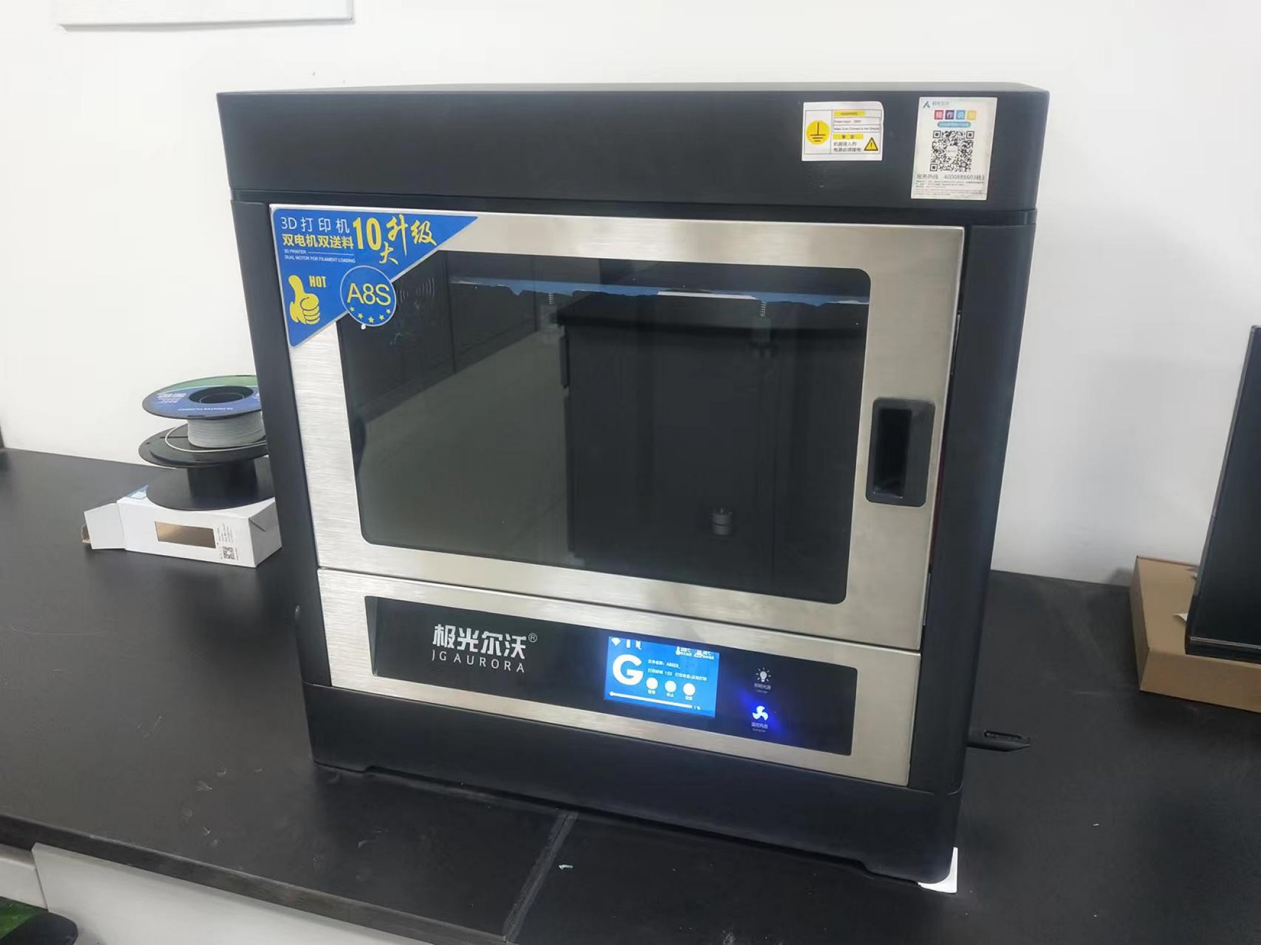
JG MAKER Aurora A8S 3D printer.

**Fig 4:**
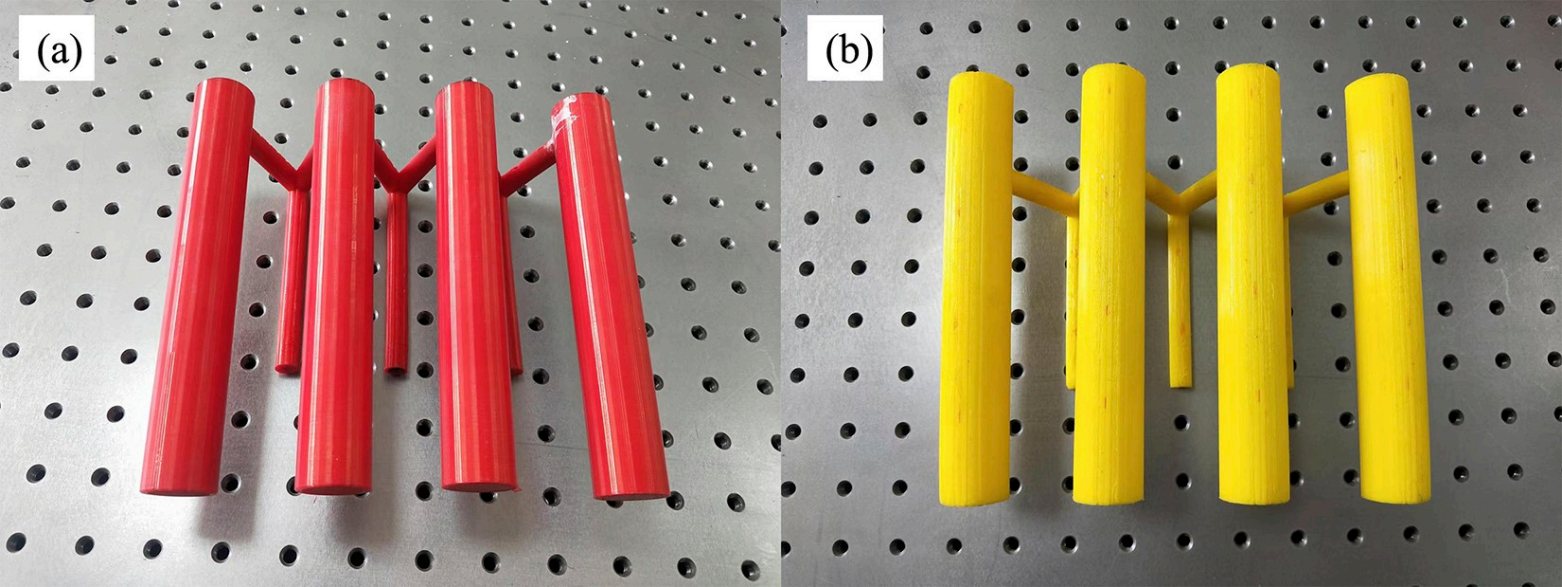
Double-row anti-slide pile model printed by 3D printer: (a) red; (b) yellow.

In this study, the 3D printer model used is the JG MAKER Aurora A8S (Fig 3), and the printing material is TPU K7 95A-type 3D printing material. The 3D printing process involves creating the model in SolidWorks software, slicing it using JGcreat software, and then selecting different infill patterns for 3D printing. Common infill patterns for 3D printing include triangular, square, spiral, and cubic patterns, and the strength of the printed pile varies depending on the chosen infill pattern. The printed anti-slide piles are shown in Fig 4. With a printing precision of 0.5 mm, the printing time for a single pile is approximately 3 hours.

### 3.4 Model test box

**Fig 5:**
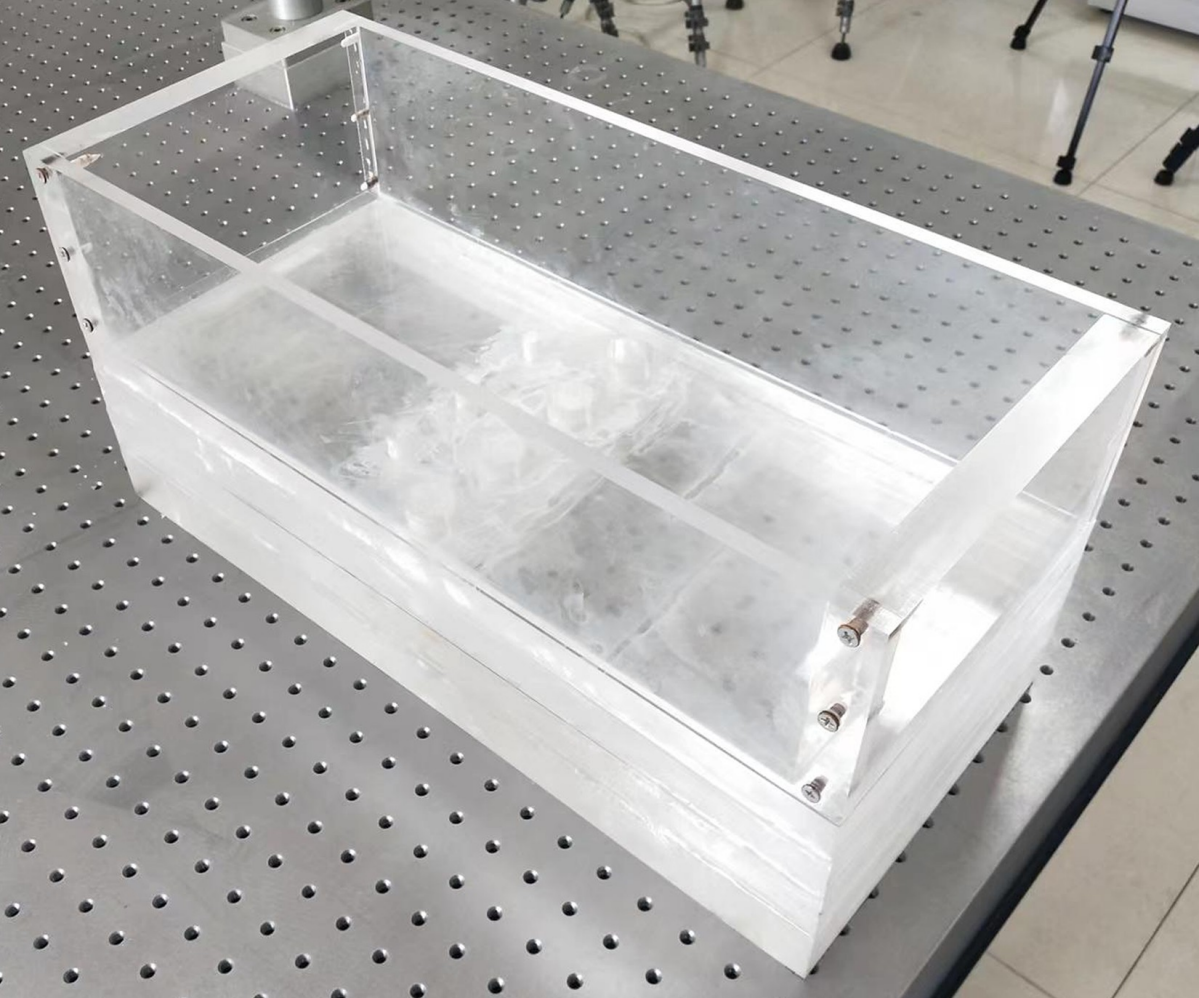
The model box.

The model test box is constructed by cutting and assembling multiple layers of transparent acrylic sheets. The purpose of using transparent acrylic sheets is to allow the camera to capture images of the deformation of the transparent soil material. The exterior dimensions of the model box are 220 mm × 500 mm × 235 mm, whereas the test space dimensions inside the model box are 200 mm × 450 mm × 110 mm. The model test box is shown in Fig 5. A piece of acrylic board was placed as a loading plate inside the model box.

In this work, the slip surface of the model box is perpendicular to the anti-slide pile, and according to Xie et al. [23], improving the bottom inclination of the model box makes it possible to simulate conditions with different inclination angles of the slip surface.

### 3.5 Loading system

**Fig 6:**
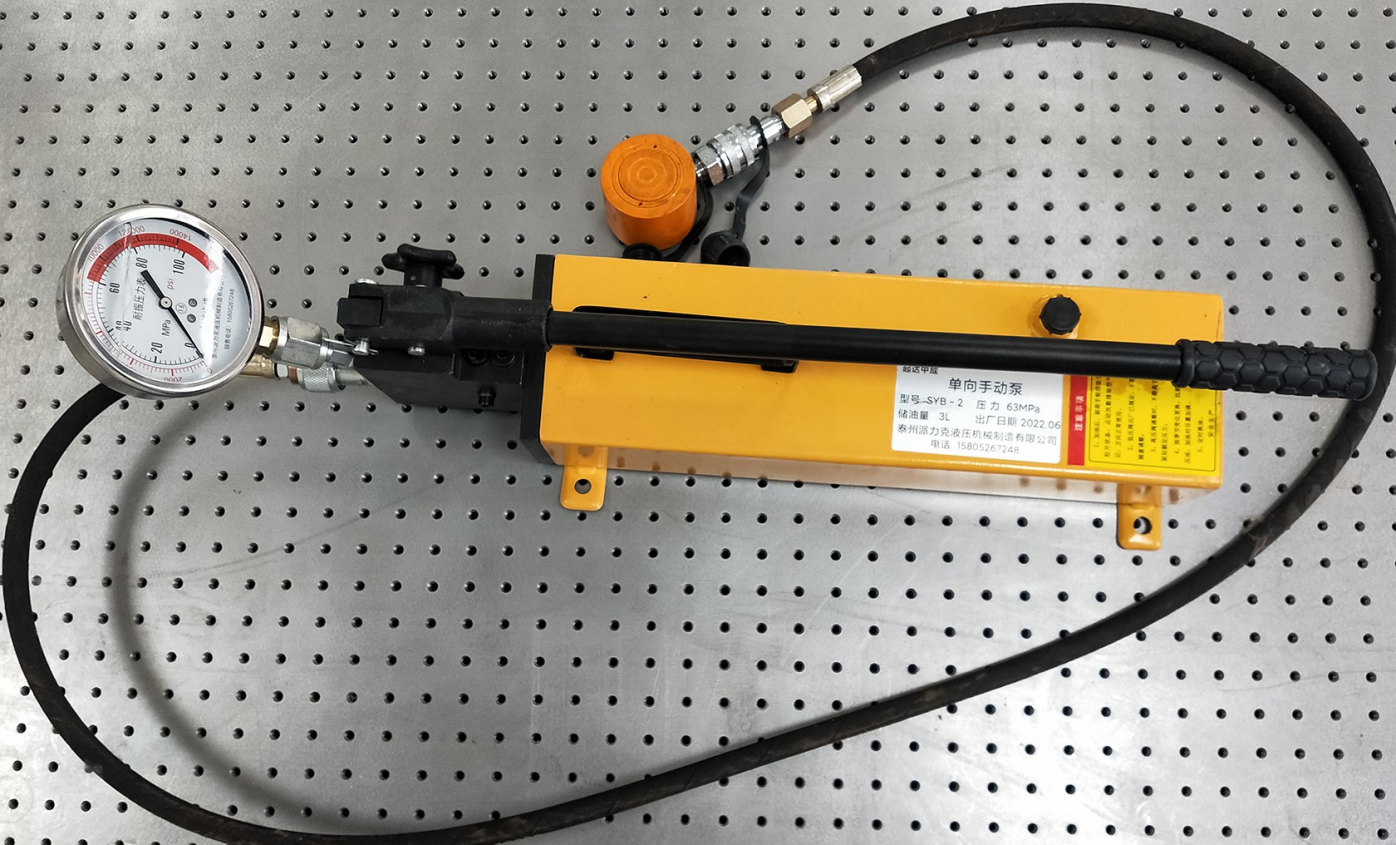
Loading system.

**Fig 7:**
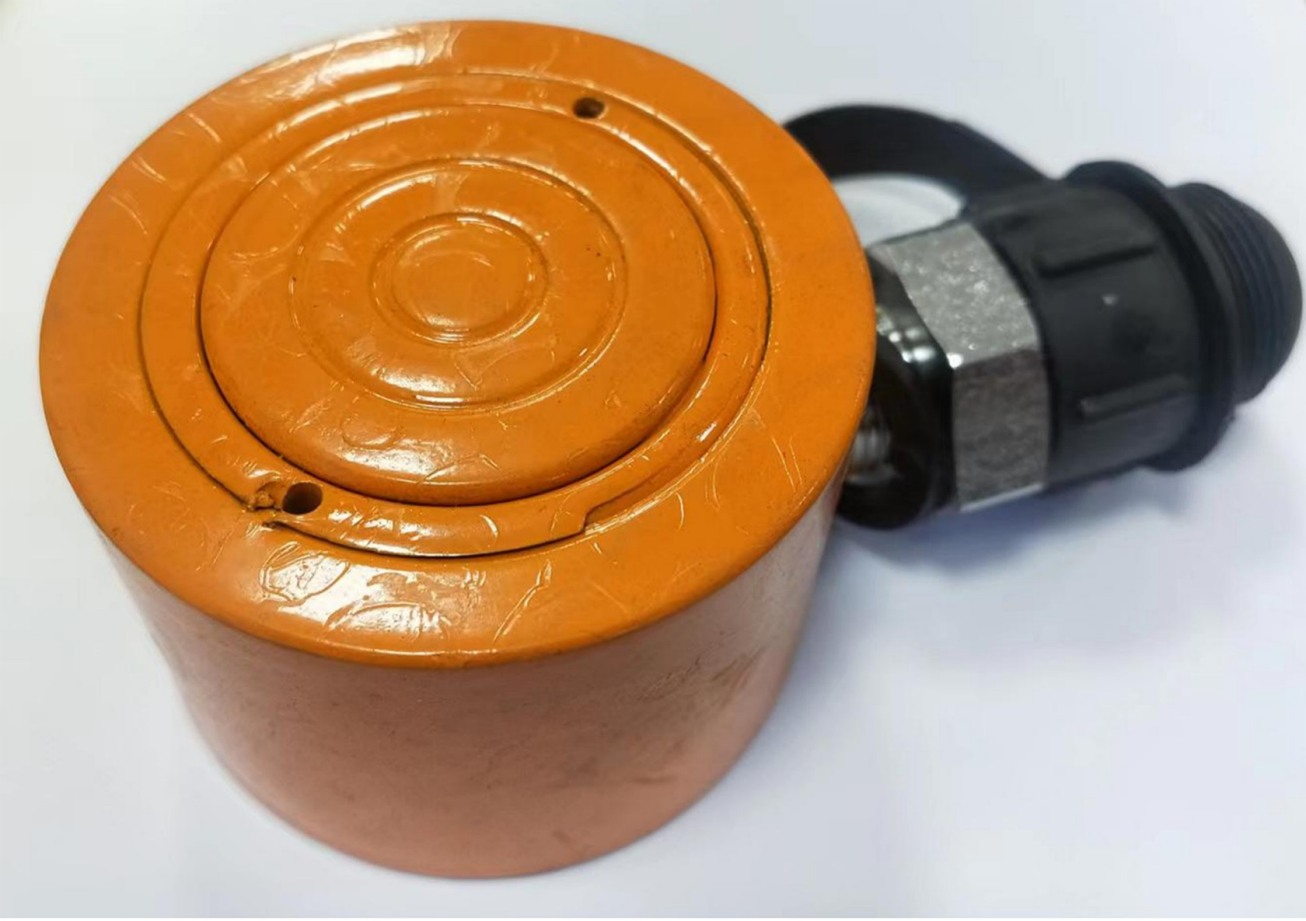
Ultra-thin hydraulic jack.

The loading system uses hydraulic principles to simulate the landslide thrust resisted by anti-slide piles. It consists of an ultrathin hydraulic jack, a loading plate, and a hydraulic pump (Fig 6). The piston of the ultrathin hydraulic jack is cylindrical (Fig 7), with a height of 45 mm and a diameter of 65 mm. It can be placed inside the model box to apply a load to the model material.

### 3.6 PIV system

**Fig 8:**
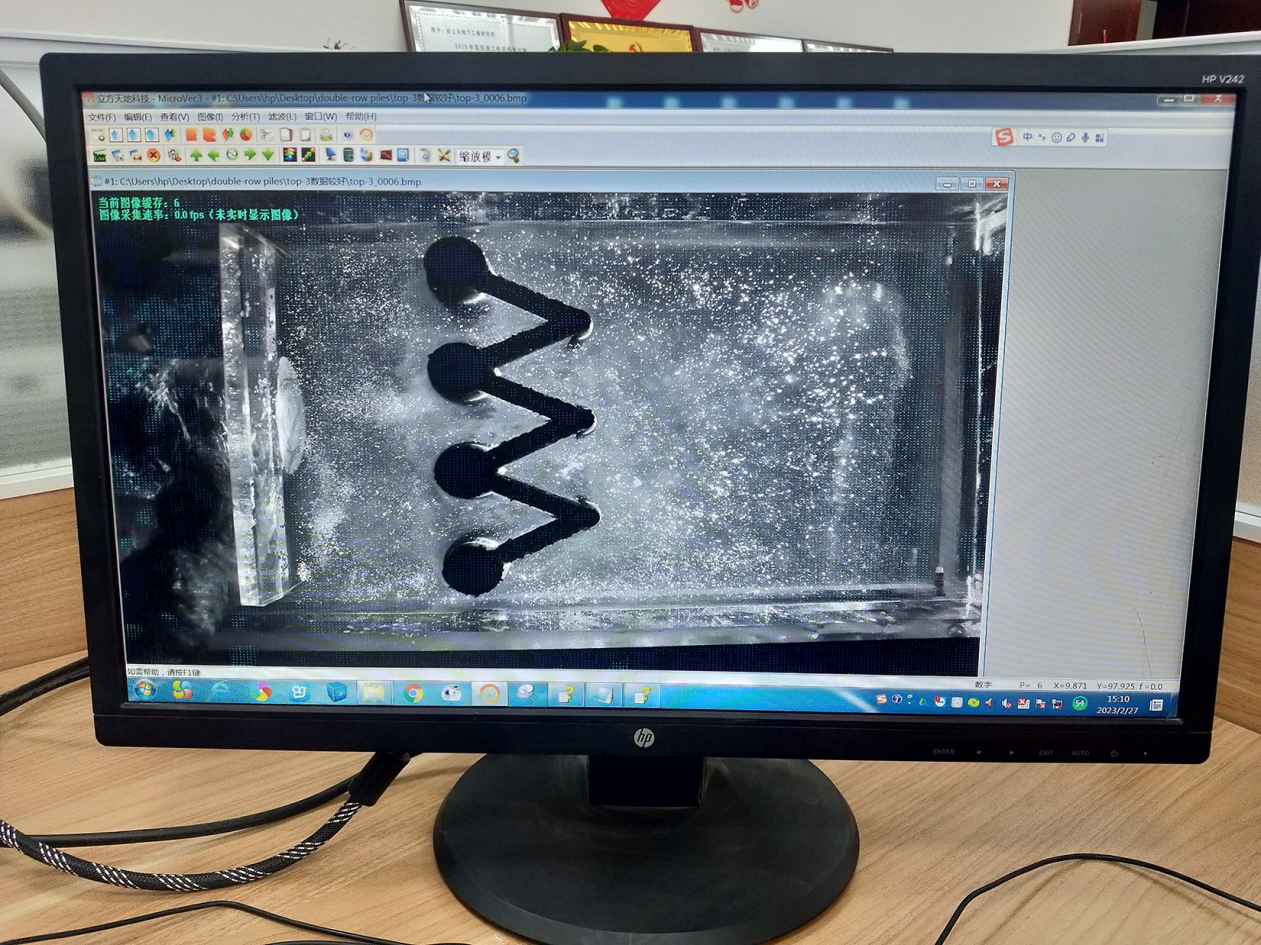
Interface of MicroVec software.

The PIV equipment and MicroVec software used in this study were provided by Beijing Cubic Space Science and Technology Development Co., Ltd. The spatial resolution of PIV is 0.5 mm. The data acquisition process involves continuous capture of images of the experimental observation surface using a camera, followed by processing via MicroVec software (Fig 8).

### 3.7 Vibration isolation platform

The purpose of the damping isolation platform is to reduce the interference of ground vibrations during the deformation of the model under load, especially for optical image acquisition, where slight vibrations can affect data accuracy. The damping isolation platform used in this study is the DRP18-12 standard dual-frequency damping isolation optical platform produced by Beijing Zhuo-Li-Han-Guang Instrument Co., Ltd. The DRP series is a new type of dual-frequency damping isolation optical platform that incorporates a dual-frequency damping isolation system with more reasonable rigidity, a smaller natural frequency, and better isolation performance. The structure of the DRP series includes a P-type dual-frequency damping isolation system, integral welded brackets, and standard stainless steel table tops. The overall height is 800 mm and is divided into two parts: the tabletop and the bracket.

**Fig 9:**
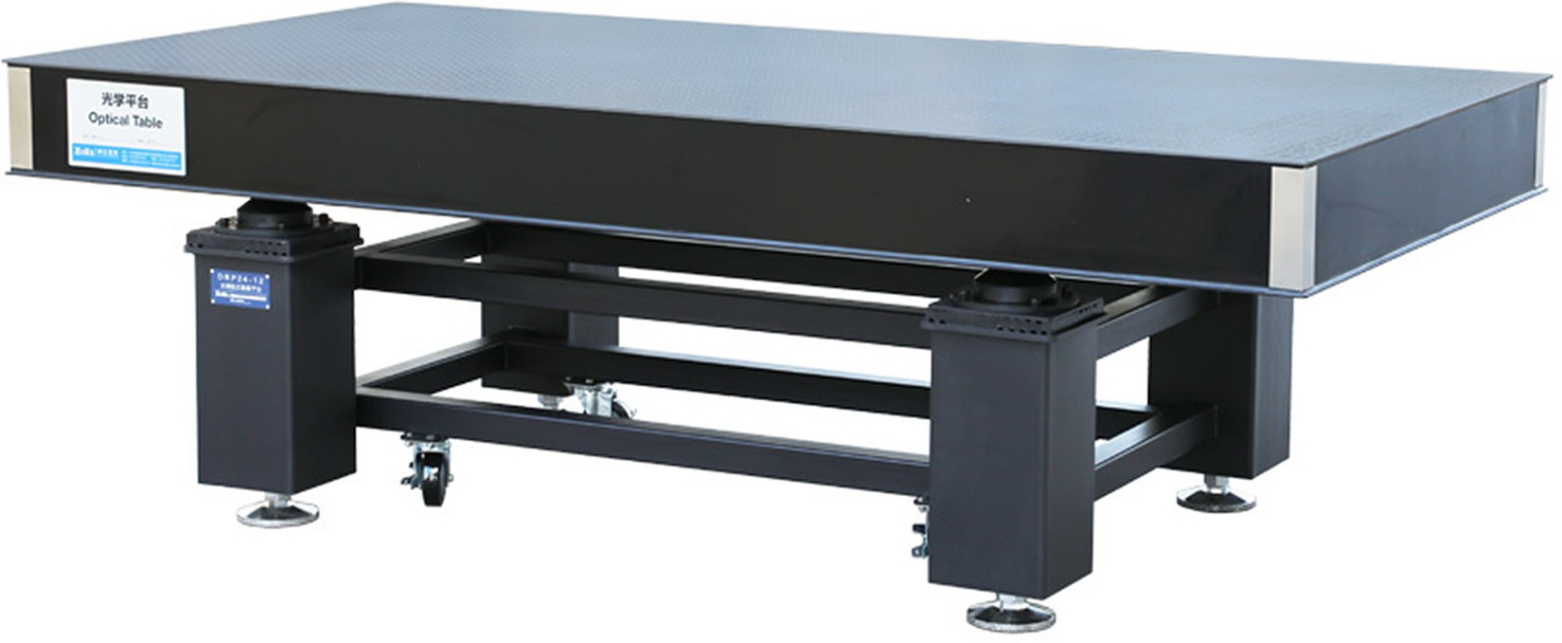
DRP18-12 damping isolation platform.

The dimensions of the isolation platform are 1800×1200×800 mm, with a tabletop thickness of 200 mm and a self-weight of 302 kg. The cross-section of the support legs is 200×200 mm, and the bracket has a load capacity of 1300 kg (Fig 9).

## 4 Mechanical properties of 3D-printed anti-slide piles

### 4.1 Test instrument

**Fig 10:**
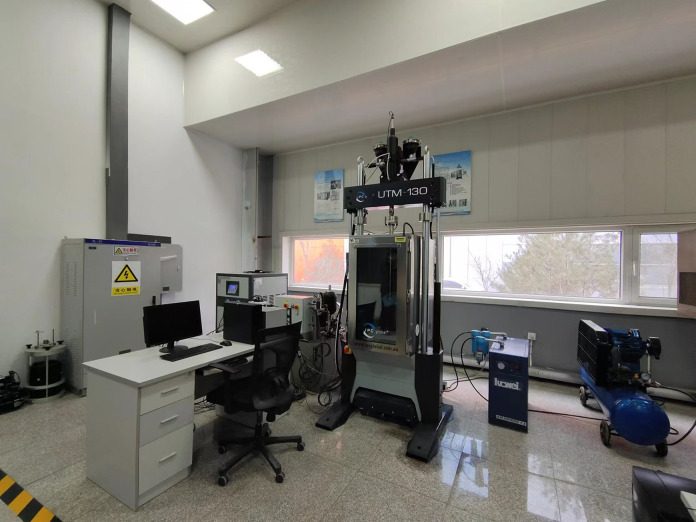
UTM-130 testing system.

The UTM-130 Servo-Hydraulic Dynamic Testing System (Fig 10) is manufactured by IPC, Australia; its static and dynamic loading capacities are 130 kN and 100 kN, respectively. The system utilizes UTS pneumatic-hydraulic servo closed-loop control, enabling testing and analysis of materials under various conditions, including temperatures ranging from -25 to 70°C. Loading tests and analyses, such as tension, compression, bending, shearing, penetration, and fatigue tests, can be conducted on this equipment. This equipment was used in this study to conduct strength tests of 3D-printed anti-slide piles.

### 4.2 Test program

**Fig 11:**
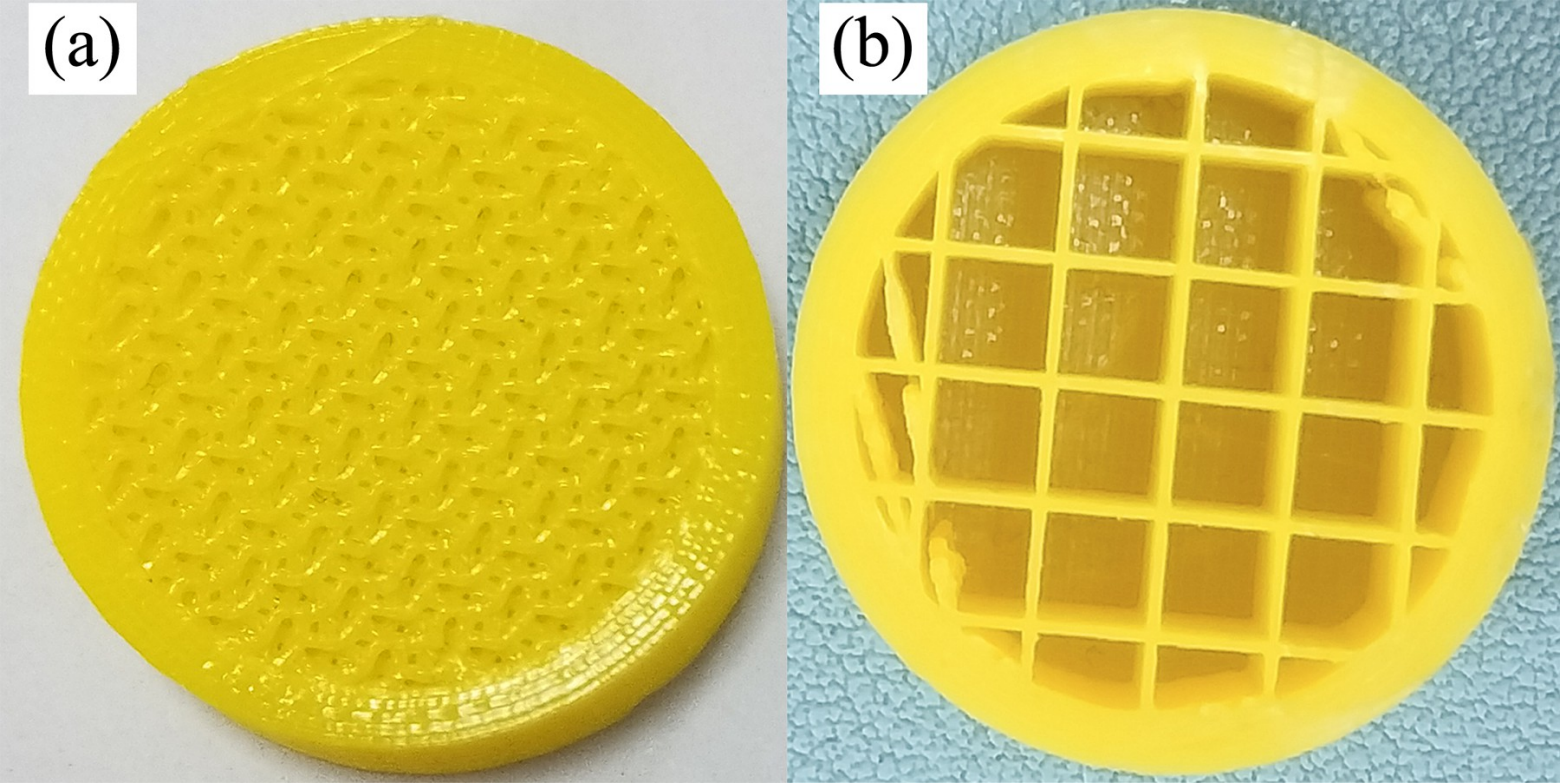
Triangular and square infill patterns for 3D printed anti-slide piles: (a) triangular; (b) square.

**Fig 12:**
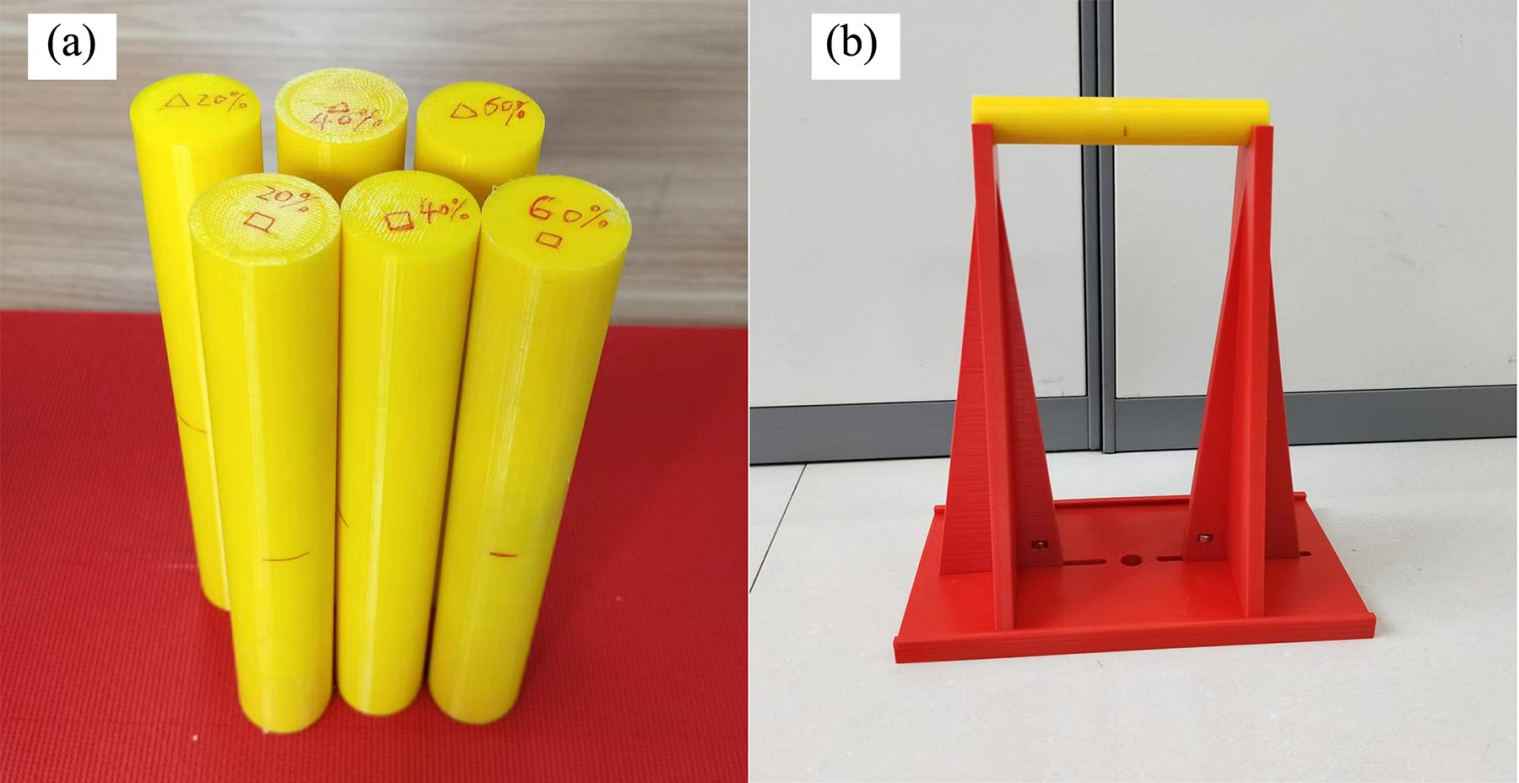
3D printed anti-slide piles (a) and experimental fixture (b).

A total of 6 anti-slide piles were printed with two types of infill patterns, namely, triangular and square patterns, each with infill ratios of 20%, 40%, and 60% (Fig 11). The dimensions of the anti-slide piles were 160 mm in length and 25 mm in diameter. Three-point bending tests were conducted on the anti-slide piles via the UTM-130 testing system (Fig 12A) to investigate the mechanical properties of the 3D-printed anti-slide piles. Owing to the absence of specialized three-point bending test fixtures in the UTM-130 testing system, we printed a experimental fixture using PLA, a kind of 3D printing material shown in Fig 12B.

### 4.3 Test results

#### 4.3.1 Analysis of deformation in three-point bending tests

**Fig 13:**
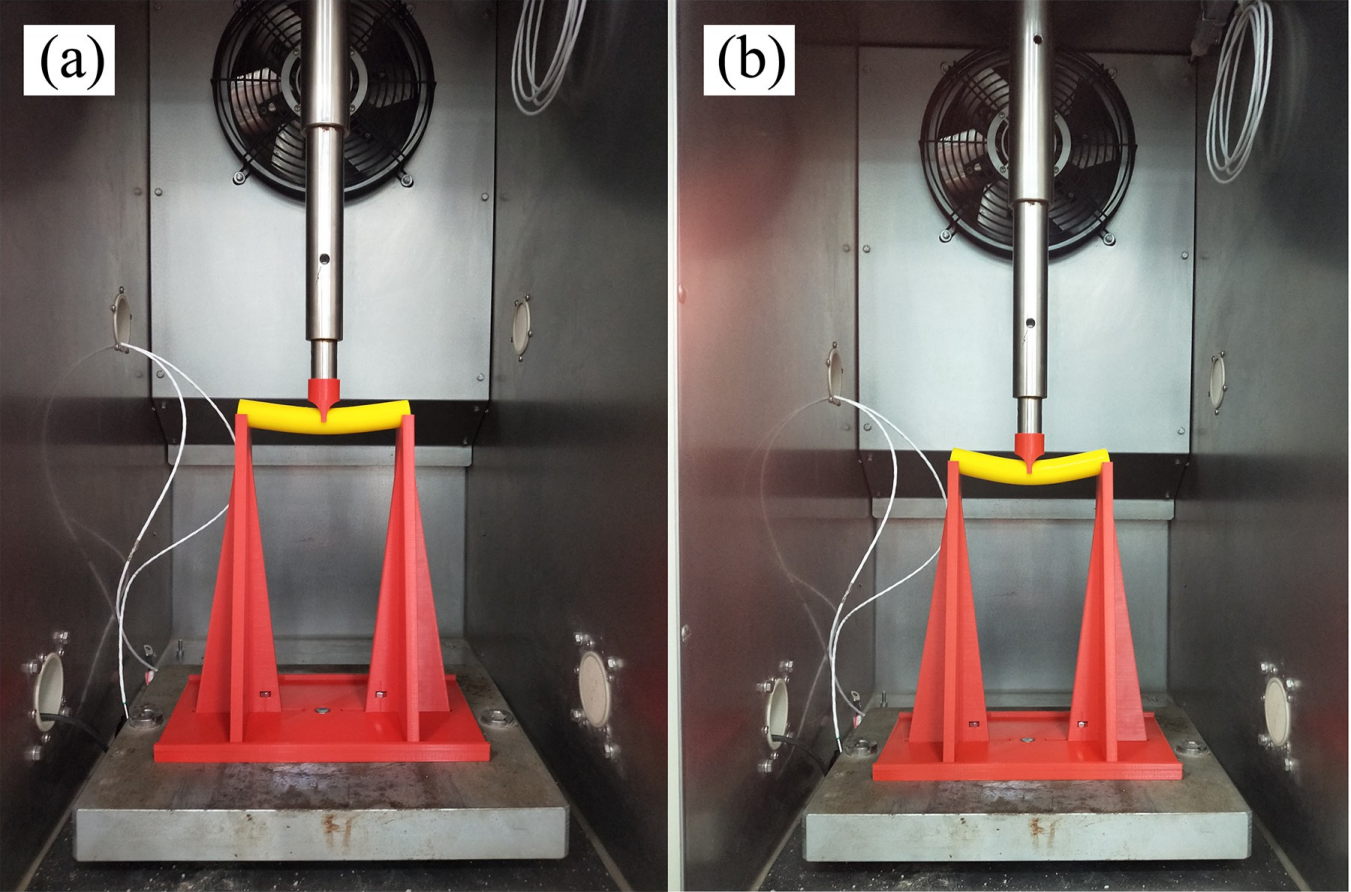
Photograph of 3D printed single-pile in three-point bending tests: (a) initial deformation: (b) final deformation.

A series of three-point bending tests were conducted to study the mechanical properties of single 3D-printed anti-slide piles. An experimental photograph is shown in Fig 13. During the loading process of the experiment, the 3D-printed piles exhibited excellent mechanical performance. Under the condition of a 20 mm loading displacement, the 3D-printed anti-slide piles did not show any signs of fracture, and deformation was uniform. After the experiment concluded, the piles were able to rebound and return to their original shape.

#### 4.3.2 Influence of the infill ratio and pattern on the mechanical properties of 3D-printed anti-slide piles

**Fig 14:**
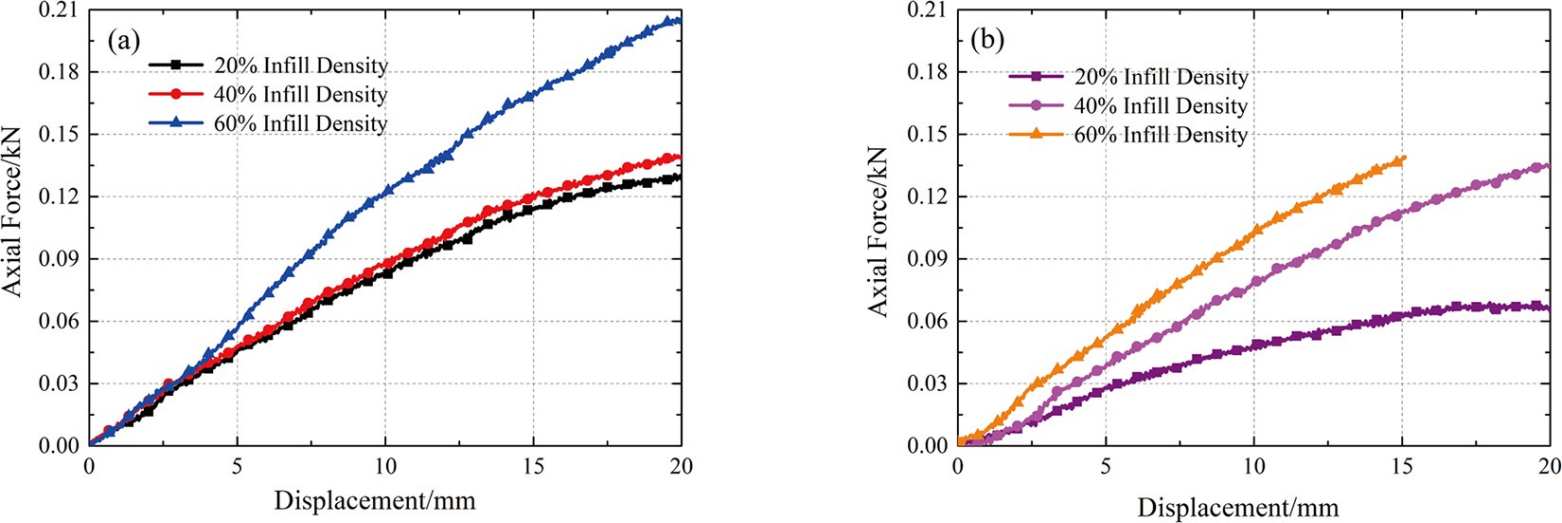
Relationship between axial force and displacement: (a) square infill pattern **(S1 File)**; (b) triangular infill pattern.

The curves between the axial force and displacement of the square and triangular infill patterns were drawn according to the test results (Fig 14). Overall, the curve is smooth, and there is no jump, which shows that the 3D-printed anti-slide piles have uniform and good mechanical properties.

**Fig 15:**
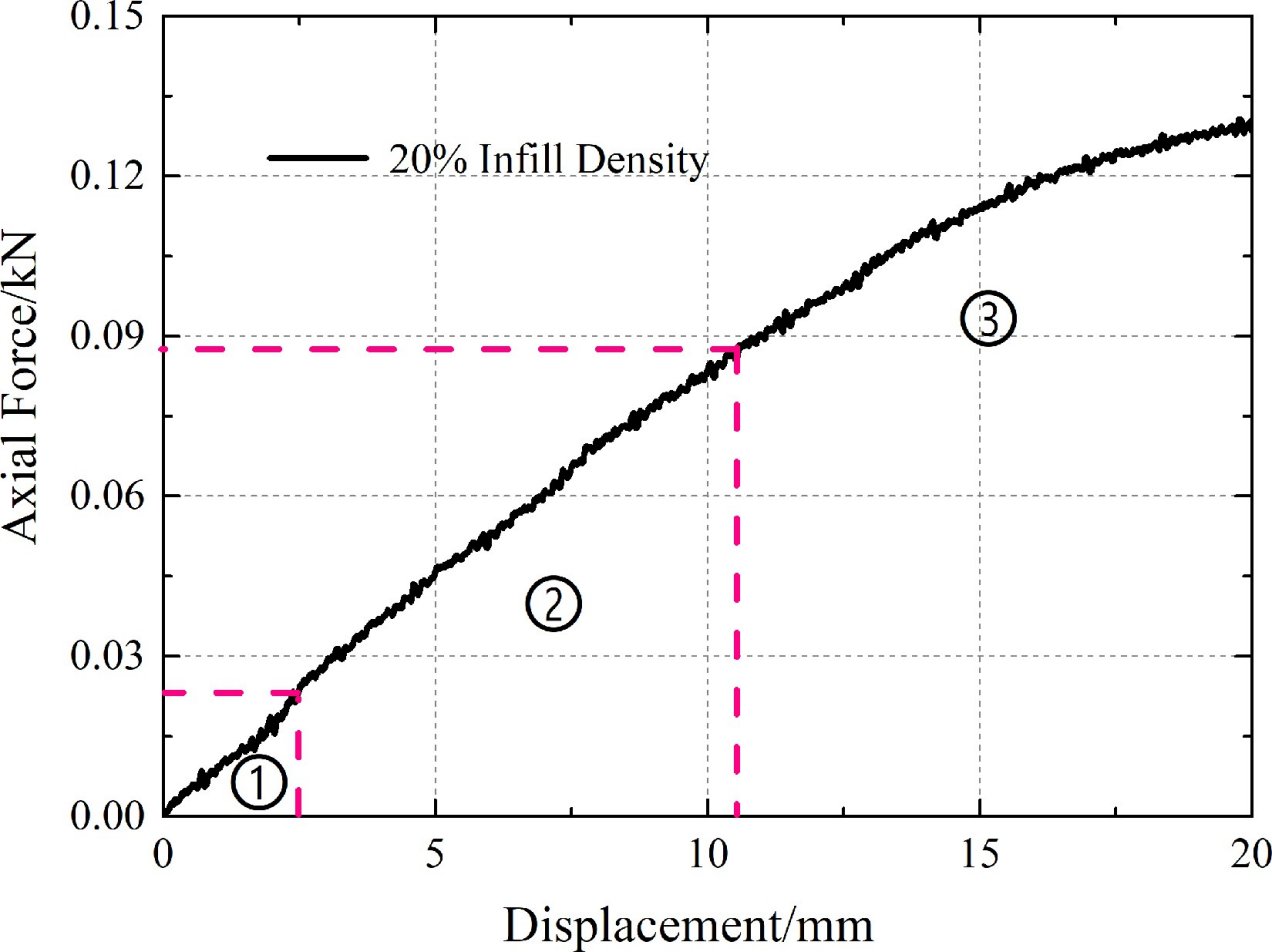
Deformation stages of 3D printed anti-slide pile.

The deformation stage of the 3D-printed single-row anti-slide pile was plotted by observing the curve between the axial force and displacement (Fig 15). This curve is in the form of a circular arc, which can be divided into three deformation stages: ① a compaction stage, in which the deformation is generated when the loading bar just makes contact, and the deformation stage is relatively short and then transforms into the next deformation stage; ② an elastic deformation stage, in which the curve is nearly straight, and the cause of deformation is the tensile deformation of the 3D printing material; and ③ a plastic deformation stage, in which the curve gradually slows down, and this damage is produced by the fusion between the filaments of the 3D printing material. The maximum displacement set for this test was 20 mm, and the 3D-printed anti-slide pile did not fracture, so the deformation phase was not present in its entirety.

According to the above deformation stage division, the calculation formula for defining the bending strength of the elastic deformation stage is as follows:

$$S=F_{n}/D_{s}$$ (1)

where ***S*** is the bending strength, ***F***_***n***_ is the axial force and ***D***_***s***_ is the vertical displacement.

**Fig 16:**
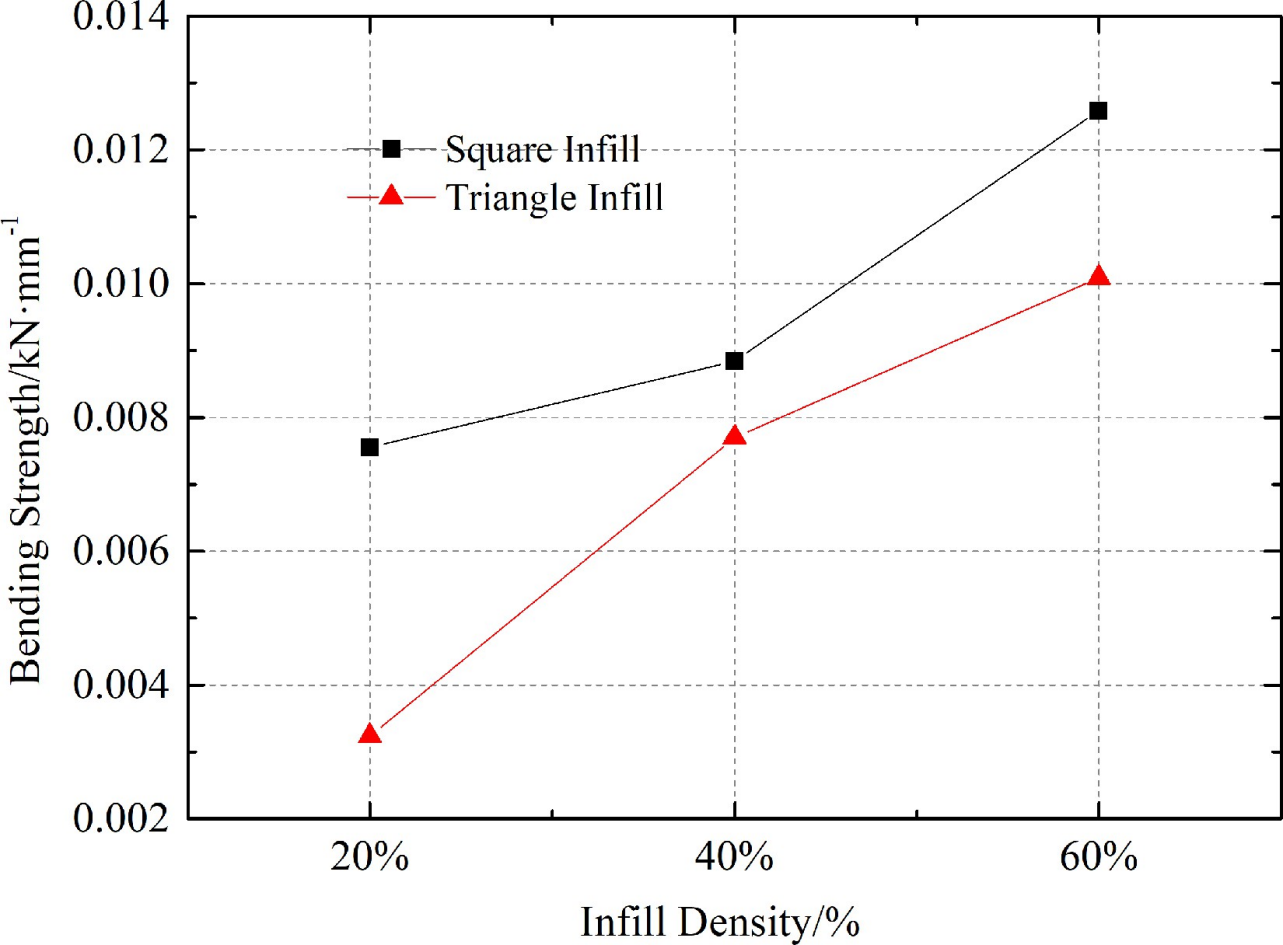
Relationship between bending strength, infill pattern and infill density.

The curve of the bending strength of 3D-printed anti-slide piles with different infill patterns and densities according to *Eq* (1) is shown in Fig 16. The bending strength of 3D-printed anti-slide piles under two infill patterns gradually increases with increasing infill density. Under the same infill density, the bending strength of square infill is greater than that of triangular infill. When the bending strength of the anti-slip pile is too high, the displacement of the anti-slip pile is not obvious. When the bending strength of the anti-slip pile is too low, the displacement of the anti-slip pile is too high because the simulation effect of the test is unfavorable. After the test comparison, an anti-slip pile with a printing density of 40% of the triangular infill pattern is selected.

## 5 Model test procedure

**Fig 17:**
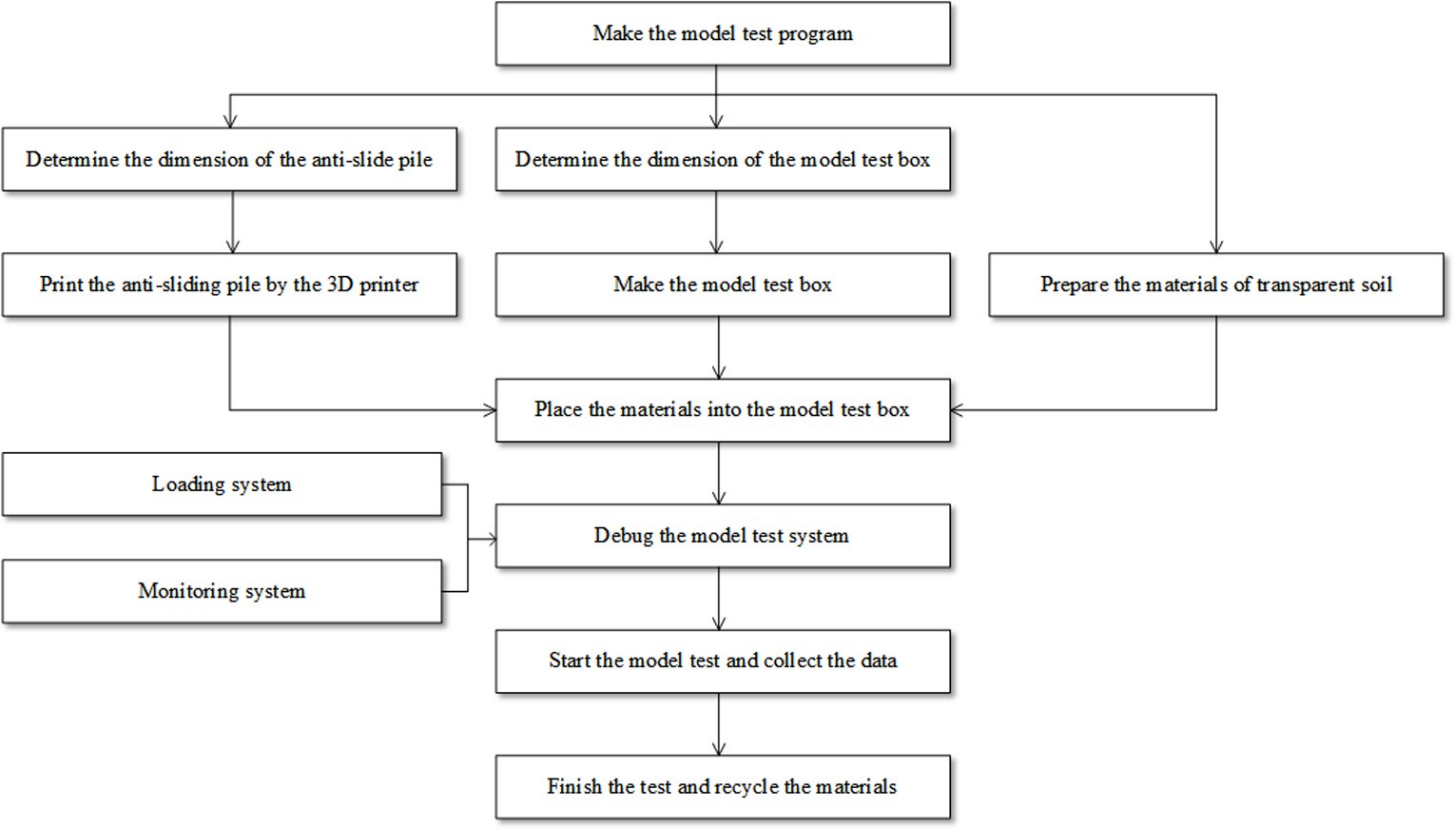
The flowchart of model test.

The procedure of the model test method developed in this paper is shown in Fig 17, and the model tests were carried out step by step as follows:

**Step 1:** According to the model test program, determine the dimensions of the anti-slide pile, including the length of the embedded and free sections and the cross-sectional shape.

**Step 2:** 3D print the anti-slide pile. First, a model of the anti-slide pile is created using the modeling software. Then, the model is sliced using the software provided by the 3D printer to determine the infill pattern and density of the printed anti-slide piles.

**Step 3:** Make the test model box. The dimensions of the model box, the thickness of each acrylic sheet, and the size of the holes drilled in the acrylic sheets were determined based on the dimensions and cross-sectional shape of the anti-slide pile. The various parts of the model box are bonded together and placed on a vibration isolation platform.

**Step 4:** Test the loading system. Open the jack piston.

**Step 5:** Place the ultrathin hydraulic jack, loading plate, and anti-slide pile inside the model box. Prepare the experimental model materials and transparent soil, by mixing silica particles, dodecane, and white oil in certain proportions inside the test model box. Then use a glass rod to mix it, eliminating any larger air bubbles in the transparent soil.

**Step 6:** Position a soil pressure cell at the front of the loading plate to record the thrust pressure data.

**Step 7:** Test the PIV system. Turn on the laser and the camera to ensure that deformation images can be captured.

**Step 8:** Start testing. Collect various types of data and images, including soil pressure cell data, deformation images, etc.

**Step 9:** Finish the test. The model test materials are recycled and all components are organized and placed in designated locations for convenient use in future tests.

## 6 Model test program and test results

### 6.1 Model test program

**Fig 18:**
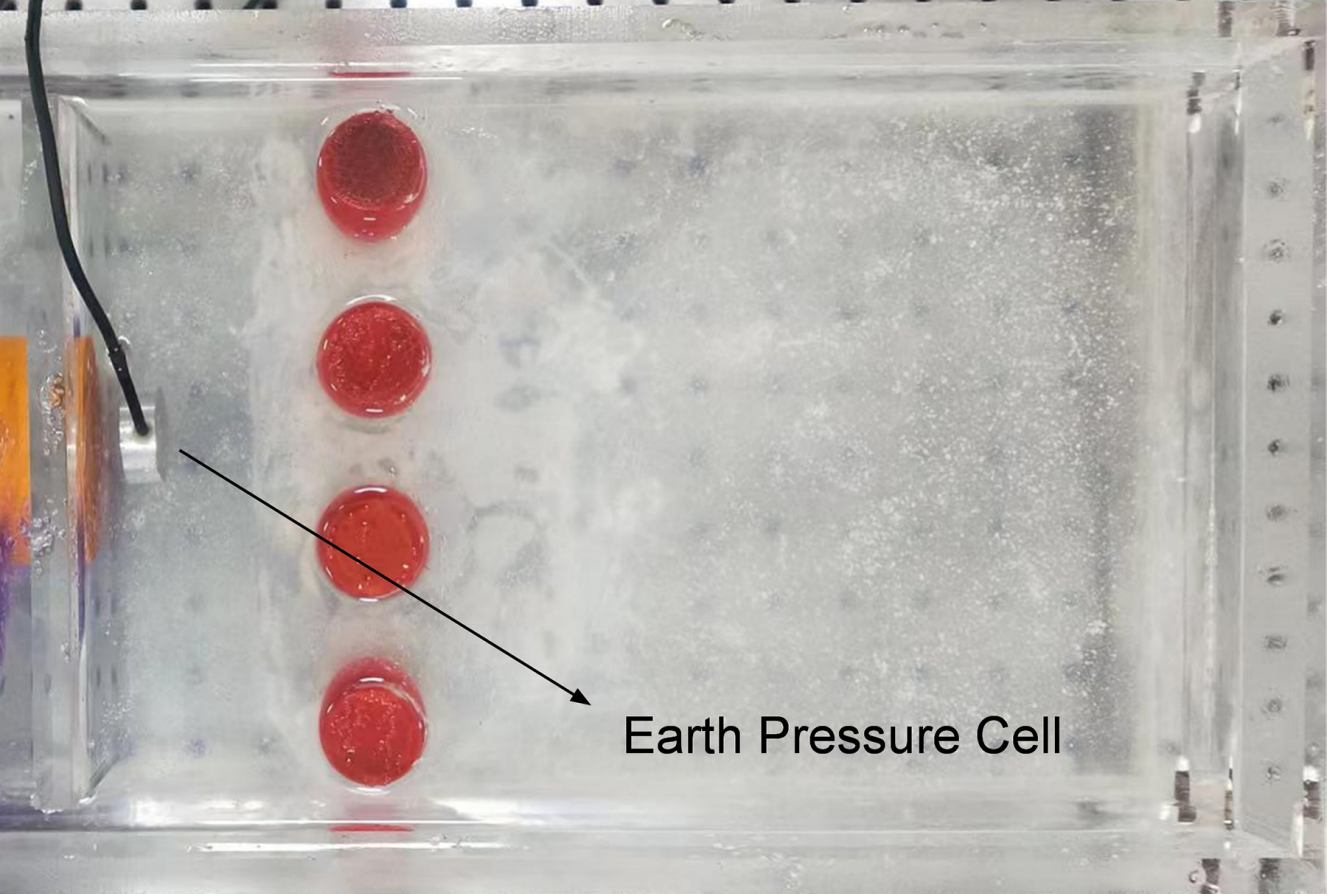
Single-row anti-slide pile model test.

**Fig 19:**
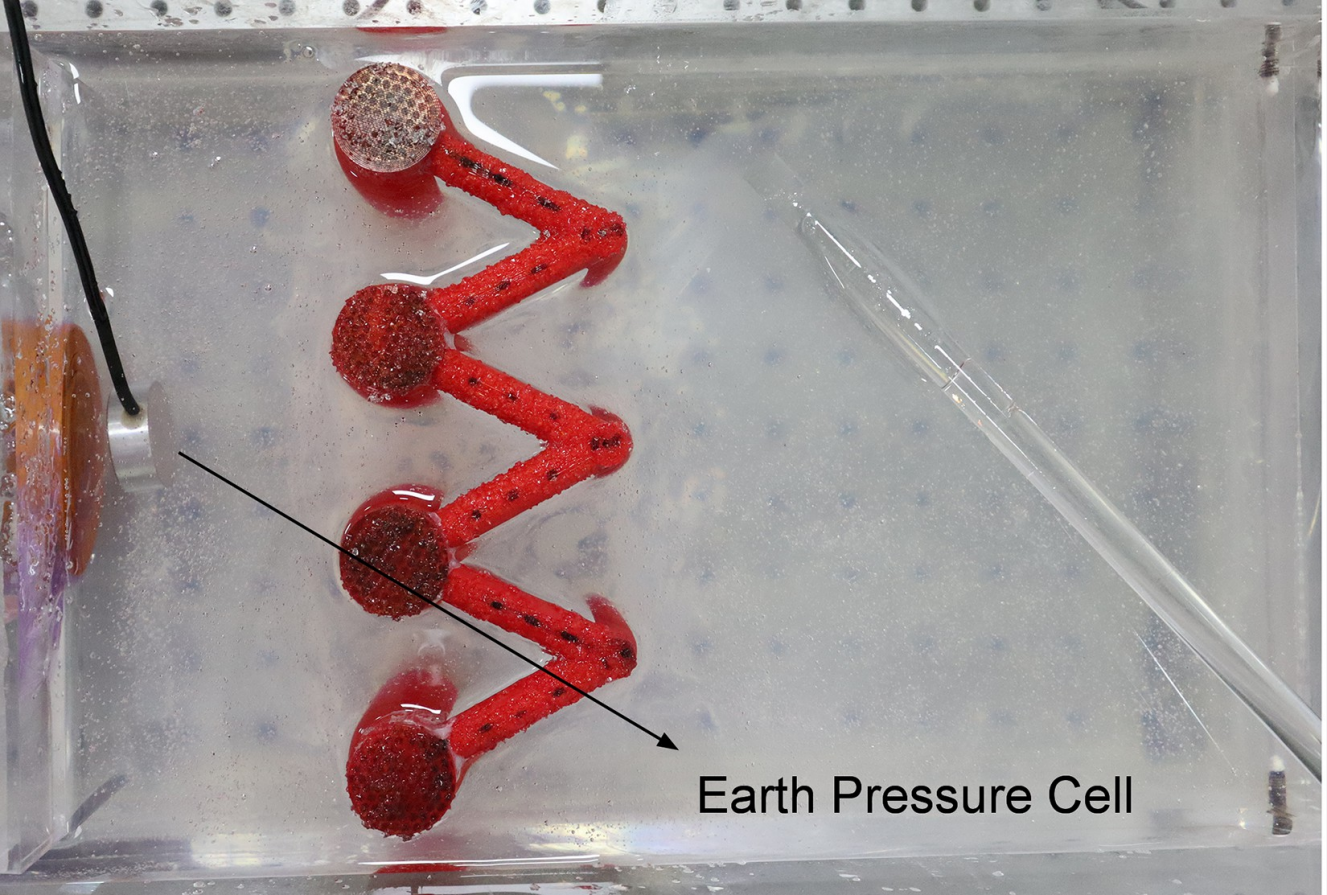
Double-row anti-slide pile model test.

Using the model test system mentioned above, two sets of model tests of each pile type, i.e., single-row anti-slide piles and double-row anti-slide piles, were carried out to compare the support effect (Figs 18 and 19), to study the anti-slide mechanism and the deformation effect of the soil body around the piles.

The earth pressure cell buried in front of the loading plate of the model test box (Figs 18 and 19) is used to collect thrust pressure data. The thrust pressure can be calculated from the strain data (*με*) collected by the soil pressure cell according to *Eq*. (2).

P=με·K(K=0.229428KPa/με) (2)

where ***K*** is the earth pressure cell parameter, ***με*** is the strain data of the earth pressure cell, and ***P*** is the thrust value.

**Fig 20:**
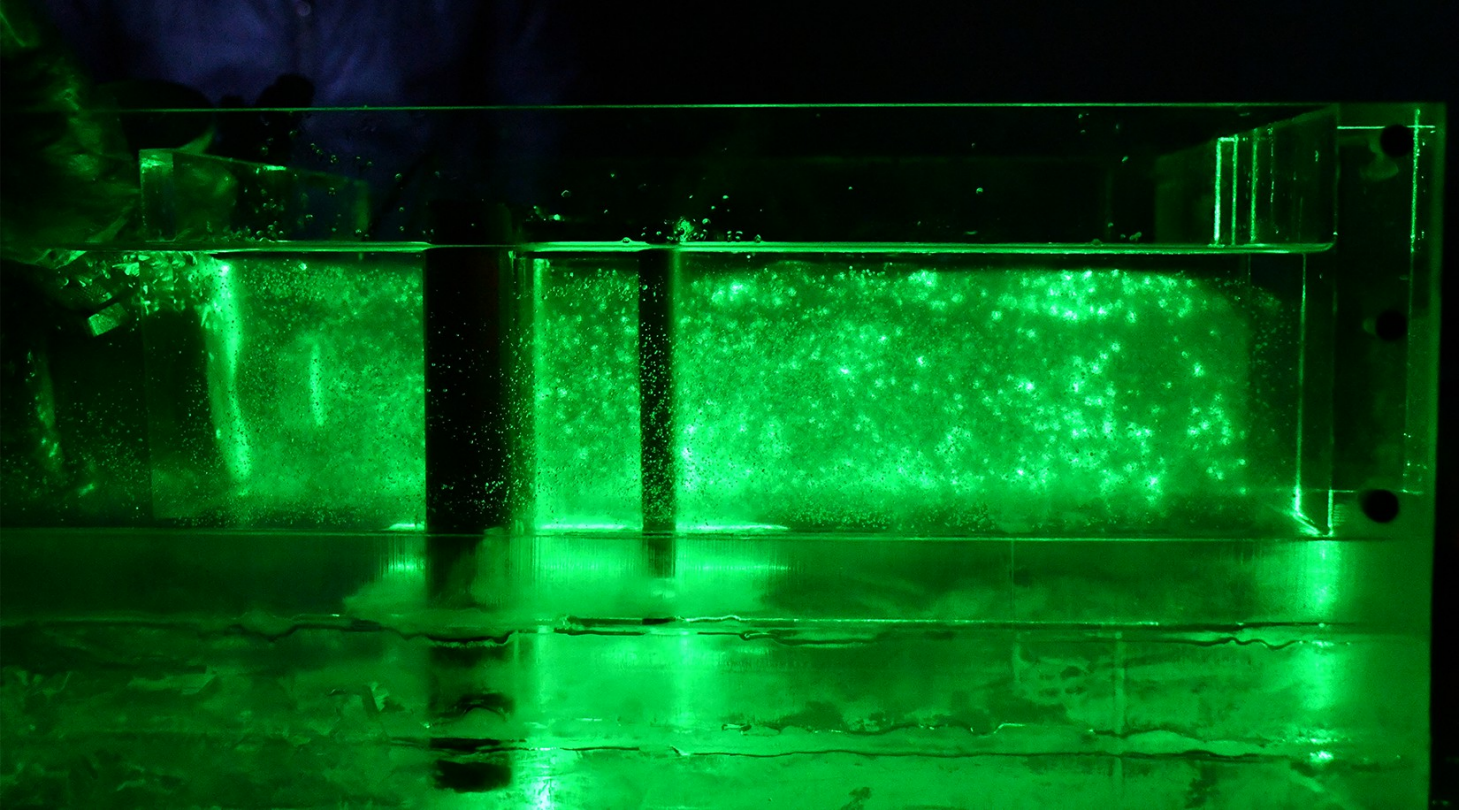
Speckle image from top using a vertical laser.

**Fig 21:**
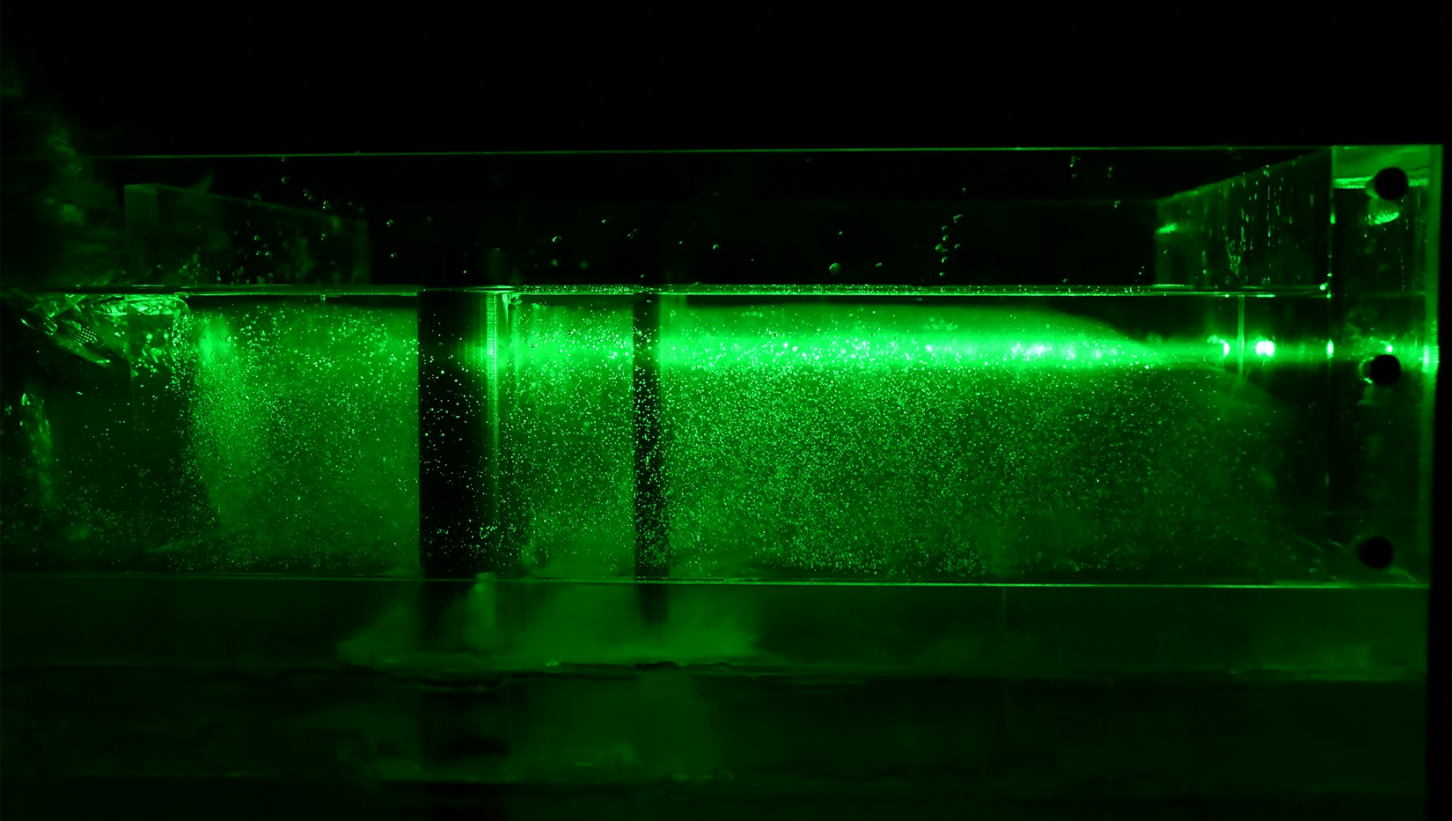
Speckle image from top using a horizontal laser.

**Fig 22:**
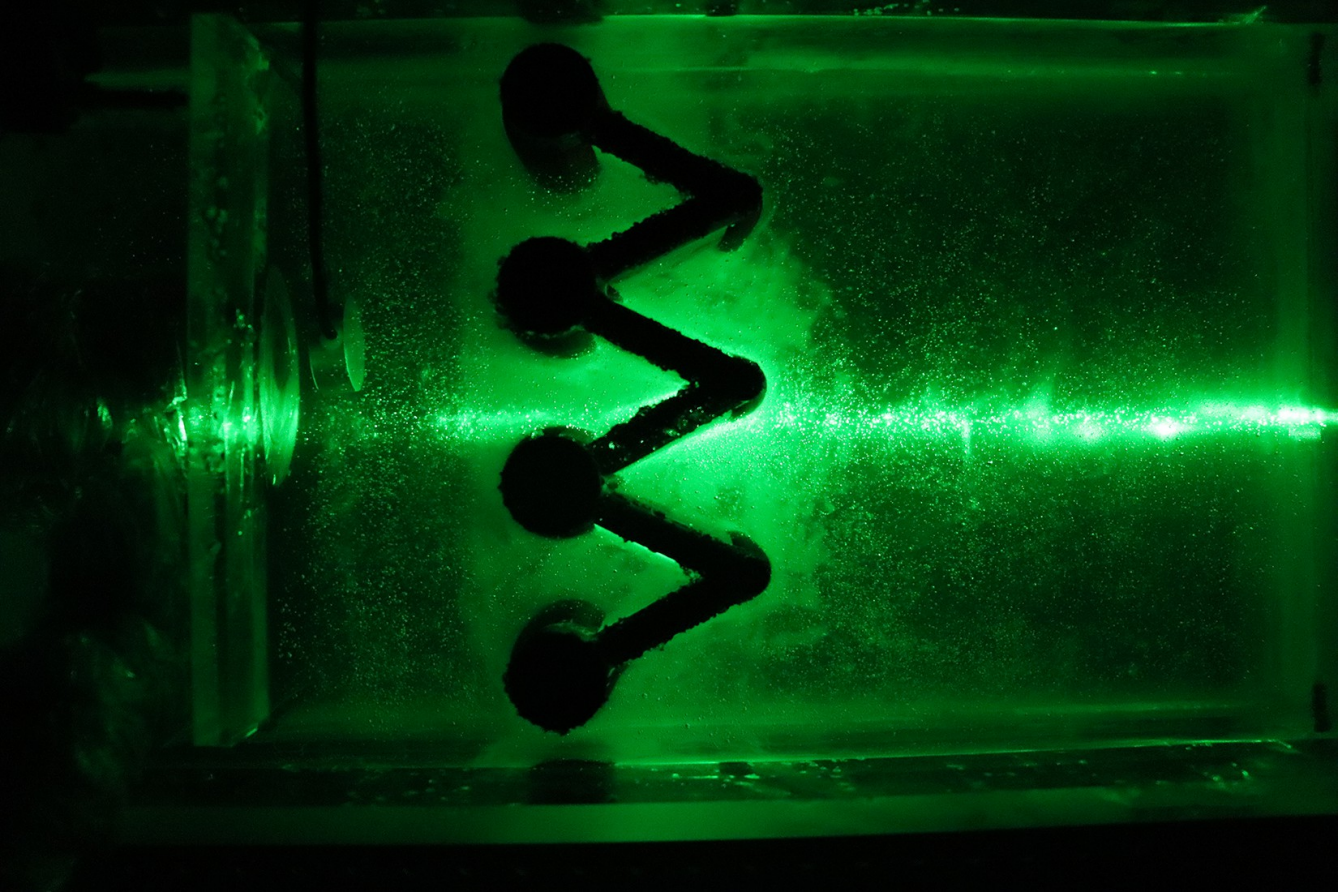
Speckle image from side using a horizontal laser.

**Fig 23:**
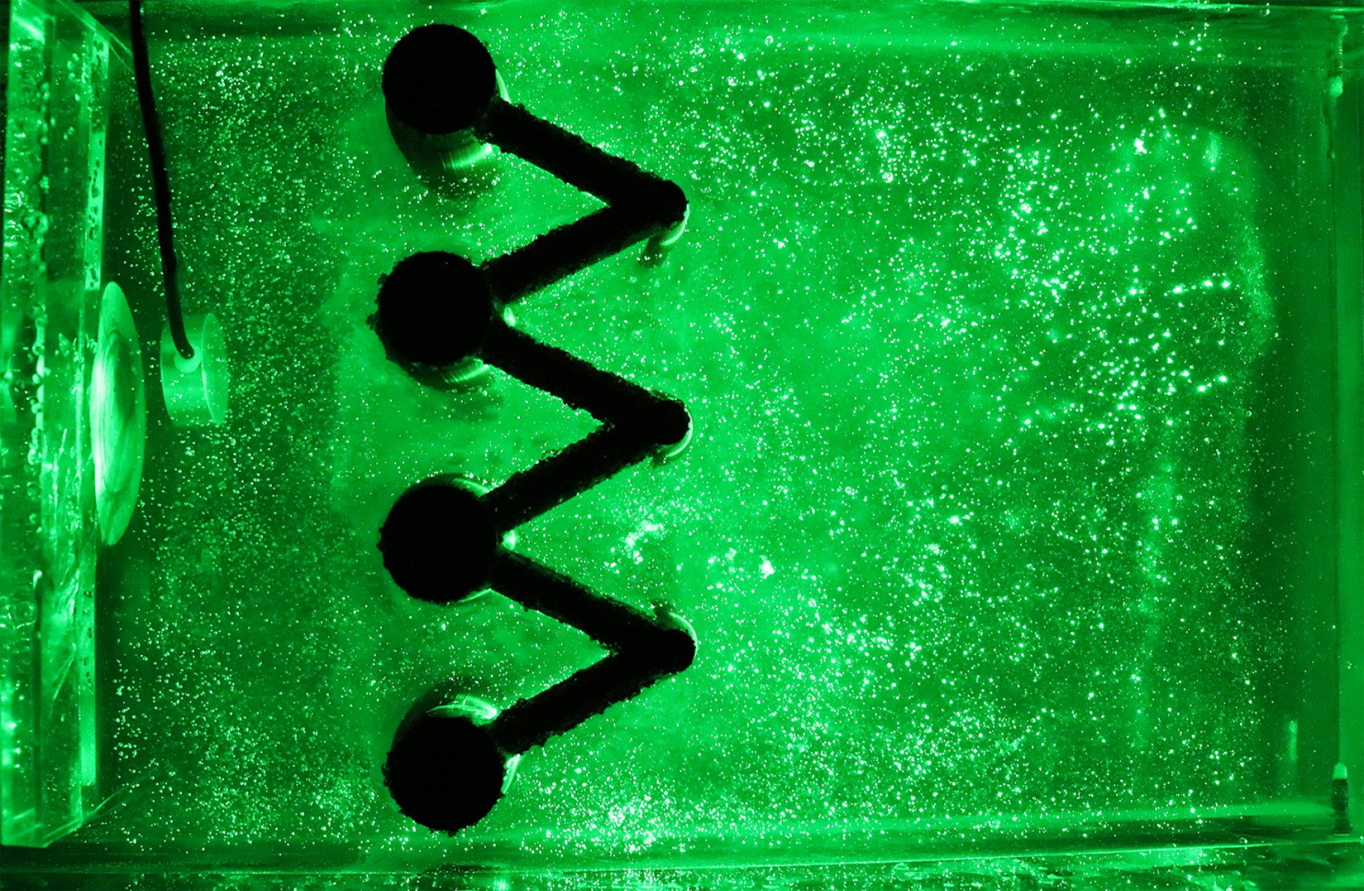
Speckle image from side using a vertical laser.

The laser can emit horizontally and vertically, depending on the orientation of the model box and the laser, and speckle images of different orientations can be collected, which are observed from the top (Figs 20 and 21) and side of the model box (Figs 22 and 23), respectively. Among the speckle images, the ones from the top obtained using a horizontal laser can be used by PIV to monitor the displacement of the soil surface at a depth of 1.5 cm from the surface of the transparent soil material (Fig 23), and the images from the side obtained using a vertical laser can be used by PIV to monitor the deep displacement of the soil (Fig 22).

### 6.2 Test results

#### (1) Thrust pressure

**Fig 24:**
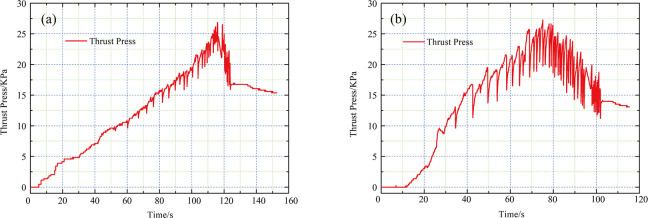
Thrust pressure data of model test: (a) single-row apile; (b) double-row pile.

The thrust pressure data of the single-row and double-row anti-slide model tests are shown in Fig 24. The maximum thrust pressures of the two model tests were approximately 27.5 KPa; therefore, the amount of thrust pressure is similar in both tests, and the difference in the supporting effects of the two types of anti-slide piles can be compared via these two model tests. After the thrust pressure reached the peak, that of the single-row anti-slide pile model test decreased to 12.5 KPa, whereas that of the double-row anti-slide model test decreased to approximately 15 KPa. The reason for this decrease may be as follows: first, the loading plate pushed the transparent soil forward, and the transparent soil material was bulged; at the same time, some of the soil between the piles was sheared out (Fig 29), which caused a gradual decrease in the thrust pressure recorded by the earth pressure cell. The loading plate could not continue to apply thrust, resulting in a gradual decrease, and eventually, the thrust pressure maintained a certain value. Second, the jack reached its maximum range and could not continue to provide thrust pressure.

Since a rocker-type hydraulic pump was used in this study, the large fluctuations in the thrust pressure data for both tests could have been caused by manually pulling and pressing the rocker; therefore, the thrust pressure data varied unevenly. The test operator attempted to press the hand crank of the jack at an almost constant rate to achieve stable thrust pressure data.

There are many ways to provide thrust pressure, and Xie et al. [23] developed a new stacking loading method by weight. This method can be used in the following tests.

In the future, the loading method can be improved over the electrohydraulic servo loading method, but the problem of miniaturization still needs to be solved.

#### (2) Displacement evolution of deep soil

**① Displacement evolution of deep soil in single-row anti-slide piles**

Test images of the displacement evolution of the soil deep in the single row anti-slide piles are shown in Appendix Ⅰ. The black and white background images are taken by the camera during the test process, while the colored arrows represent the displacement deformation vectors processed by MicroVec software. The displacement laws of the deep soil and the anti-slide piles are analyzed by observing the deformation vectors and the shadows of the anti-slide piles in the images. The law of displacement of the soil is presented below:

As shown in Appendix Ⅰ S1 and S2 in S1 Appendix, for the initial loading period, there was no significant deformation of the soil or pile, and the anti-slide pile remained approximately vertical. However, displacement was observed on the surface of the transparent soil (see Appendix Ⅰ S2 in S1 Appendix); this may be due to air bubbles inside the transparent soli rising to the surface under the loading pressure. Appendix Ⅰ S3 in S1 Appendix illustrates that as the thrust pressure increases, displacement occurs primarily in the upper area behind the pile, with only a small amount of displacement occurring on the soil surface in front of the pile. The anti-slide pile experiences slight deformation. Appendix Ⅰ S4 in S1 Appendix shows the deformation displacement of the soil after the pile has been extended to depth. The figure also shows a large area of soil displacement in front of the pile. However, the displacement of the anti-slip pile is not significantly different from that shown in Appendix Ⅰ S3 in S1 Appendix. Appendix Ⅰ S5–S10 in S1 Appendix show that as the thrust increases, the soil body deforms continuously, and the displacement distribution is similar to that inAppendix Ⅰ S4 in S1 Appendix. The deformation of the anti-slip pile remains minimal. From Appendix Ⅰ S11 to S18 in S1 Appendix, the thrust pressure increases, causing the soil behind the pile to be extruded. The displacement rate also tends to increase. The deep soil behind the pile clearly distinguishes between light and dark, and there seems to be a “***displacement triangle***”. Within this triangle, the displacement rate is nearly zero. In the upper part of the triangle, the distribution of soil displacement is evident. As shown in Appendix Ⅰ S11–S18 in S1 Appendix, the size of this area gradually increases. The tilt angle of the anti-slide pile reaches its maximum at this stage. In Appendix Ⅰ S19 in S1 Appendix, the hydraulic jack reaches its maximum range, and the thrust pressure stops increasing. The soil behind the pile no longer generates displacement increments, but a small amount of displacement is still generated in the upper soil in front of the pile. At this point, without any displacement increment, the maximum inclination angle of the anti-slip pile is approximately 8° (see Appendix Ⅰ S20 in S1 Appendix).

**Fig 25:**
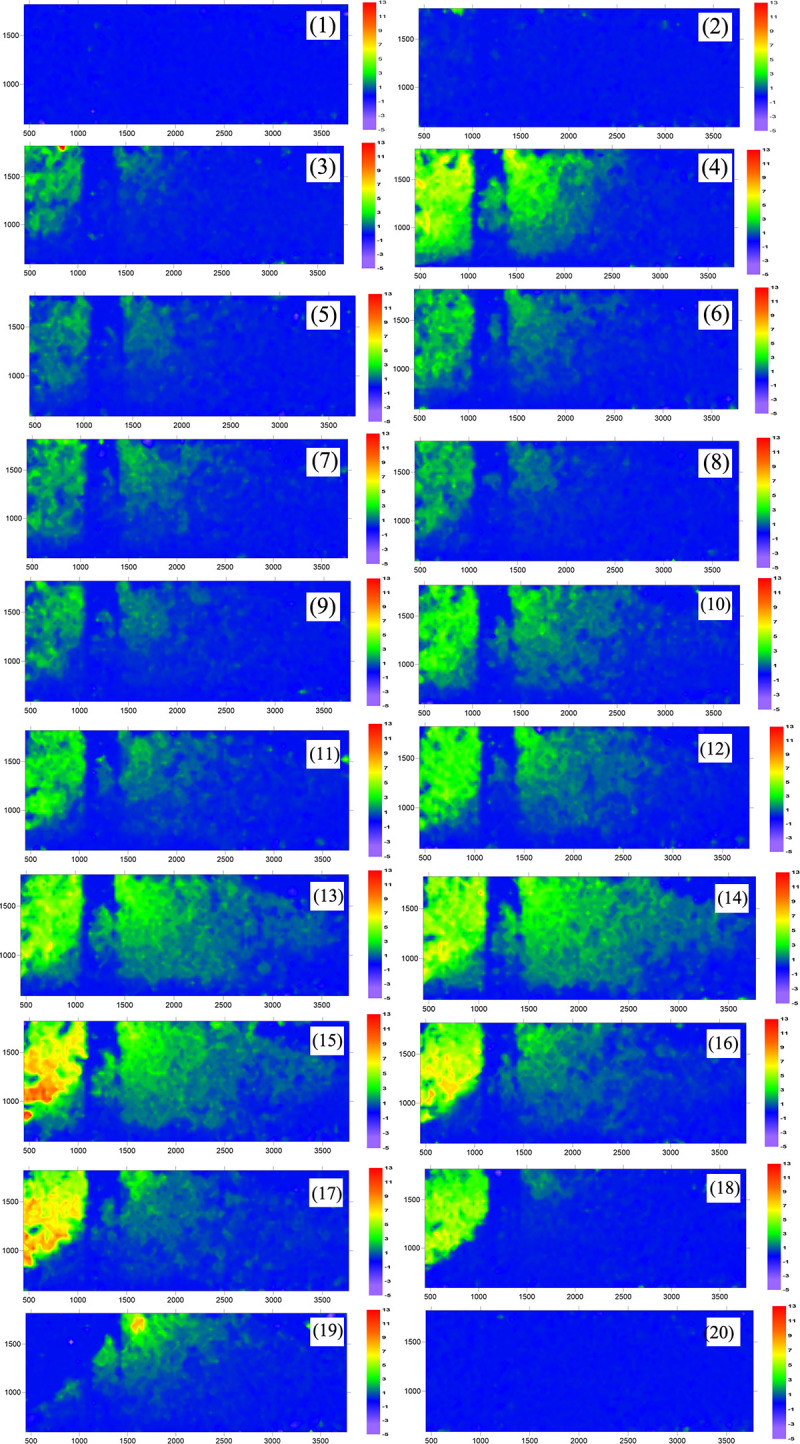
Displacement rate nephogram of soil in deep (single row anti-slide pile). (The 20 figures are arrange according to the test time).

Fig 25 shows the displacement rate contour plot of the single-row anti-slide pile. The nephograms were formed by exporting deformation data from figures in Appendix Ⅰ through MicroVec software and processing it with Surfer12 software. This allows for a more intuitive judgment of the size of the displacement vector and the deformation range of the displacement rate. However, the software cannot identify the displacement of the anti-slide pile because the anti-slide pile body does not generate a speckle field. In the following test, we can place fluorescent markers on the body of the anti-slide pile or print it with fluorescent material to identify its displacement. The soil displacement field in Fig 25 is more significant than that in the figures in Appendix Ⅰ. As shown in Figs 25(10)—(16), the displacement in front of the anti-slide pile extends more widely. The soil displacement was nearly distributed along the slip surface behind the pile. The “displacement triangles” appear to be more obvious in Fig 25(16). Based on the magnitude of the displacement vector, the pile‒soil interaction exhibits continuous deformation with a uniform deformation rate during the initial stage of the test. However, in the final stage of the test, there is a significant increase in the displacement vector, particularly in the middle‒upper part of the soil behind the pile. This suggests that the continuous deformation of the soil body is not sustainable and that the soil undergoes shear damage, with shearing occurring along the upper edge of the “displacement triangle”, causing the soil surface behind the pile to bulge and the soil between the piles to shear. This will be further explained in the following sections. Fig 25(19) shows no displacement of the soil behind the pile toward the end of the loading, whereas there was still displacement of the soil surface in front of the pile. This may be a hysteresis phenomenon of thrust transmission.

**② Displacement evolution of the soil in deep double row anti-slide piles**

Appendix II S1-S5 in S2 Appendix shows the soil deformation of the model test of the double-row anti-slide pile. MicroVec software was used to iteratively process the collected soil displacement data, resulting in five images. The colored arrows in the figures indicate the direction and magnitude of the soil displacement vector.

Appendix II S1 in S2 Appendix illustrates the initial loading stage, where the soil remains undisturbed and the anti-slide pile shows no signs of deformation.

As shown in Appendix II S2 in S2 Appendix, the increasing thrust pressure applied in the model test causes significant displacement of the soil behind the back row of piles. Although some displacement occurs between the two rows of piles, it is much smaller in magnitude than that behind the back row of piles. The displacement of the double-row anti-slide pile body is not readily apparent at this time. The deep soil behind the back pile appears as a small “***displacement triangle***”, which is similar to the test result of a single row pile.

Appendix II S3 in S2 Appendix shows that as the thrust increases, the displacement vector is transmitted over a wide range, and displacement data are collected in more than half of the model box. The double-row anti-slide pile experiences only a small amount of tilt, demonstrating the effectiveness of this structure in providing support.

Appendix II S4 in S2 Appendix demonstrates that the soil in front of the two rows of piles remained stable and did not undergo any significant displacement or deformation with increasing thrust pressure, thus confirming the effectiveness of the two rows of piles in providing support. The soil behind the back anti-slide pile exhibits a “displacement triangle”, which confirms the absence of displacement in this area, which is similar to the test results for a single row pile. This “***triangle***” area is larger than that of the single-row pile, and the displacement vector points toward the surface of the soil, indicating that the double-row pile has a superior supporting effect. The displacement of the structure of the double-row anti-slide piles shows only a slight increase, further demonstrating their effectiveness. However, the deformation of the back row anti-slide pile is slightly more significant.

In Appendix II S5 in S2 Appendix, the thrust application causes the soil behind the back row piles to bulge, resulting in a tilt of approximately 3° for the structure of the double-row anti-slide piles.

In summary, compared with single-row anti-slide piles, double-row anti-slide piles have a better supporting effect on controlling soil deformation. The displacement of the top of the double-row anti-slide pile has also been effectively reduced by approximately 62.5%.

#### (3) Displacement evolution of soil at the surface

**① Displacement evolution of the soil at the surface of single-row anti-slide piles**

**Fig 26:**
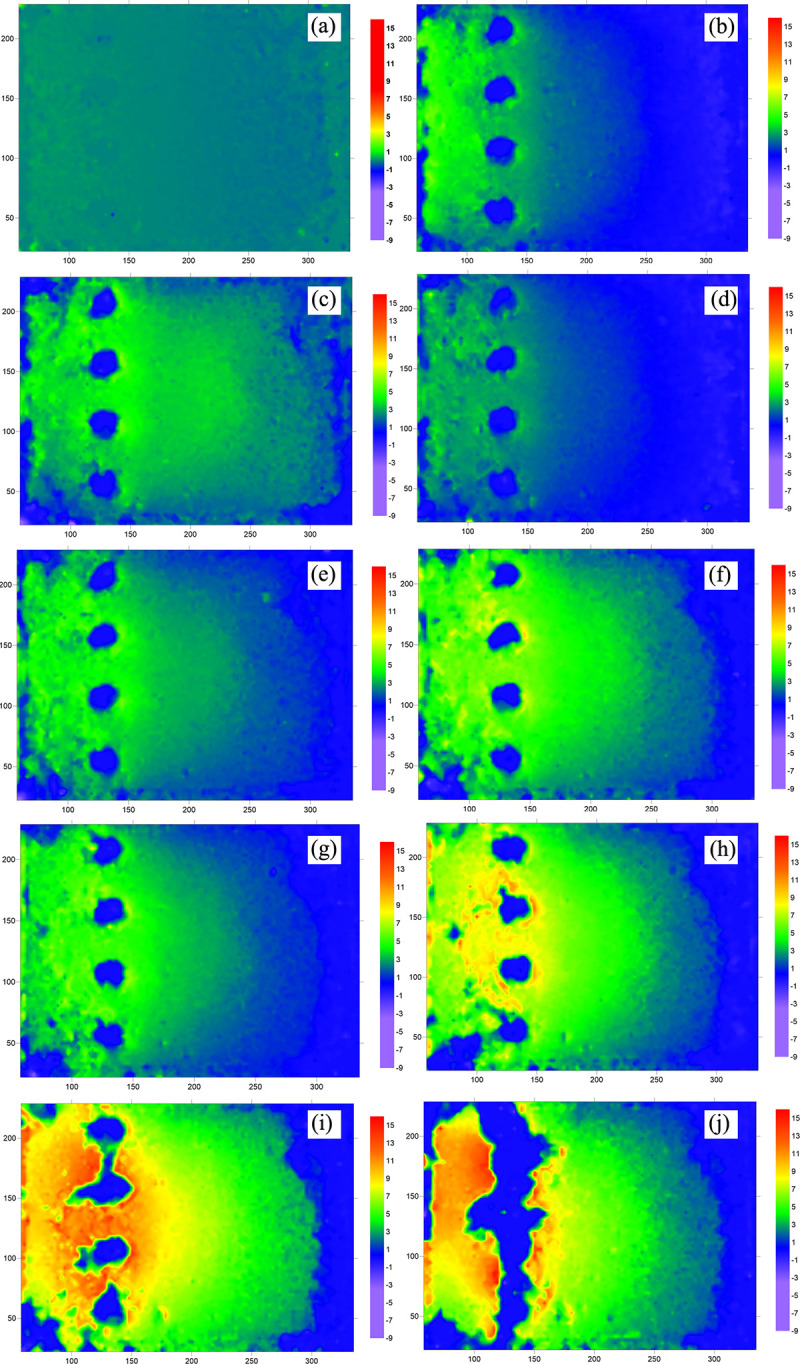
Displacement rate nephogram of soil at the surface (single row anti-slide pile). (The 10 figures are arrange according to the test time).

The evolution of soil displacement at the surface of a single-row pile is shown in Fig 26, which consists of 10 images arranged in chronological order, with colored shading representing the magnitude of the soil deformation rate. The displacement rate of the soil in Fig 26(A) is not obvious at the beginning of loading. The circular shading indicates the location of the top of the anti-slide pile. With increasing thrust pressure (Fig 26(B)–26(G)), the soil at the surface is displaced, and the displacement rate continues to increase. Its expansion is in the form of an arc, which is probably due to the influence of the boundary effect, which refers to the friction between the transparent soil and the sidewall of the model box that hinders the displacement of the soil mass. Fig 26(H) and 26(J) show a large displacement rate of the soil between the piles, indicating the phenomenon of extrusion of the soil between piles.

**② Extrusion of soil between piles (single-row anti-slide piles)**

**Fig 27:**
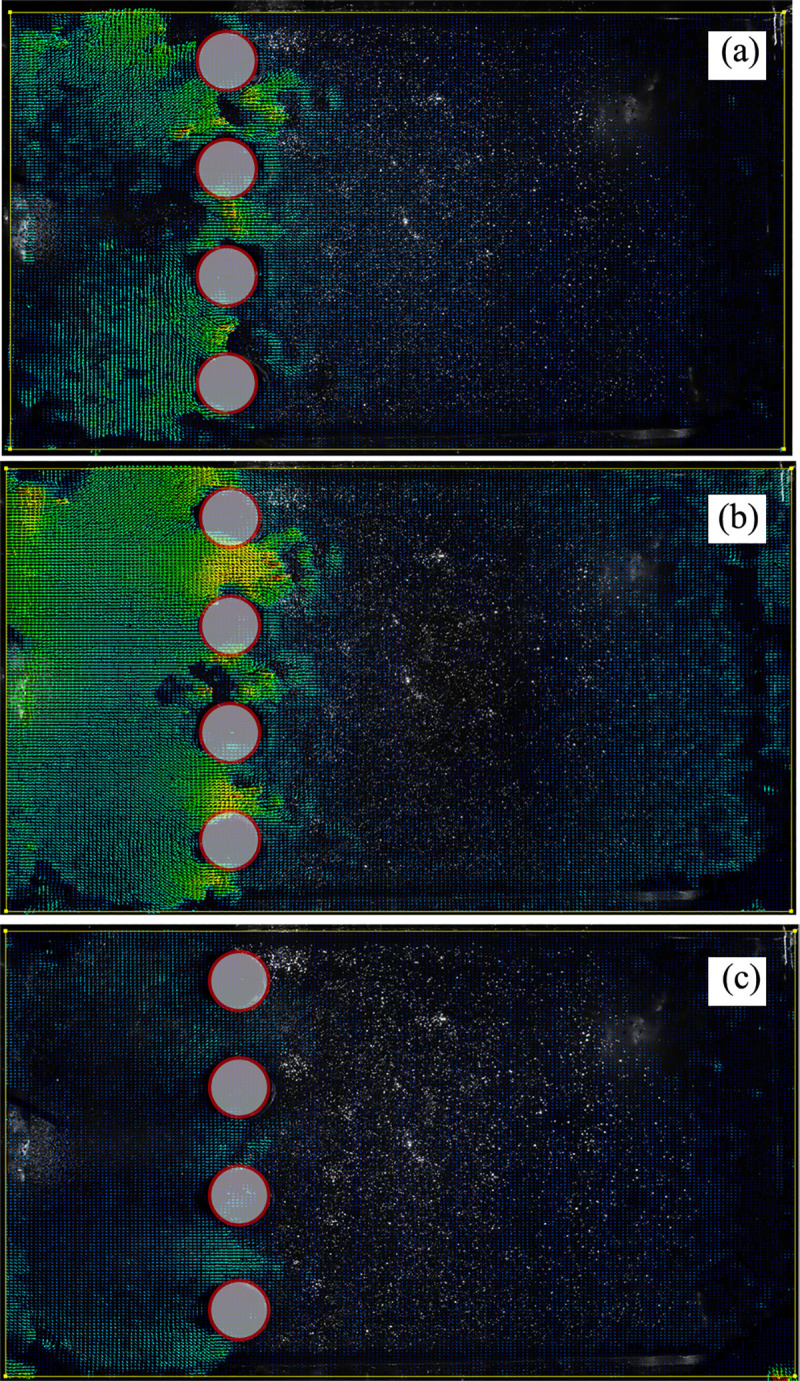
The extrusion of soil between single-row anti-slide piles. (The 3 figures are arrange according to the test time).

The extrusion of soil between single-row anti-slip piles can be identified (Fig 27). The three images in Fig 27 are arranged chronologically, and the red circle indicates the position of the top of the pile. No deformation is recognized in the red circle, whereas the soil is sheared around the anti-slide piles. As there is only one row of piles, the soil displacement is up to twice the diameter of the pile.

**③ Displacement evolution of the soil at the surface of double row anti-slide piles**

**Fig 28:**
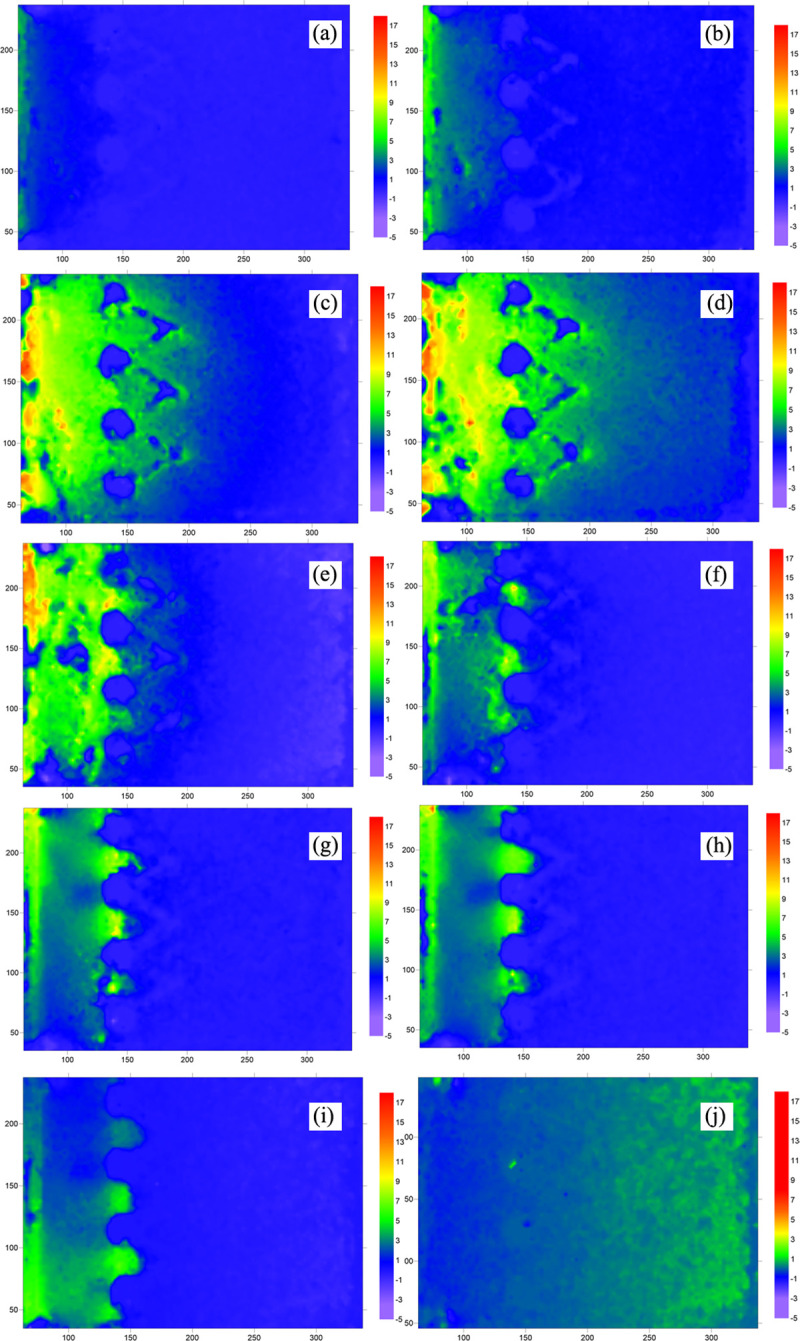
Displacement rate nephogram of soil at the surface (double row anti-slide pile). (The 10 figures are arrange according to the test time).

The displacement rate nephogram of the soil at the surface of the double-row pile is shown in Fig 28. At the beginning of thrust pressure application, displacement vectors were found in a small area at the edge of the loading plate (Fig 28(A)), and no displacement was observed at the other surfaces of the soil. In Fig 28(B), the thrust pressure is continuously applied. The soil is restrained by the anti-slip piles, and as a result, soil displacement gradually occurs behind the piles. The dark area represents the top of the double-row pile in the “V” shape. The soil surrounding the double-row pile moved with increasing thrust pressure (Fig 28(C)), but the extent of soil displacement was small, and the “V” shape of the double-row anti-slide pile was more obvious. Fig 28(D) shows that the thrust pressure has a more significant effect, causing the distribution area of displacement to gradually increase. However, the displacement distribution around the anti-slide pile remains uniform, indicating that the anti-slide pile can coordinate deformation with the soil. In Fig 28(E), the concentration of displacement at the edge of the loading plate indicates that the thrust pressure is still in effect. The increase in displacement in front of the pile decreases at this point, and the area of incremental displacement is concentrated behind the pile and between the two rows of piles. The soil may have bulged at the top of the pile, and there may have been extrusion of the soil between the piles. In Fig 28(F)–28(I), there is no significant displacement at the loading plate, the thrust pressure stops increasing, and the displacement area of the soil behind the pile gradually dissipates.

**④ The extrusion of soil between piles (double-row anti-slide piles)**

**Fig 29:**
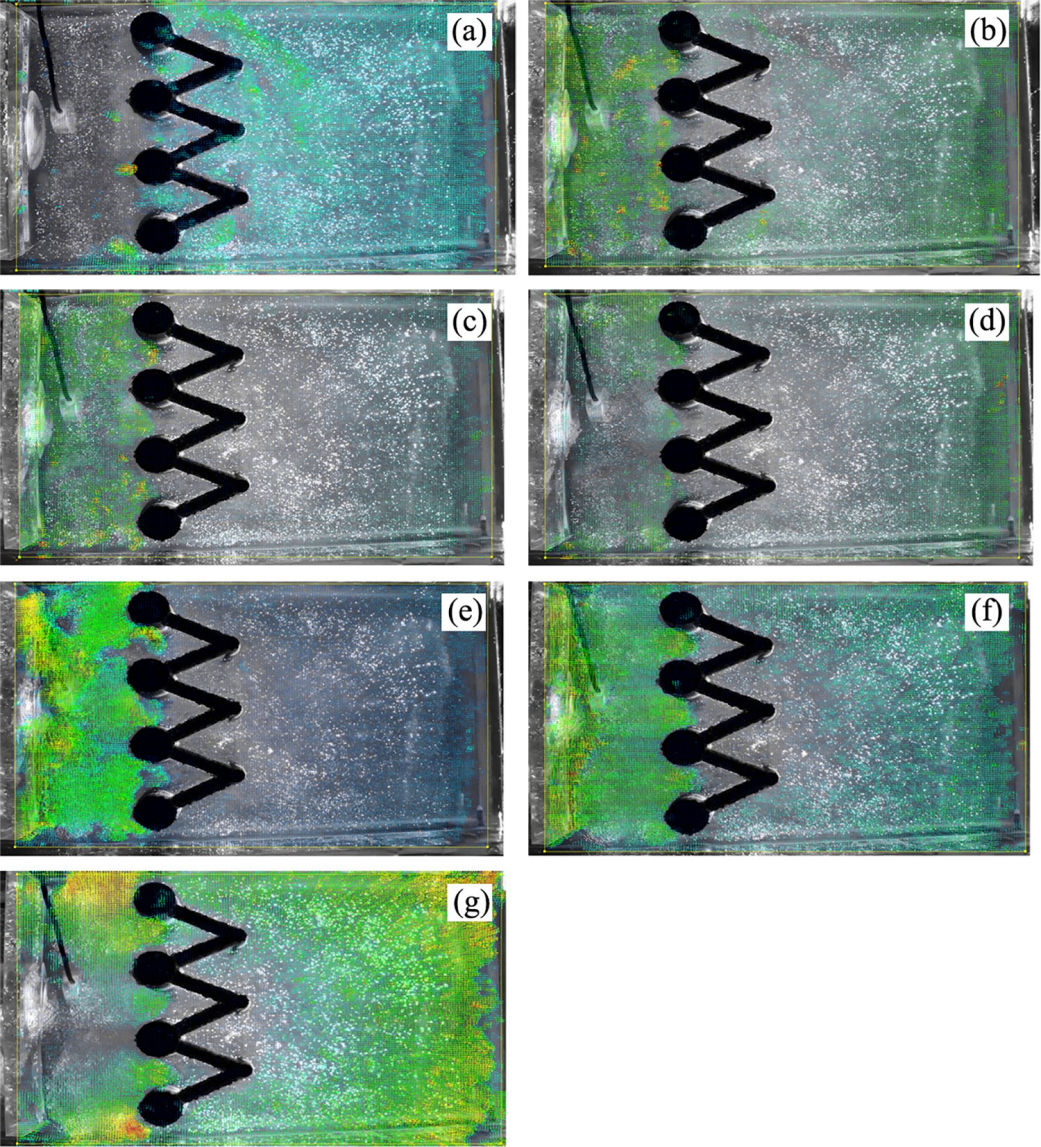
The extrusion of soil between double-row anti-slide pile. (The 7 figures are arrange according to the test time).

The extrusion of soil between the piles was identified (Fig 29). In Fig 29(A), the pile deforms in coordination with the soil under thrust pressure. There was some displacement in the area surrounding the anti-slide pile. Compared with Fig 29(A) and 29(B) shows how the loading plate moves forward, but the soil displacement is limited due to the double row anti-slide piles, and the soil experiences extrusion behind the back row piles, with a tendency to shear out. In Fig 29(C)–29(F), the loading plate has been moved further forward, resulting in obvious displacement of the soil between the piles and shearing out of the soil. However, the maximum shear area does not exceed the diameter of the back row piles. In Fig 29(G), the thrust pressure reaches its peak value, and the test ends. The soil displacement between piles no longer increases.

In summary, the process of surface displacement evolution between single-row piles and the soil is mostly coordinated deformation. This means that the pile and the soil deform together, and the thrust pressure increases to a certain extent, causing the soil to shear out. The range of shear displacement of the soil is larger. On the other hand, double-row piles have a better support effect, effectively blocking the soil. The range of shear displacement of the soil is much smaller than that of a single-row pile.

#### (4) Deformation analysis of the anti-slide piles

**Fig 30:**
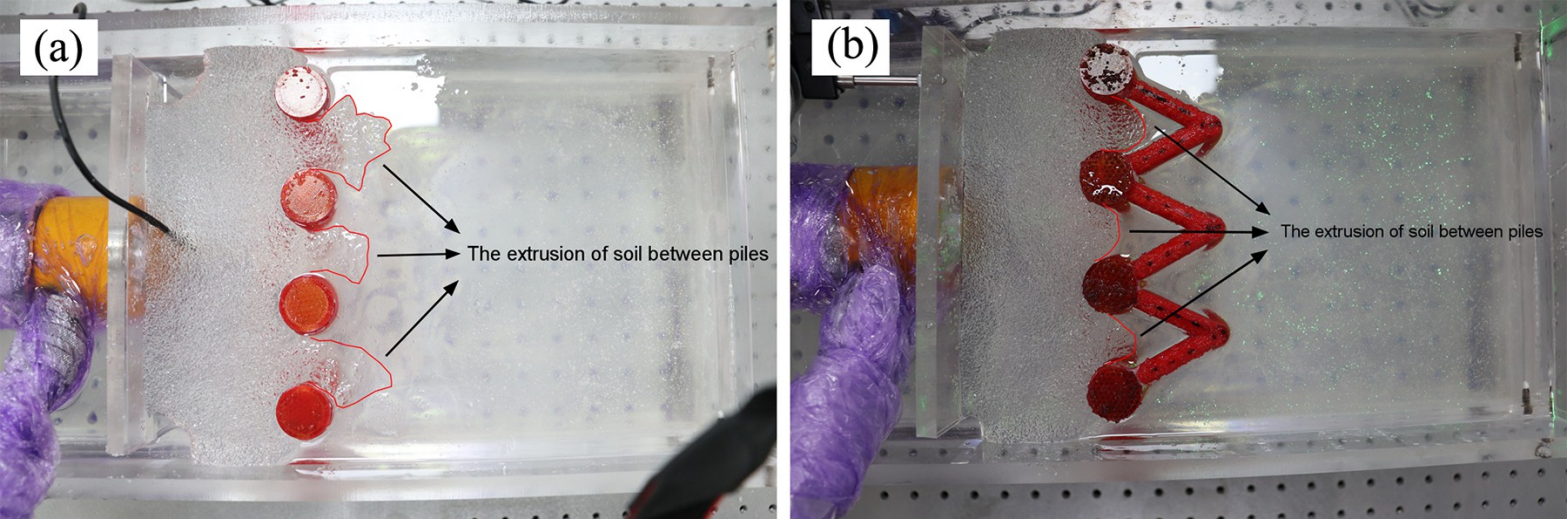
Test images from top view of anti-slide pile by camera: (a) single row anti-slide pile; (b) double row anti-slide pile.

Fig 30 shows a top view of the soil between the piles in the single-row pile and double-row pile tests. The soil underwent a shearing phenomenon under the influence of thrust pressure. Comparing the range of soil shearing out, that of the single-row piles is clearly larger than that of the double-row piles; this may be due to the front pile of the double-row piles having a certain supporting effect on the soil. The displacement of the front piles is smaller, suggesting that they may only bear a small portion of the thrust pressure. It is believed that the back row piles bear most of the pressure.

**Fig 31:**
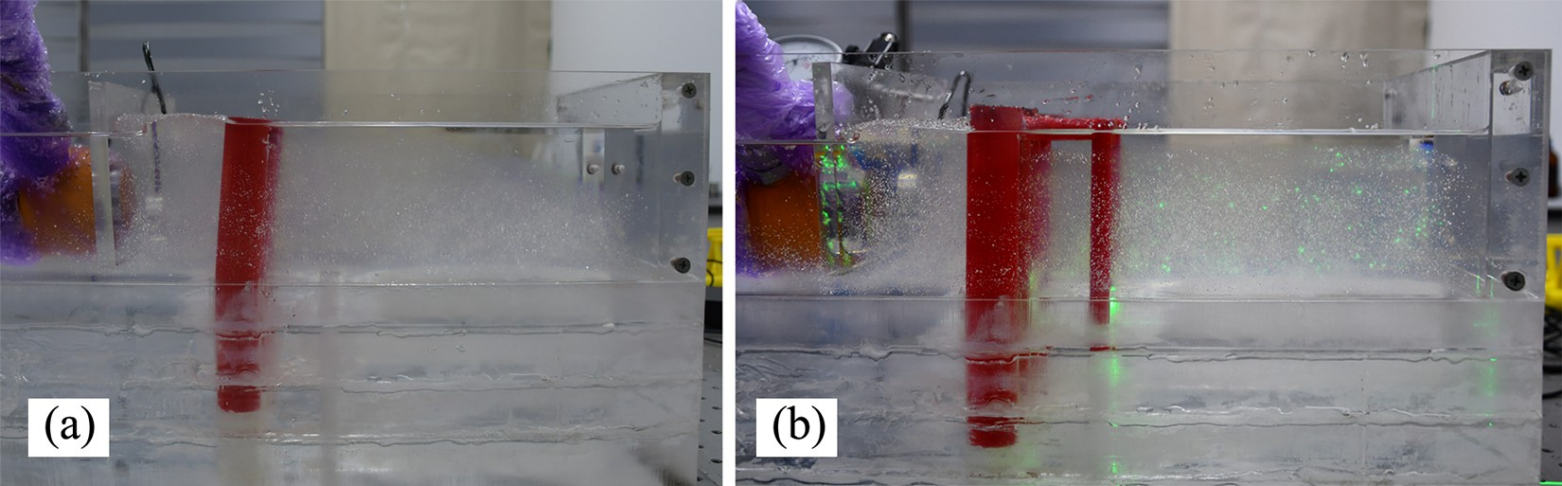
Test images from side view of anti-slide pile by camera (a) single row anti-slide pile: (b) double row anti-slide pile.

The side view of the anti-slip pile tests reveals the following findings, as shown in Fig 31: ① the soil behind the two types of piles is affected by the thrust pressure, resulting in an obvious bulge of approximately 1 cm in height, which indicates that both types of antislip piles are able to withstand the thrust pressure and are able to block the soil. ② From the perspective of pile displacement, single-row anti-slide piles exhibit more significant displacement than do double-row anti-slide piles. The free section of a single row of anti-slide piles is curved; however, the front and back piles of the two rows of anti-slide piles exhibit coordinated deformation.

## 7 Discussion

In summary, the soil deep and at the surface, the extrusion of soil between piles, and the deformation of anti-slide piles were analyzed. It was found that the double-row piles have a better supporting effect. The anti-slide mechanism of the double-row piles is analyzed below.

### (1) Existence of the “displacement triangle”

During the model test, a triangular area with no displacement was observed in the deep part of the soil behind the pile. This area is referred to as the “***displacement triangle***” area in this paper. The stress state of the soil in this area was affected by the combined effects of the thrust pressure, the self-weight of the transparent soil, and the anti-slide pile. The left side of this triangular area was subjected to thrust pressure, whereas the right side was subjected to resistance from the anti-slide piles. Despite this, the triangle remained in force equilibrium and did not experience any displacement due to the resistance of the anti-slip piles. As the thrust pressure continued to increase, shear slip occurred within the soil, resulting in the formation of a slip surface along the arc at the upper edge of this triangular area. This area may also be found behind the anti-slide piles in a physical landslide control project, and the presence of this area may cause changes in the stress state of the soil behind the pile. Further study of the area of the “***displacement triangle”*** is necessary.

**Fig 32:**
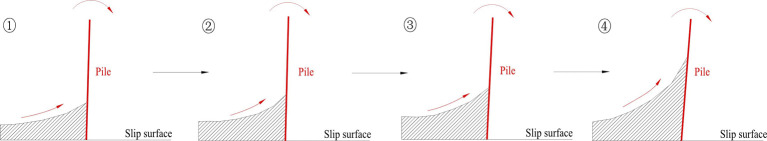
The area of “*displacement triangle*” of the soil behind the single row anti-slide pile.

The expansion of the "***displacement triangle***" behind a single-row pile occurs is shown in Fig 32. At the beginning of loading (Fig 32①), an area of a “***displacement triangle***” can be seen at the bottom of the soil behind the pile, the bottom of the triangle was affected by the boundary conditions, and the top of the triangle was likely an arc shape. As a result, the soil slipped along the arc, transferring the thrust pressure to the front anti-slide pile, which had a certain amount of offset toward the outside. In Fig 32② and ③, with increasing thrust pressure, owing to the support of the anti-slide pile, this triangular area expanded the arc upward, enlarging the triangular area. As shown in Fig 32④, the arc of the triangular area extended further upward. The top of the anti-slide pile experienced greater displacement because of the thrust pressure transferred by the soil. The soil sheared along the arc surface, and the soil behind the pile bulged. In this work, soil displacement between piles may only occur in the surface area.

**Fig 33:**
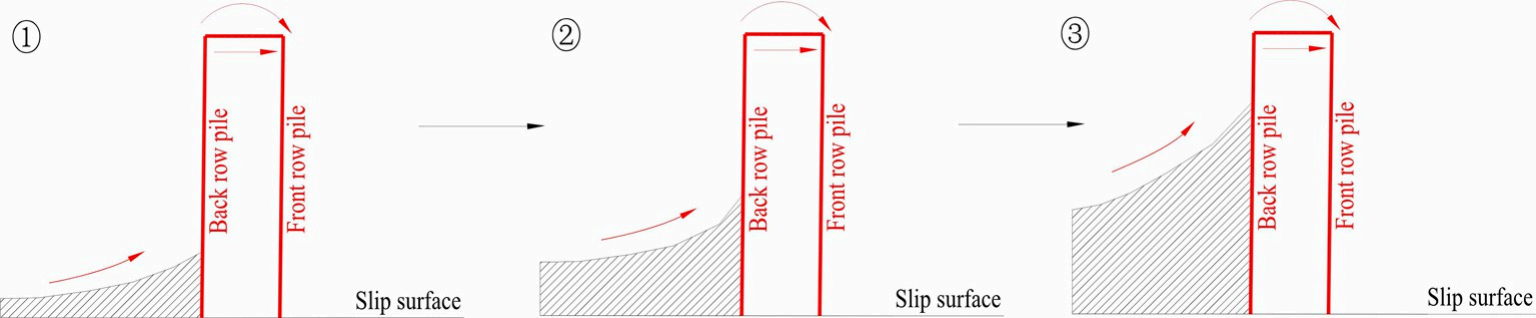
The area of “*displacement triangle*” of the soil behind the double row anti-slide pile.

As shown in Fig 33, the evolution rule of the “***displacement triangle***” of the double-row anti-slide pile differed from that of the single-row pile. The structure of the double-row pile offered greater resistance, resulting in a smaller tilt angle of the pile. With the continuous action of the thrust force, the area of the “***displacement triangle***” expanded more rapidly, causing the soil to slip along the upper edge of the triangle, and this arc had a larger angle, resulting in mainly vertical displacement of the soil behind the pile, which ultimately led to the uplift of the soil at the surface. However, the amount of soil shear displacement was smaller. As shown in Fig 33①, slight tilting of the anti-slide piles can be observed due to the thrust pressure, and a smaller area of “displacement triangle” formed in the bottom of the soil behind the back row pile along the slip surface. The double row of anti-slide piles provided more resistance; as a result, the upper arc of the displacement triangle extended upward along the pile (see Fig 33②). This area further expanded, and the soil slipped along the upper edge of this area, namely, the shear area gradually approached the surface, which resulted in stress being transferred to the top of the back row pile; then, the thrust pressure was transferred to the front row pile by the beam between the back and front piles (Fig 33③). In summary, despite single- and double-row pile model tests, the extrusion of soil between piles may occur only in the near-surface region [45].

Few studies have reported the phenomenon of a “***displacement triangle***”. In the previous study involving model test of anti-slide pile, the piles were subjected to bending deformation rather than fracture after the model test, with less displacement near the slip surface and more displacement away from them [45–47], which is consistent with the results of this study. However, this phenomenon of a “***displacement triangle***” has not yet been sufficiently identified because of the limitations of traditional physical model test methods, which are not effectively precise to identify the evolution of deformation in the soil. One of the most significant advantages of the method used in this study is its ability to identify deformation in the soil. In addition, time costs and expenses also constrain the repetition of model tests on anti-slide piles. Notably, when 3D printing technology is used, the 3D printed double-row anti-slide pile structure in this work has a material cost of only 200 RMB, a printing time of approximately 25 hours, and a preparation time of only 1 day between the two model tests.

**Fig 34:**
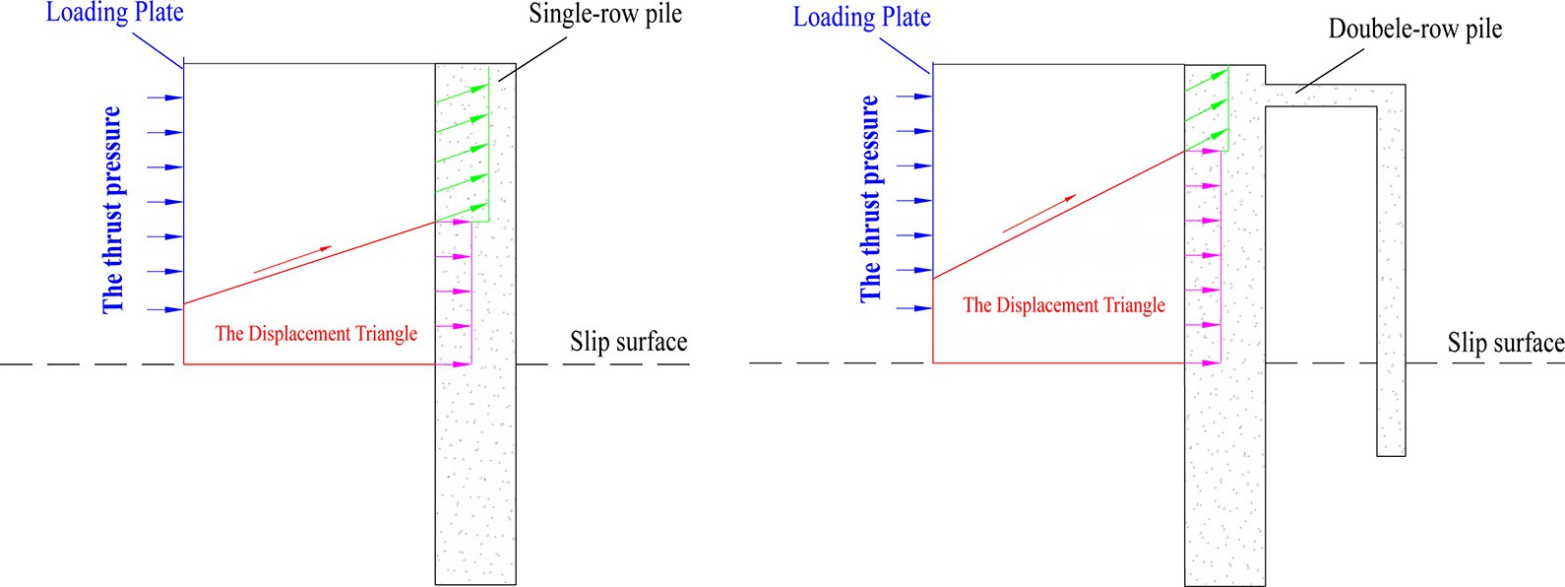
The direction of stress behind the pile.

According to the “Code for the Design of Landslide Stabilization” (GB/T 38509–2020) published by the Standardization Administration of China in March 6, 2020, the stress distributions of anti-slide piles are classified as trapezoidal, triangular, rectangular, etc [38]. Due to the influence of the *“****displacement triangle****”*, the stress distribution behind the pile may not be the case. As shown in Fig 34, the direction of stress behind the pile in the upper area of the *“****displacement triangle****”* may be diagonally upward, with a uniform load perpendicular to the pile in the lower area of the displacement triangle. Hence, this form of stress distribution may affect the design of anti-slide piles. From the test results in this paper, the difference between single-row and double-row piles lies in the size of the “***displacement triangle***”, with the double-row pile being subjected to a larger area of uniform loading. Many factors affect the evolution of the displacement triangle, such as row spacing and the presence of connecting beams. Excessive row spacing may affect the overall stiffness of a double-row pile and increase the displacement of the top of the pile [22,23].

### (2) Support effect on the pile displacement

**Fig 35:**
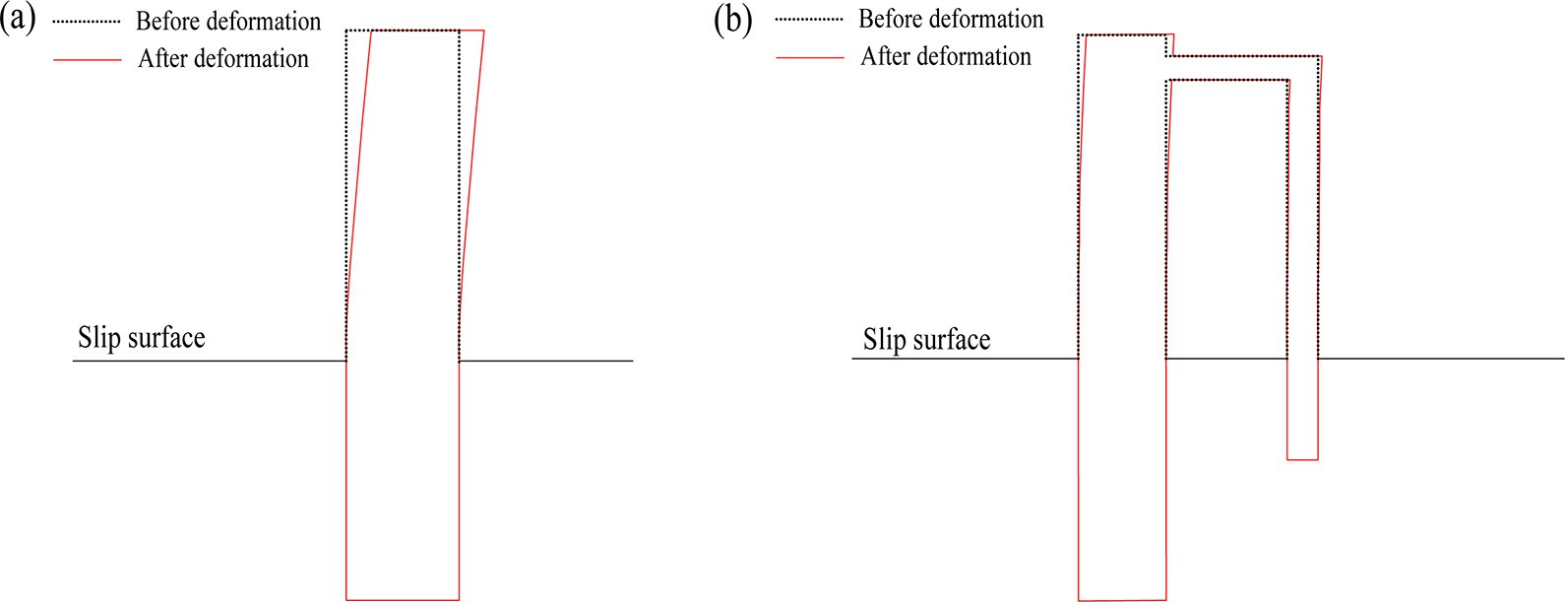
The displacement of pile before and after the test: (a) single row anti-slide pile; (b) double row anti-slide pile.

Fig 35 shows a comparison of the deformation for the two types of piles before and after the model tests. The dashed line represents the original position of the pile, whereas the red line represents the position after the test. As shown in Fig 35(A), the farther away from the sliding surface, the greater the deformation of the single-row pile, such that the displacement of the top of the pile was the largest, and the displacement close to the slip surface was the smallest. In Fig 35(B), the overall displacement for the double-row piles was much smaller than that of the single-row piles, and the front and back piles had a small amount of displacement, which reflected the supporting effect of the double-row piles.

In previous studies, the double row anti-slide pile, especially the h-type anti-slide piles, were proven to have a better supporting effect on landslide prevention and control projects. The size and shape of the front and back rows of h-type anti-slide piles are generally the same [40–43]. Unlike common h-type anti-slide piles, the anti-slide piles in this paper are a combination of large-diameter circular piles at the back row and small-diameter circular piles at the front row, and the test result show that they also have a good supporting effect.

### (3) The extrusion of soil between piles

**Fig 36:**
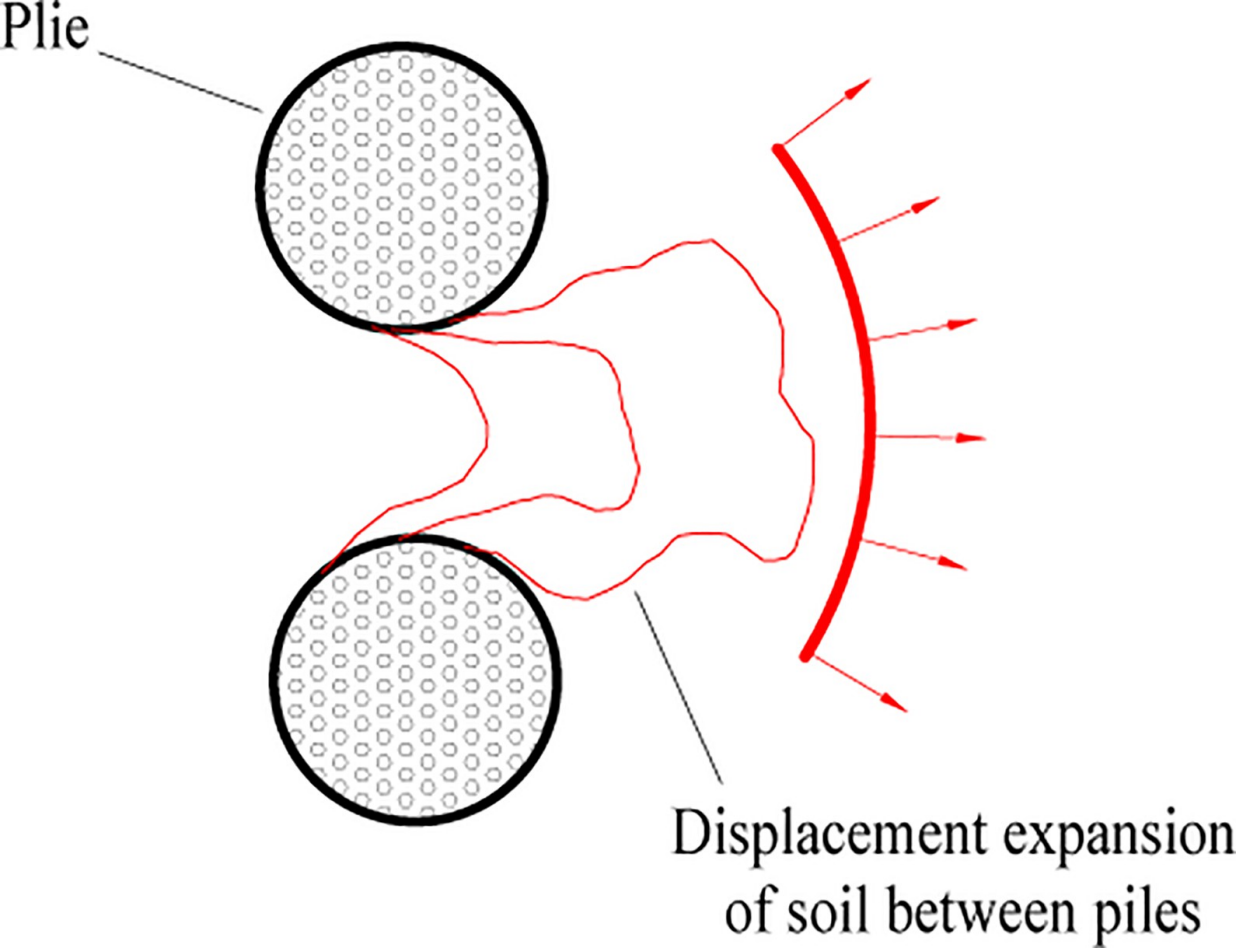
The schematic diagrams of the extrusion of soil between single row piles.

**Fig 37:**
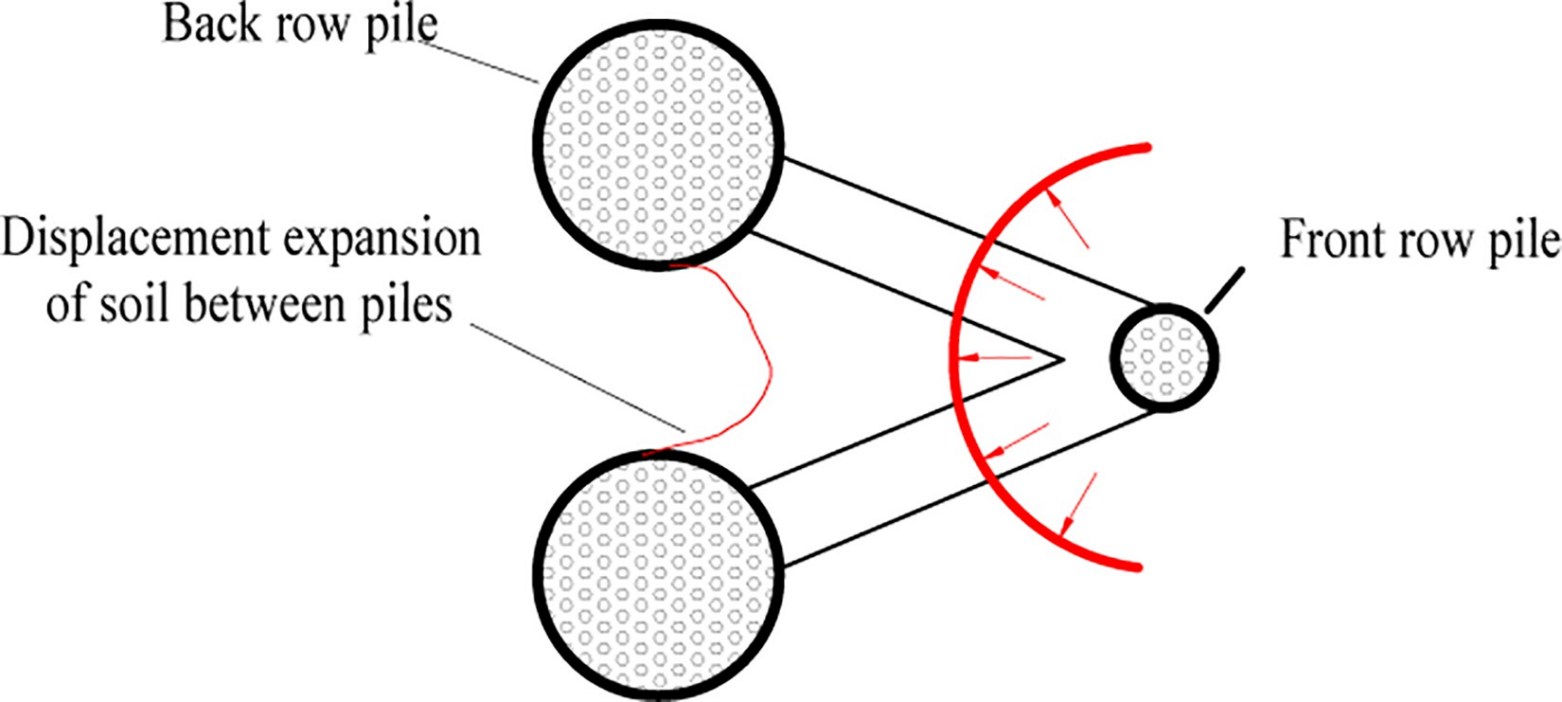
The schematic diagrams of the extrusion of soil between double row piles.

Schematic diagrams of soil shearing extension between single- and double-row piles were drawn to analyze the extrusion phenomenon of soil between piles (Figs 36 and 37).

The contour of the soil displacement between the single row piles took the shape of a fan that constantly expanded forward (see Fig 36). There is no resistance in front of the piles, and the largest displacement contour can reach 1–2 times the diameter of the pile. In the previous section, the continuous displacement expansion of the soil at the surface behind the pile was caused by the constant expansion of the deep soil. Therefore, the single row pile had a limited effect on controlling the displacement of the landslide.

For the double-row pile, the observed displacement expansion of the soil was distributed only between the back row piles (Fig 37), which may be attributed to two aspects: first, the deep displacement was controlled under the action of the two-row piles; and second, the front row piles had a certain suppression effect on the shear-out soil from the back row piles, where the soil between the front and back row piles was squeezed and the extrusion of the soil was stopped.

The phenomenon of soil shearing out between piles has been widely observed in model tests. In single-row pile model tests, the soil behind piles can cross the tops of piles to produce significant deformation [45,46]. Nevertheless, the shear-out phenomenon of soil between piles in two rows of piles has not yet been sufficiently investigated, and further studies are needed.

In summary, the test method in this paper combines transparent soil, 3D printing, and PIV technologies, which can be carried out quickly and repeatedly and can be enough to effectively identify deformation inside the soil behind single-row and double-row anti-slide piles, with a lower time cost and expense.

## 8 Conclusion

Three aspects were investigated based on the model tests of single-row and double-row anti-slide piles, with the following conclusions:

1. A new model test method was developed.
  The advantages and disadvantages of the traditional model test method for anti-slide piles were analyzed, and a new model test method was proposed. The method used in this study combines a PIV system, transparent soil technology, and 3D printing technology. It offers several advantages over traditional test methods, including time savings, data accuracy, and convenient data collection.
2. A series of model tests of single- and double-row anti-slide piles were carried out.
  Using the new model test system, single- and double-row anti-slide pile simulation tests were carried out to analyze the internal displacement of the soil and the evolution of the soil surface displacement after the pile and to verify the applicability of this model test method.
3. The anti-slide mechanism was studied.
  The displacement evolution of deep and surface soil, deformation analysis of anti-slide piles, and extrusion of soil between piles were analyzed, and the phenomenon of the “displacement triangle” area was identified. The double-row anti-slide piles can effectively control the deep displacement of the soil behind the piles. Under the combined influence of the front and back row piles, the displacement of the top of the piles can also be effectively controlled. The front row piles have a controlling effect on the shearing out of the soil between the back piles.

## Supporting information

S1 File Data of axial force and displacement of square infill pattern. (XLS)

S1 Appendix Test images from side view of single row anti-slide pile by MicroVec (20 images). **Appendix Ⅰ S1** 1st test image. **Appendix Ⅰ S2** 2nd test image. **Appendix Ⅰ S3** 3rd test image. **Appendix Ⅰ S4** 4th test image. **Appendix Ⅰ S5** 5th test image. **Appendix Ⅰ S6** 6th test image. **Appendix Ⅰ S7** 7th test image. **Appendix Ⅰ S8** 8th test image. **Appendix Ⅰ S9** 9th test image. **Appendix Ⅰ S10** 10th test image. **Appendix Ⅰ S11** 11th test image. **Appendix Ⅰ S12** 12th test image. **Appendix Ⅰ S13** 13th test image. **Appendix Ⅰ S14** 14th test image. **Appendix Ⅰ S15** 15th test image. **Appendix Ⅰ S16** 16th test image. **Appendix Ⅰ S17 1**7th test image. **Appendix Ⅰ S18 1**8th test image. **Appendix Ⅰ S19 1**9th test image. **Appendix Ⅰ S20 20**th test image.
(ZIP)

S2 Appendix Test images from side view of double row anti-slide pile by MicroVec (5 images). **Appendix Ⅱ S1** 1st test image. **Appendix Ⅱ S2** 2nd test image. **Appendix Ⅱ S3** 3rd test image. **Appendix Ⅱ S4** 4th test image. **Appendix Ⅱ S5** 5th test image.
(ZIP)
