# Supplementary figures and images for: Study on the anti-slide mechanism of double-row circular pile by model test using PIV, transparent soil material and 3D printing technology
Source: PLoS One. 2024 Sep 25;19(9):e0309727. doi: 10.1371/journal.pone.0309727 (PMC11423988; doi:10.1371/journal.pone.0309727)

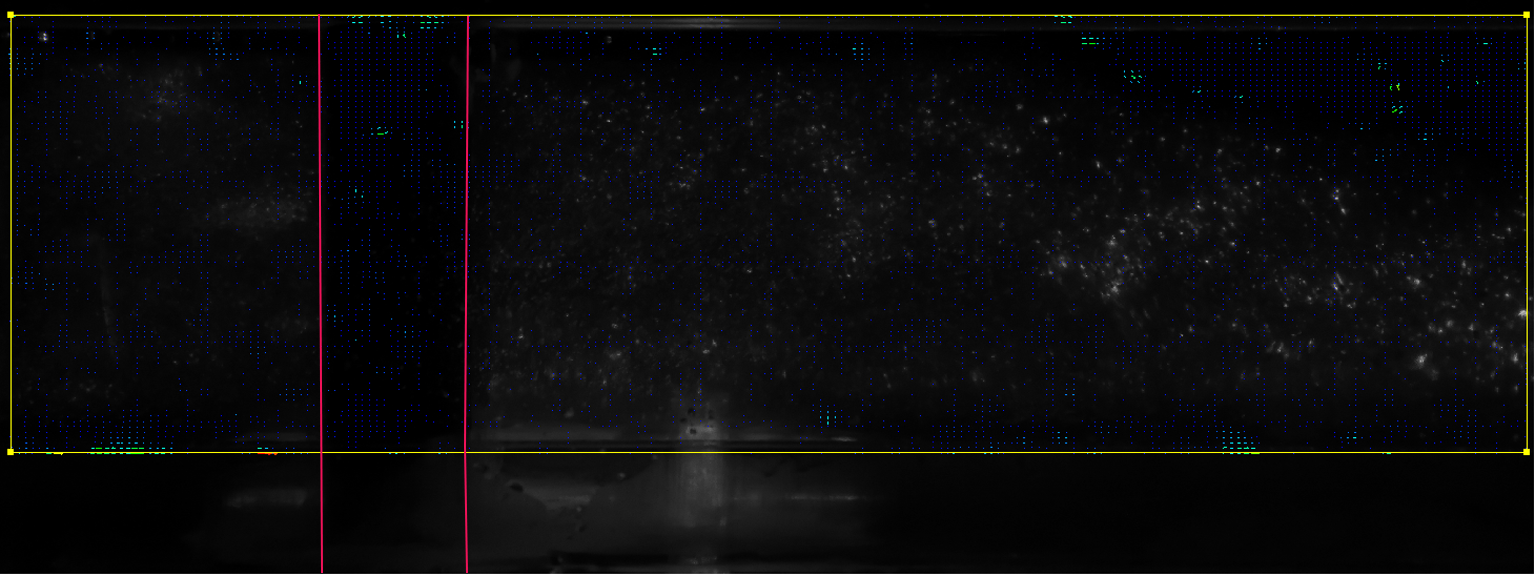

Supplement: S1 Appendix — Appendix Ⅰ S1 1st test image. Appendix Ⅰ S2 2nd test image. Appendix Ⅰ S3 3rd test image. Appendix Ⅰ S4 4th test image. Appendix Ⅰ S5 5th test image. Appendix Ⅰ S6 6th test image. Appendix Ⅰ S7 7th test image. Appendix Ⅰ S8 8th test image. Appendix Ⅰ S9 9th test image. Appendix Ⅰ S10 10th test image. Appendix Ⅰ S11 11th test image. Appendix Ⅰ S12 12th test image. Appendix Ⅰ S13 13th test image. Appendix Ⅰ S14 14th test image. Appendix Ⅰ S15 15th test image. Appendix Ⅰ S16 16th test image. Appendix Ⅰ S17 17th test image. Appendix Ⅰ S18 18th test image. Appendix Ⅰ S19 19th test image. Appendix Ⅰ S20 20th test image. (ZIP) [file pone.0309727.s002.zip › Appendix I S1.tif]

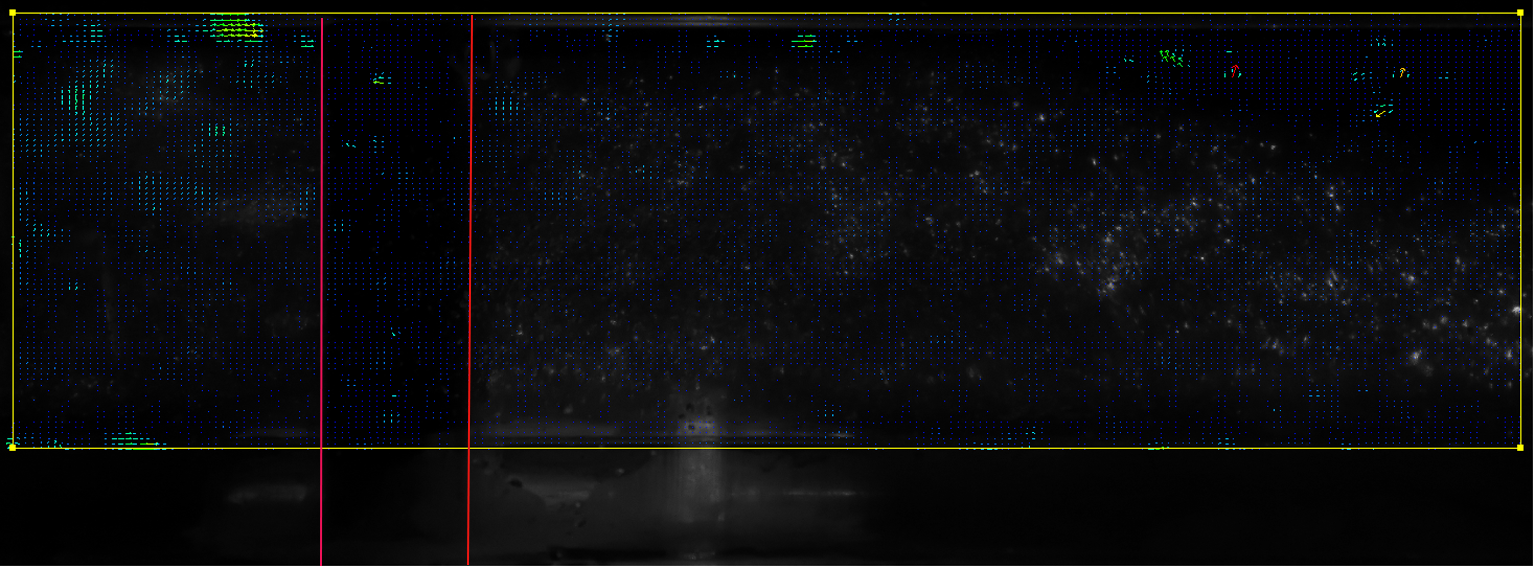

Supplement: S1 Appendix — Appendix Ⅰ S1 1st test image. Appendix Ⅰ S2 2nd test image. Appendix Ⅰ S3 3rd test image. Appendix Ⅰ S4 4th test image. Appendix Ⅰ S5 5th test image. Appendix Ⅰ S6 6th test image. Appendix Ⅰ S7 7th test image. Appendix Ⅰ S8 8th test image. Appendix Ⅰ S9 9th test image. Appendix Ⅰ S10 10th test image. Appendix Ⅰ S11 11th test image. Appendix Ⅰ S12 12th test image. Appendix Ⅰ S13 13th test image. Appendix Ⅰ S14 14th test image. Appendix Ⅰ S15 15th test image. Appendix Ⅰ S16 16th test image. Appendix Ⅰ S17 17th test image. Appendix Ⅰ S18 18th test image. Appendix Ⅰ S19 19th test image. Appendix Ⅰ S20 20th test image. (ZIP) [file pone.0309727.s002.zip › Appendix I S2.tif]

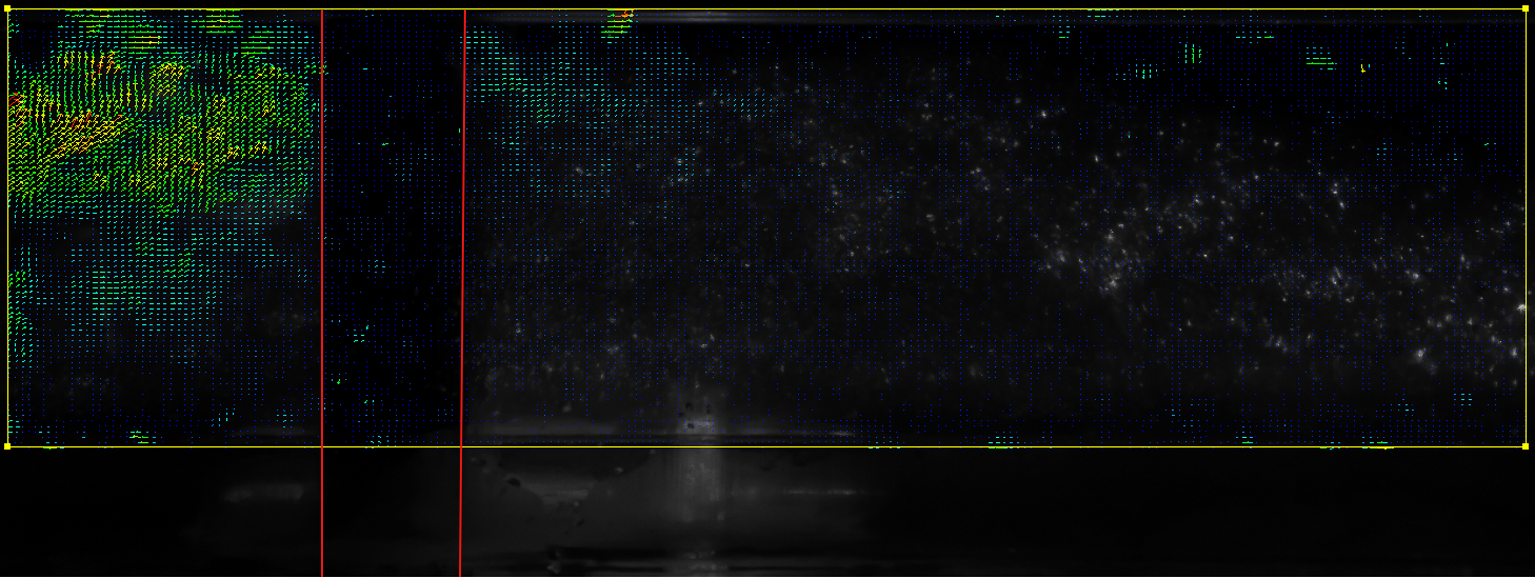

Supplement: S1 Appendix — Appendix Ⅰ S1 1st test image. Appendix Ⅰ S2 2nd test image. Appendix Ⅰ S3 3rd test image. Appendix Ⅰ S4 4th test image. Appendix Ⅰ S5 5th test image. Appendix Ⅰ S6 6th test image. Appendix Ⅰ S7 7th test image. Appendix Ⅰ S8 8th test image. Appendix Ⅰ S9 9th test image. Appendix Ⅰ S10 10th test image. Appendix Ⅰ S11 11th test image. Appendix Ⅰ S12 12th test image. Appendix Ⅰ S13 13th test image. Appendix Ⅰ S14 14th test image. Appendix Ⅰ S15 15th test image. Appendix Ⅰ S16 16th test image. Appendix Ⅰ S17 17th test image. Appendix Ⅰ S18 18th test image. Appendix Ⅰ S19 19th test image. Appendix Ⅰ S20 20th test image. (ZIP) [file pone.0309727.s002.zip › Appendix I S3.tif]

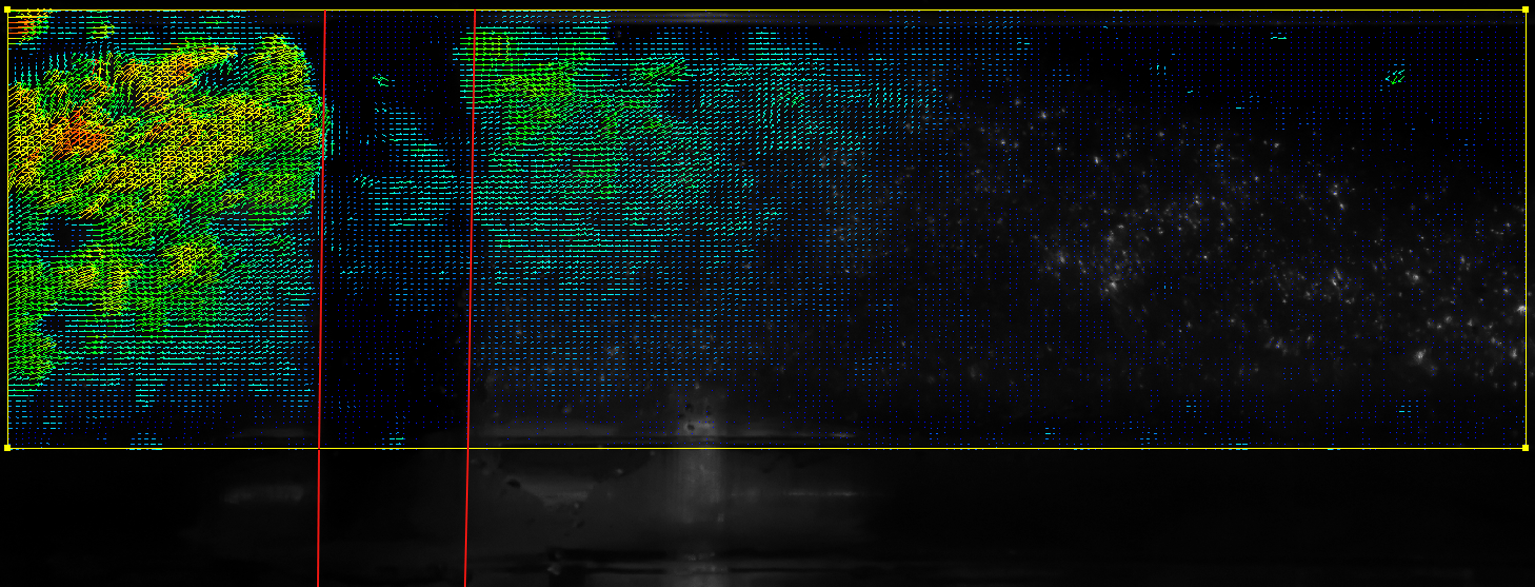

Supplement: S1 Appendix — Appendix Ⅰ S1 1st test image. Appendix Ⅰ S2 2nd test image. Appendix Ⅰ S3 3rd test image. Appendix Ⅰ S4 4th test image. Appendix Ⅰ S5 5th test image. Appendix Ⅰ S6 6th test image. Appendix Ⅰ S7 7th test image. Appendix Ⅰ S8 8th test image. Appendix Ⅰ S9 9th test image. Appendix Ⅰ S10 10th test image. Appendix Ⅰ S11 11th test image. Appendix Ⅰ S12 12th test image. Appendix Ⅰ S13 13th test image. Appendix Ⅰ S14 14th test image. Appendix Ⅰ S15 15th test image. Appendix Ⅰ S16 16th test image. Appendix Ⅰ S17 17th test image. Appendix Ⅰ S18 18th test image. Appendix Ⅰ S19 19th test image. Appendix Ⅰ S20 20th test image. (ZIP) [file pone.0309727.s002.zip › Appendix I S4.tif]

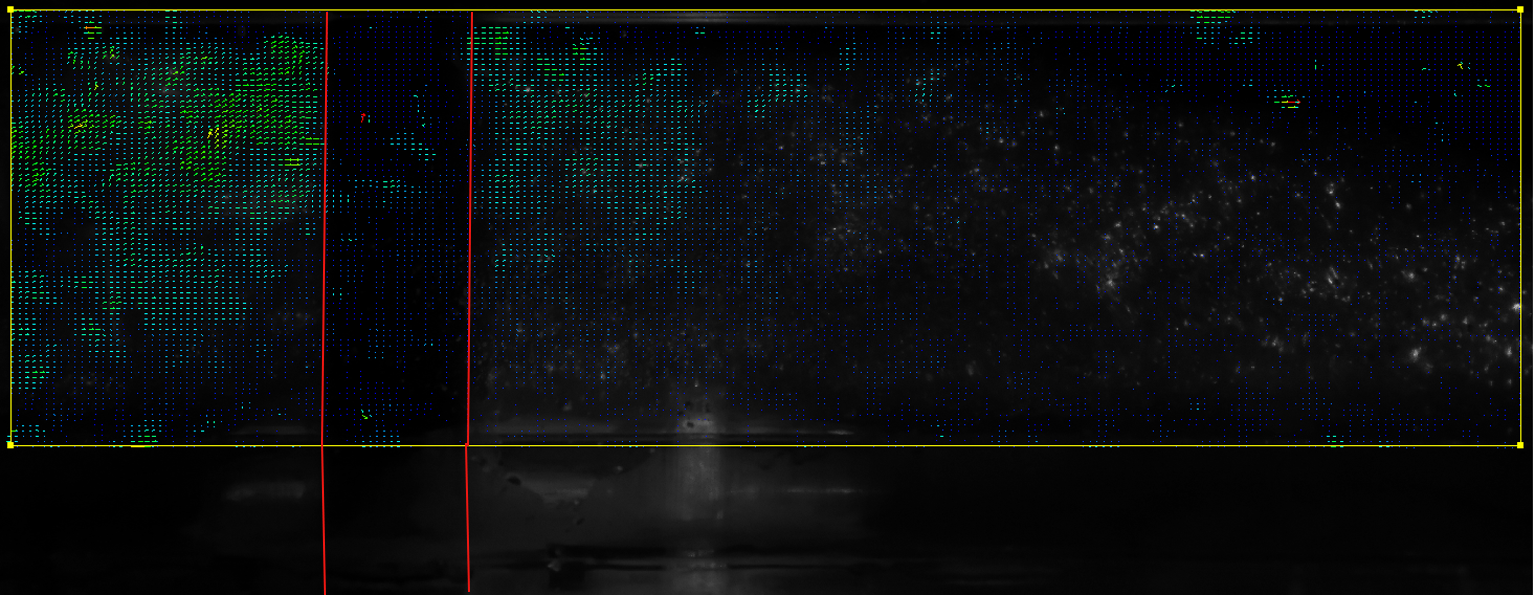

Supplement: S1 Appendix — Appendix Ⅰ S1 1st test image. Appendix Ⅰ S2 2nd test image. Appendix Ⅰ S3 3rd test image. Appendix Ⅰ S4 4th test image. Appendix Ⅰ S5 5th test image. Appendix Ⅰ S6 6th test image. Appendix Ⅰ S7 7th test image. Appendix Ⅰ S8 8th test image. Appendix Ⅰ S9 9th test image. Appendix Ⅰ S10 10th test image. Appendix Ⅰ S11 11th test image. Appendix Ⅰ S12 12th test image. Appendix Ⅰ S13 13th test image. Appendix Ⅰ S14 14th test image. Appendix Ⅰ S15 15th test image. Appendix Ⅰ S16 16th test image. Appendix Ⅰ S17 17th test image. Appendix Ⅰ S18 18th test image. Appendix Ⅰ S19 19th test image. Appendix Ⅰ S20 20th test image. (ZIP) [file pone.0309727.s002.zip › Appendix I S5.tif]

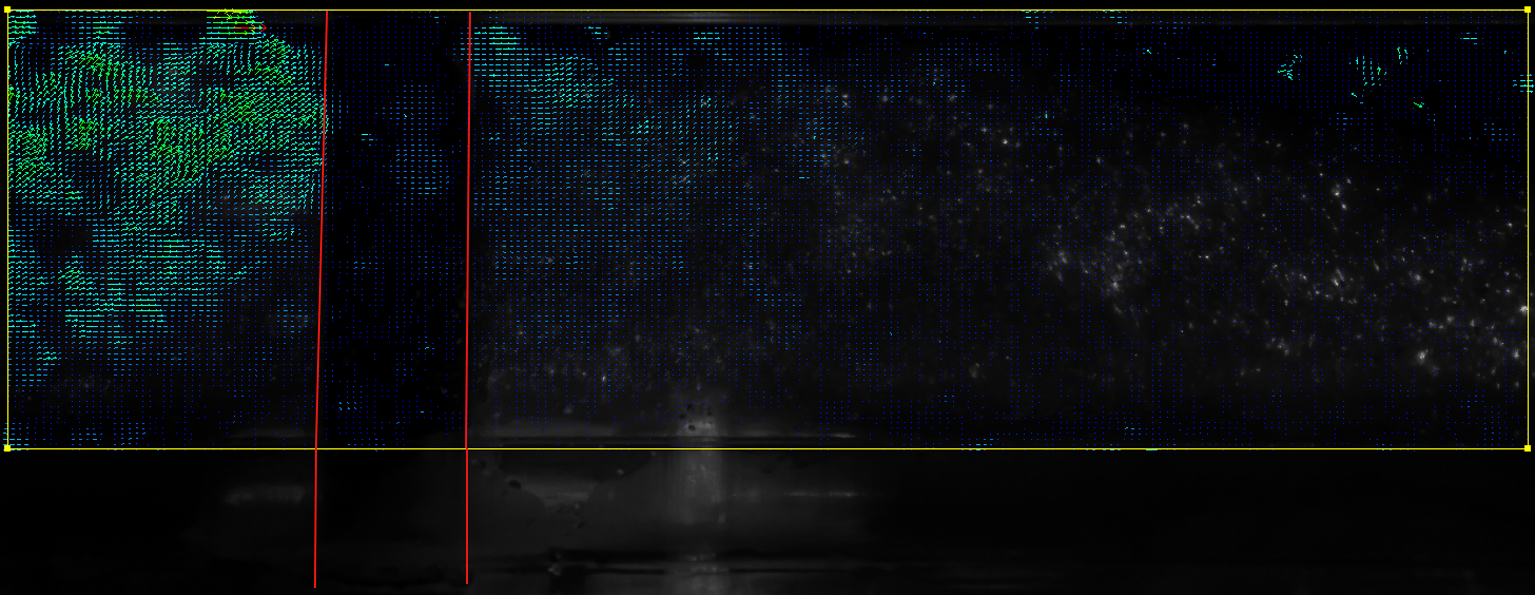

Supplement: S1 Appendix — Appendix Ⅰ S1 1st test image. Appendix Ⅰ S2 2nd test image. Appendix Ⅰ S3 3rd test image. Appendix Ⅰ S4 4th test image. Appendix Ⅰ S5 5th test image. Appendix Ⅰ S6 6th test image. Appendix Ⅰ S7 7th test image. Appendix Ⅰ S8 8th test image. Appendix Ⅰ S9 9th test image. Appendix Ⅰ S10 10th test image. Appendix Ⅰ S11 11th test image. Appendix Ⅰ S12 12th test image. Appendix Ⅰ S13 13th test image. Appendix Ⅰ S14 14th test image. Appendix Ⅰ S15 15th test image. Appendix Ⅰ S16 16th test image. Appendix Ⅰ S17 17th test image. Appendix Ⅰ S18 18th test image. Appendix Ⅰ S19 19th test image. Appendix Ⅰ S20 20th test image. (ZIP) [file pone.0309727.s002.zip › Appendix I S6.tif]

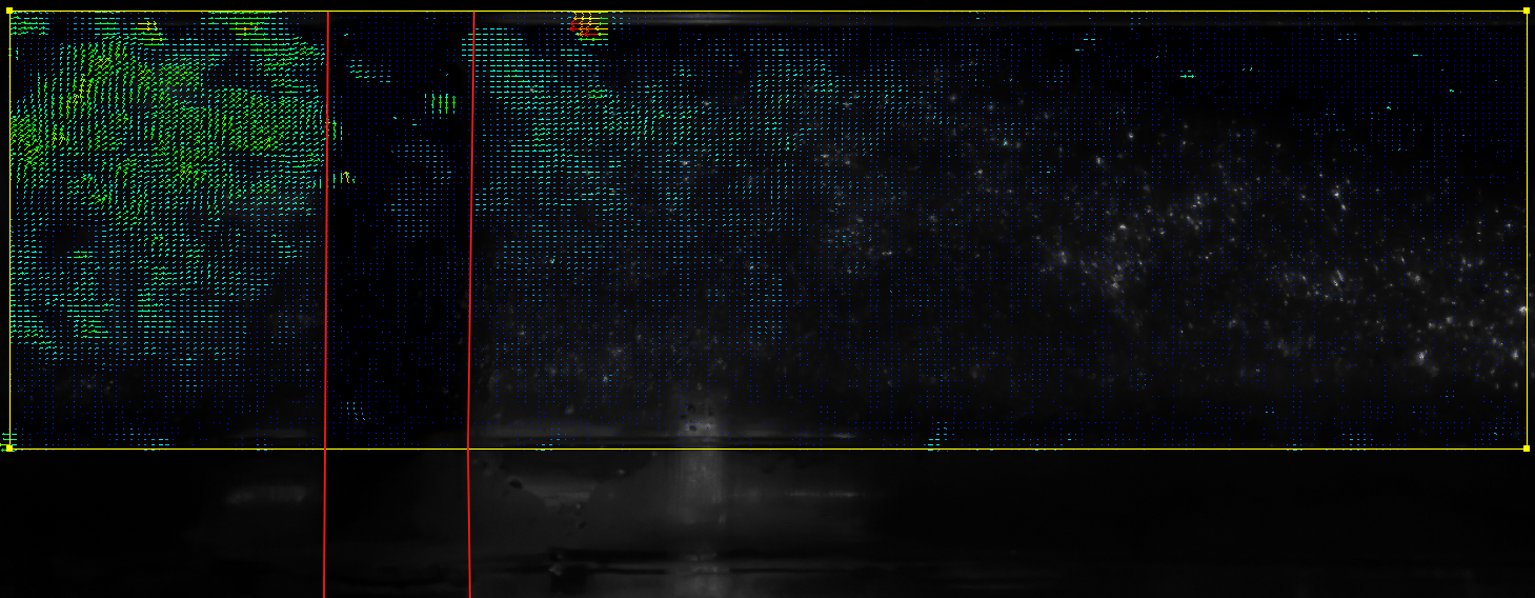

Supplement: S1 Appendix — Appendix Ⅰ S1 1st test image. Appendix Ⅰ S2 2nd test image. Appendix Ⅰ S3 3rd test image. Appendix Ⅰ S4 4th test image. Appendix Ⅰ S5 5th test image. Appendix Ⅰ S6 6th test image. Appendix Ⅰ S7 7th test image. Appendix Ⅰ S8 8th test image. Appendix Ⅰ S9 9th test image. Appendix Ⅰ S10 10th test image. Appendix Ⅰ S11 11th test image. Appendix Ⅰ S12 12th test image. Appendix Ⅰ S13 13th test image. Appendix Ⅰ S14 14th test image. Appendix Ⅰ S15 15th test image. Appendix Ⅰ S16 16th test image. Appendix Ⅰ S17 17th test image. Appendix Ⅰ S18 18th test image. Appendix Ⅰ S19 19th test image. Appendix Ⅰ S20 20th test image. (ZIP) [file pone.0309727.s002.zip › Appendix I S7.tif]

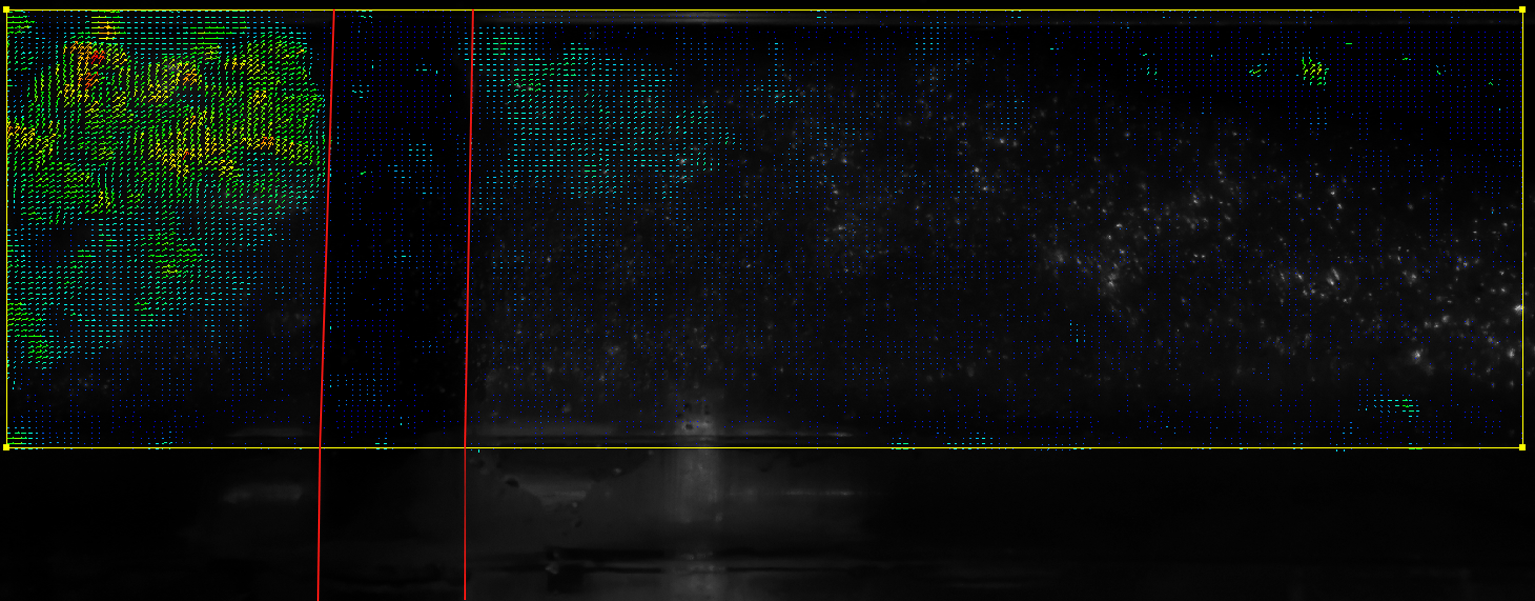

Supplement: S1 Appendix — Appendix Ⅰ S1 1st test image. Appendix Ⅰ S2 2nd test image. Appendix Ⅰ S3 3rd test image. Appendix Ⅰ S4 4th test image. Appendix Ⅰ S5 5th test image. Appendix Ⅰ S6 6th test image. Appendix Ⅰ S7 7th test image. Appendix Ⅰ S8 8th test image. Appendix Ⅰ S9 9th test image. Appendix Ⅰ S10 10th test image. Appendix Ⅰ S11 11th test image. Appendix Ⅰ S12 12th test image. Appendix Ⅰ S13 13th test image. Appendix Ⅰ S14 14th test image. Appendix Ⅰ S15 15th test image. Appendix Ⅰ S16 16th test image. Appendix Ⅰ S17 17th test image. Appendix Ⅰ S18 18th test image. Appendix Ⅰ S19 19th test image. Appendix Ⅰ S20 20th test image. (ZIP) [file pone.0309727.s002.zip › Appendix I S8.tif]

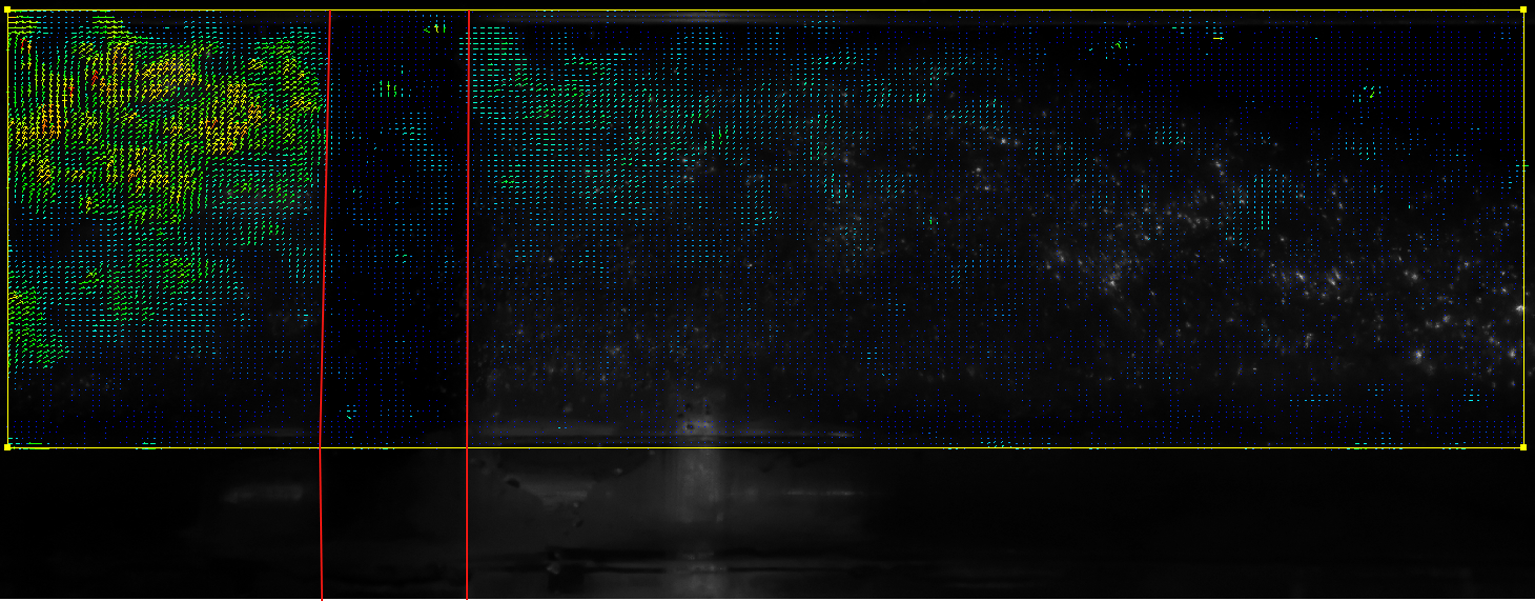

Supplement: S1 Appendix — Appendix Ⅰ S1 1st test image. Appendix Ⅰ S2 2nd test image. Appendix Ⅰ S3 3rd test image. Appendix Ⅰ S4 4th test image. Appendix Ⅰ S5 5th test image. Appendix Ⅰ S6 6th test image. Appendix Ⅰ S7 7th test image. Appendix Ⅰ S8 8th test image. Appendix Ⅰ S9 9th test image. Appendix Ⅰ S10 10th test image. Appendix Ⅰ S11 11th test image. Appendix Ⅰ S12 12th test image. Appendix Ⅰ S13 13th test image. Appendix Ⅰ S14 14th test image. Appendix Ⅰ S15 15th test image. Appendix Ⅰ S16 16th test image. Appendix Ⅰ S17 17th test image. Appendix Ⅰ S18 18th test image. Appendix Ⅰ S19 19th test image. Appendix Ⅰ S20 20th test image. (ZIP) [file pone.0309727.s002.zip › Appendix I S9.tif]

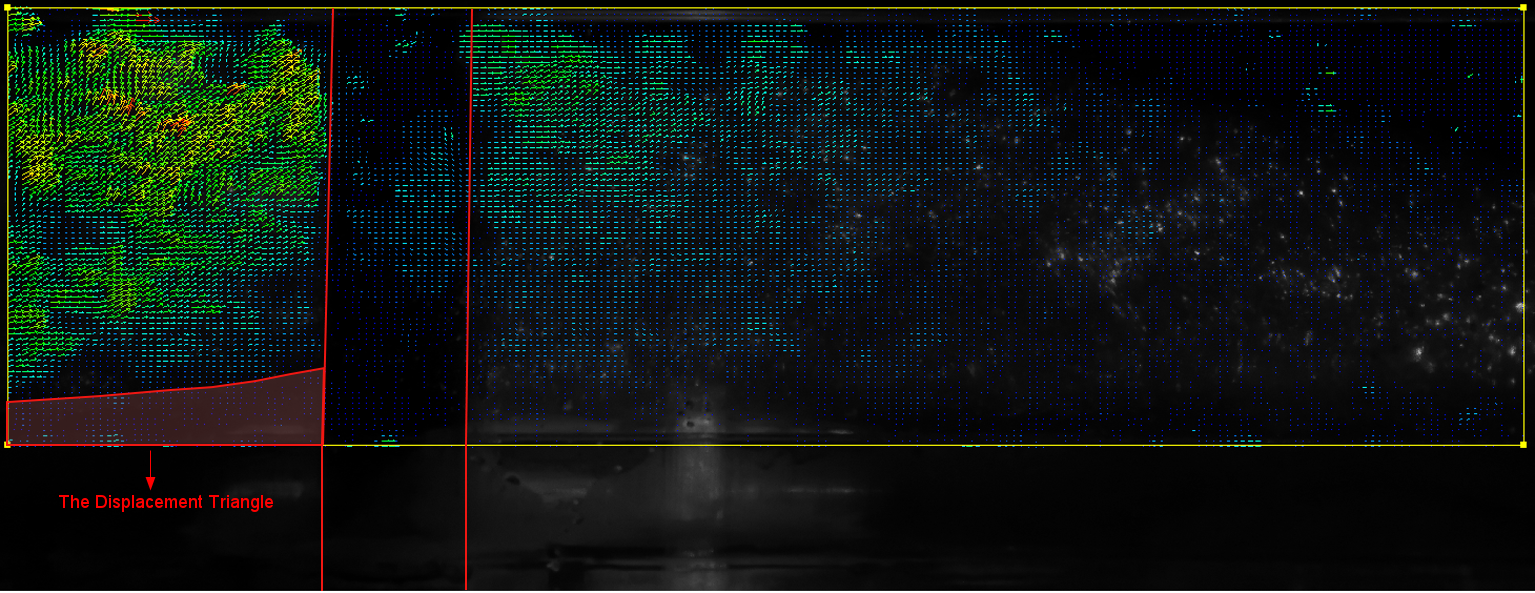

Supplement: S1 Appendix — Appendix Ⅰ S1 1st test image. Appendix Ⅰ S2 2nd test image. Appendix Ⅰ S3 3rd test image. Appendix Ⅰ S4 4th test image. Appendix Ⅰ S5 5th test image. Appendix Ⅰ S6 6th test image. Appendix Ⅰ S7 7th test image. Appendix Ⅰ S8 8th test image. Appendix Ⅰ S9 9th test image. Appendix Ⅰ S10 10th test image. Appendix Ⅰ S11 11th test image. Appendix Ⅰ S12 12th test image. Appendix Ⅰ S13 13th test image. Appendix Ⅰ S14 14th test image. Appendix Ⅰ S15 15th test image. Appendix Ⅰ S16 16th test image. Appendix Ⅰ S17 17th test image. Appendix Ⅰ S18 18th test image. Appendix Ⅰ S19 19th test image. Appendix Ⅰ S20 20th test image. (ZIP) [file pone.0309727.s002.zip › Appendix I S10.tif]

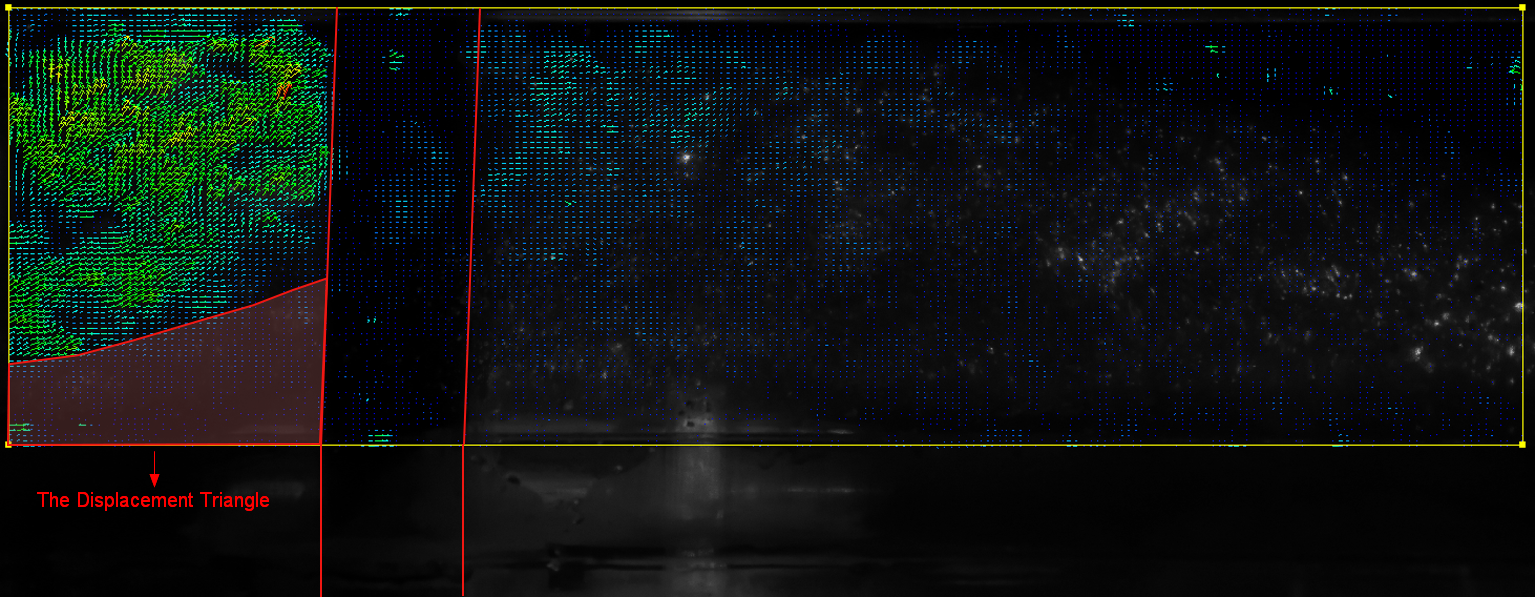

Supplement: S1 Appendix — Appendix Ⅰ S1 1st test image. Appendix Ⅰ S2 2nd test image. Appendix Ⅰ S3 3rd test image. Appendix Ⅰ S4 4th test image. Appendix Ⅰ S5 5th test image. Appendix Ⅰ S6 6th test image. Appendix Ⅰ S7 7th test image. Appendix Ⅰ S8 8th test image. Appendix Ⅰ S9 9th test image. Appendix Ⅰ S10 10th test image. Appendix Ⅰ S11 11th test image. Appendix Ⅰ S12 12th test image. Appendix Ⅰ S13 13th test image. Appendix Ⅰ S14 14th test image. Appendix Ⅰ S15 15th test image. Appendix Ⅰ S16 16th test image. Appendix Ⅰ S17 17th test image. Appendix Ⅰ S18 18th test image. Appendix Ⅰ S19 19th test image. Appendix Ⅰ S20 20th test image. (ZIP) [file pone.0309727.s002.zip › Appendix I S11.tif]

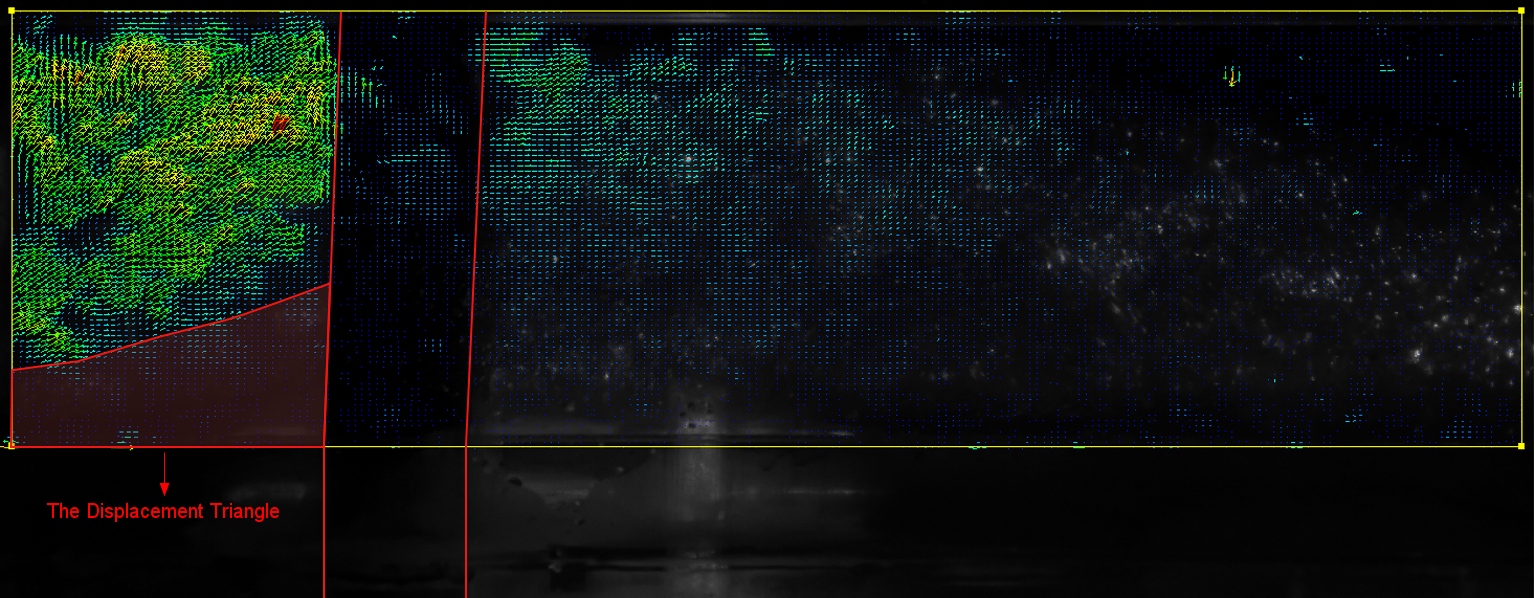

Supplement: S1 Appendix — Appendix Ⅰ S1 1st test image. Appendix Ⅰ S2 2nd test image. Appendix Ⅰ S3 3rd test image. Appendix Ⅰ S4 4th test image. Appendix Ⅰ S5 5th test image. Appendix Ⅰ S6 6th test image. Appendix Ⅰ S7 7th test image. Appendix Ⅰ S8 8th test image. Appendix Ⅰ S9 9th test image. Appendix Ⅰ S10 10th test image. Appendix Ⅰ S11 11th test image. Appendix Ⅰ S12 12th test image. Appendix Ⅰ S13 13th test image. Appendix Ⅰ S14 14th test image. Appendix Ⅰ S15 15th test image. Appendix Ⅰ S16 16th test image. Appendix Ⅰ S17 17th test image. Appendix Ⅰ S18 18th test image. Appendix Ⅰ S19 19th test image. Appendix Ⅰ S20 20th test image. (ZIP) [file pone.0309727.s002.zip › Appendix I S12.tif]

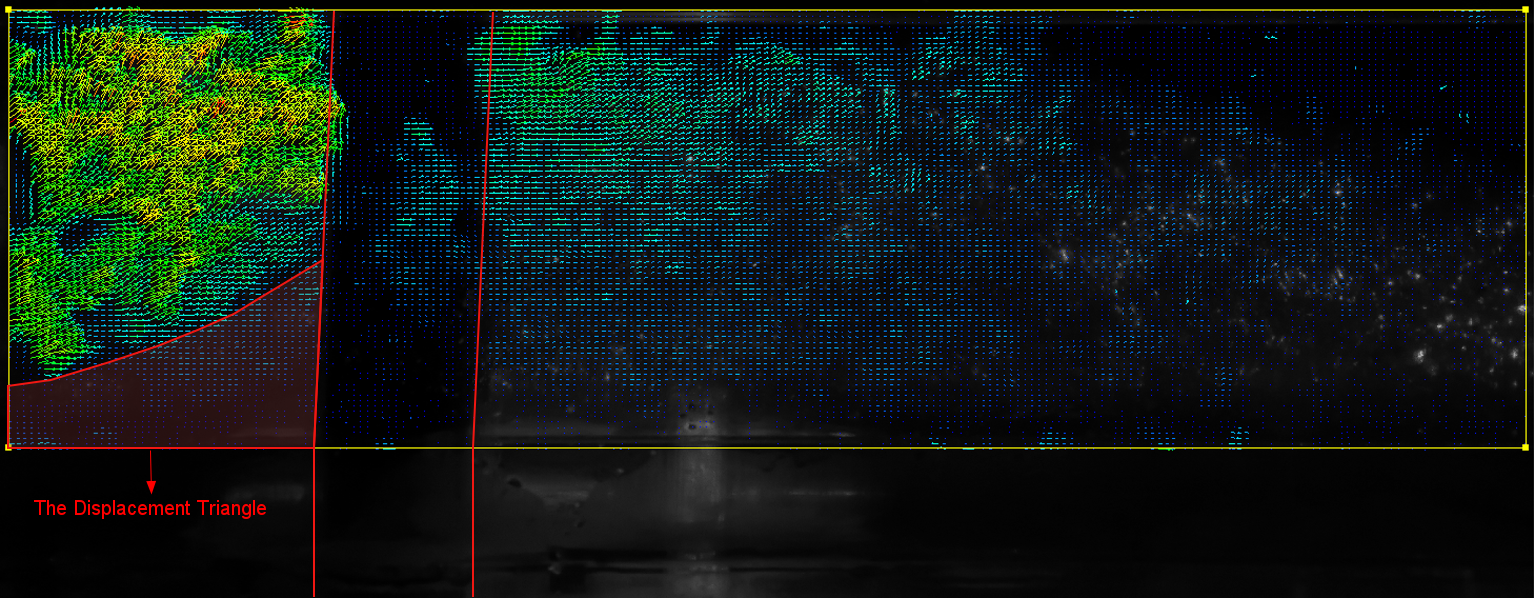

Supplement: S1 Appendix — Appendix Ⅰ S1 1st test image. Appendix Ⅰ S2 2nd test image. Appendix Ⅰ S3 3rd test image. Appendix Ⅰ S4 4th test image. Appendix Ⅰ S5 5th test image. Appendix Ⅰ S6 6th test image. Appendix Ⅰ S7 7th test image. Appendix Ⅰ S8 8th test image. Appendix Ⅰ S9 9th test image. Appendix Ⅰ S10 10th test image. Appendix Ⅰ S11 11th test image. Appendix Ⅰ S12 12th test image. Appendix Ⅰ S13 13th test image. Appendix Ⅰ S14 14th test image. Appendix Ⅰ S15 15th test image. Appendix Ⅰ S16 16th test image. Appendix Ⅰ S17 17th test image. Appendix Ⅰ S18 18th test image. Appendix Ⅰ S19 19th test image. Appendix Ⅰ S20 20th test image. (ZIP) [file pone.0309727.s002.zip › Appendix I S13.tif]

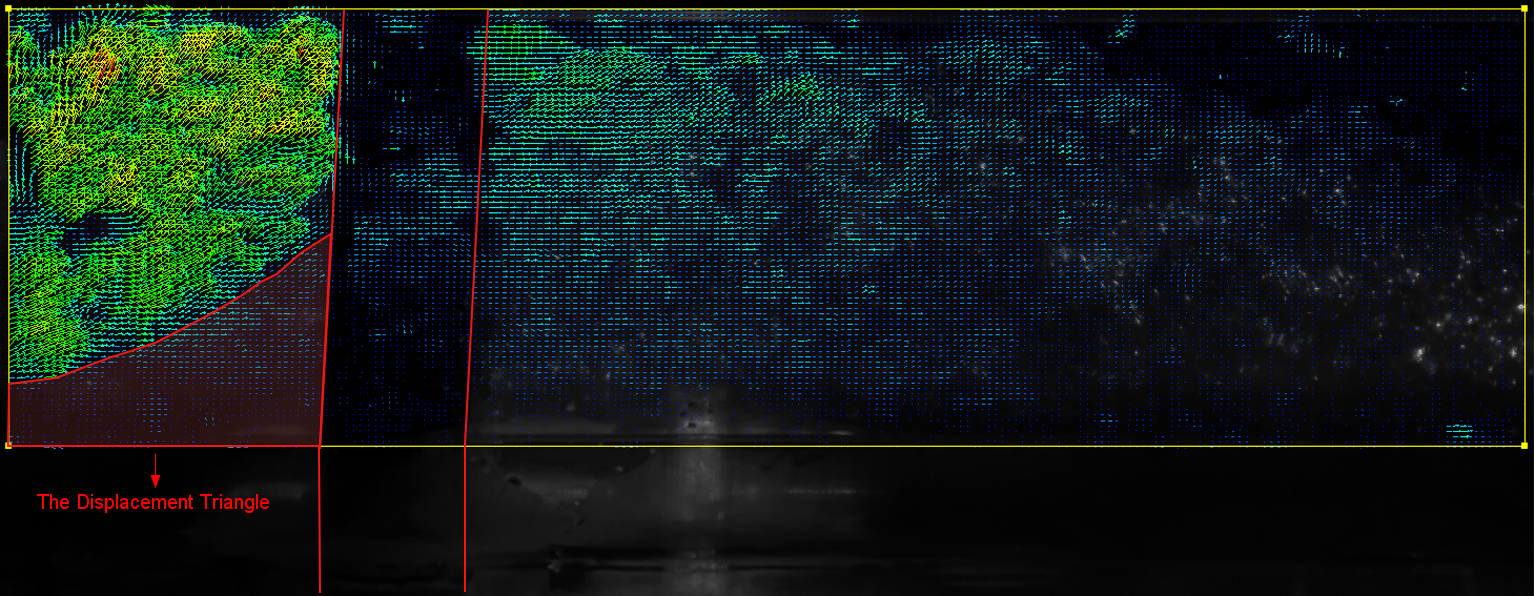

Supplement: S1 Appendix — Appendix Ⅰ S1 1st test image. Appendix Ⅰ S2 2nd test image. Appendix Ⅰ S3 3rd test image. Appendix Ⅰ S4 4th test image. Appendix Ⅰ S5 5th test image. Appendix Ⅰ S6 6th test image. Appendix Ⅰ S7 7th test image. Appendix Ⅰ S8 8th test image. Appendix Ⅰ S9 9th test image. Appendix Ⅰ S10 10th test image. Appendix Ⅰ S11 11th test image. Appendix Ⅰ S12 12th test image. Appendix Ⅰ S13 13th test image. Appendix Ⅰ S14 14th test image. Appendix Ⅰ S15 15th test image. Appendix Ⅰ S16 16th test image. Appendix Ⅰ S17 17th test image. Appendix Ⅰ S18 18th test image. Appendix Ⅰ S19 19th test image. Appendix Ⅰ S20 20th test image. (ZIP) [file pone.0309727.s002.zip › Appendix I S14.tif]

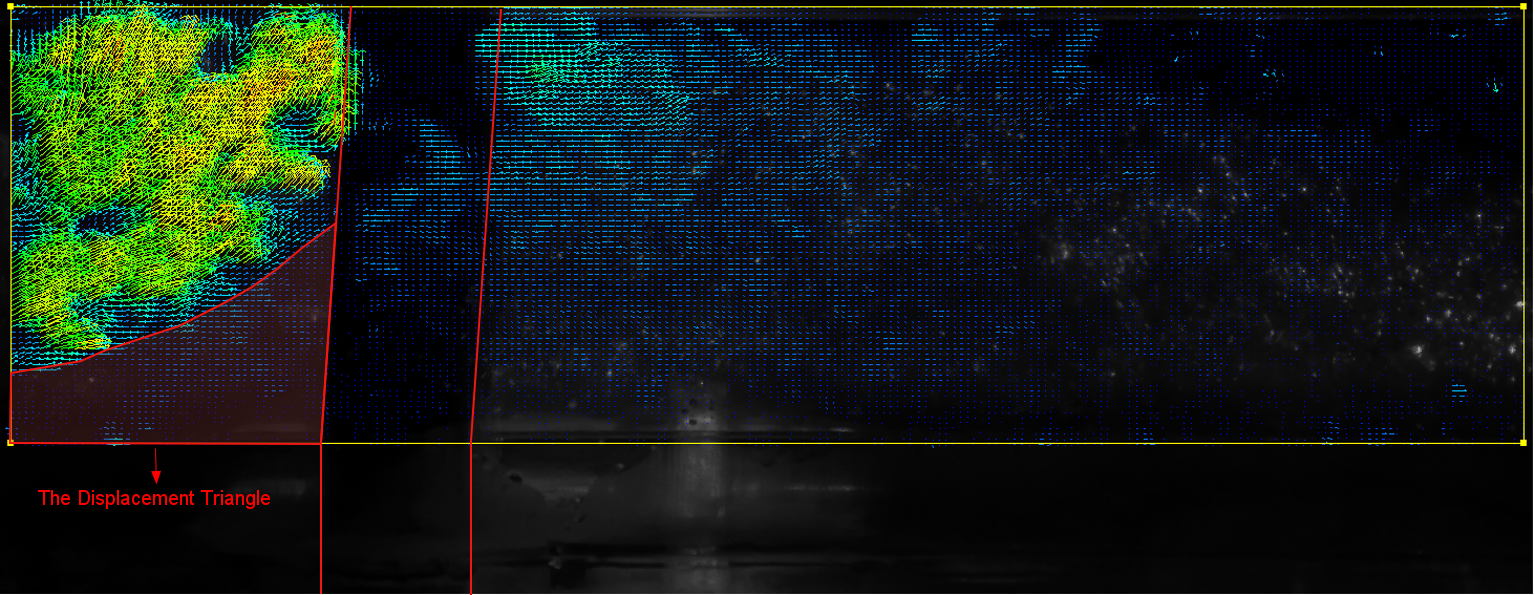

Supplement: S1 Appendix — Appendix Ⅰ S1 1st test image. Appendix Ⅰ S2 2nd test image. Appendix Ⅰ S3 3rd test image. Appendix Ⅰ S4 4th test image. Appendix Ⅰ S5 5th test image. Appendix Ⅰ S6 6th test image. Appendix Ⅰ S7 7th test image. Appendix Ⅰ S8 8th test image. Appendix Ⅰ S9 9th test image. Appendix Ⅰ S10 10th test image. Appendix Ⅰ S11 11th test image. Appendix Ⅰ S12 12th test image. Appendix Ⅰ S13 13th test image. Appendix Ⅰ S14 14th test image. Appendix Ⅰ S15 15th test image. Appendix Ⅰ S16 16th test image. Appendix Ⅰ S17 17th test image. Appendix Ⅰ S18 18th test image. Appendix Ⅰ S19 19th test image. Appendix Ⅰ S20 20th test image. (ZIP) [file pone.0309727.s002.zip › Appendix I S15.tif]

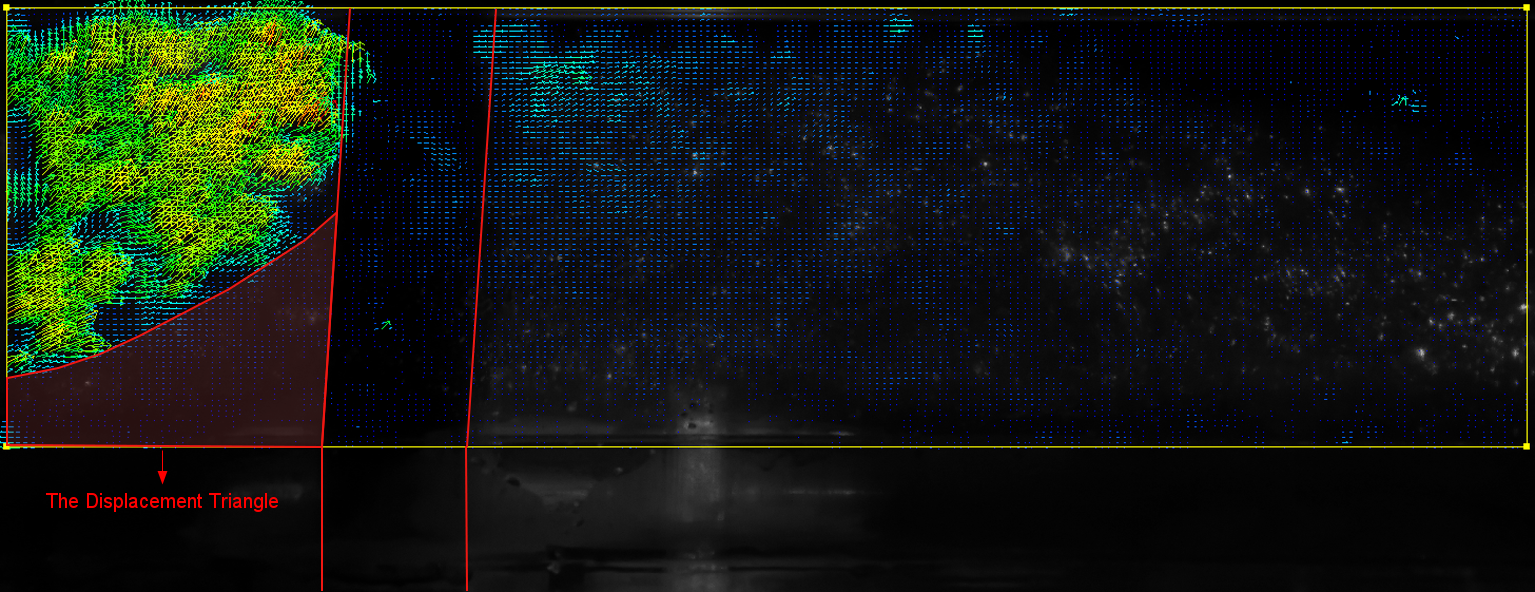

Supplement: S1 Appendix — Appendix Ⅰ S1 1st test image. Appendix Ⅰ S2 2nd test image. Appendix Ⅰ S3 3rd test image. Appendix Ⅰ S4 4th test image. Appendix Ⅰ S5 5th test image. Appendix Ⅰ S6 6th test image. Appendix Ⅰ S7 7th test image. Appendix Ⅰ S8 8th test image. Appendix Ⅰ S9 9th test image. Appendix Ⅰ S10 10th test image. Appendix Ⅰ S11 11th test image. Appendix Ⅰ S12 12th test image. Appendix Ⅰ S13 13th test image. Appendix Ⅰ S14 14th test image. Appendix Ⅰ S15 15th test image. Appendix Ⅰ S16 16th test image. Appendix Ⅰ S17 17th test image. Appendix Ⅰ S18 18th test image. Appendix Ⅰ S19 19th test image. Appendix Ⅰ S20 20th test image. (ZIP) [file pone.0309727.s002.zip › Appendix I S16.tif]

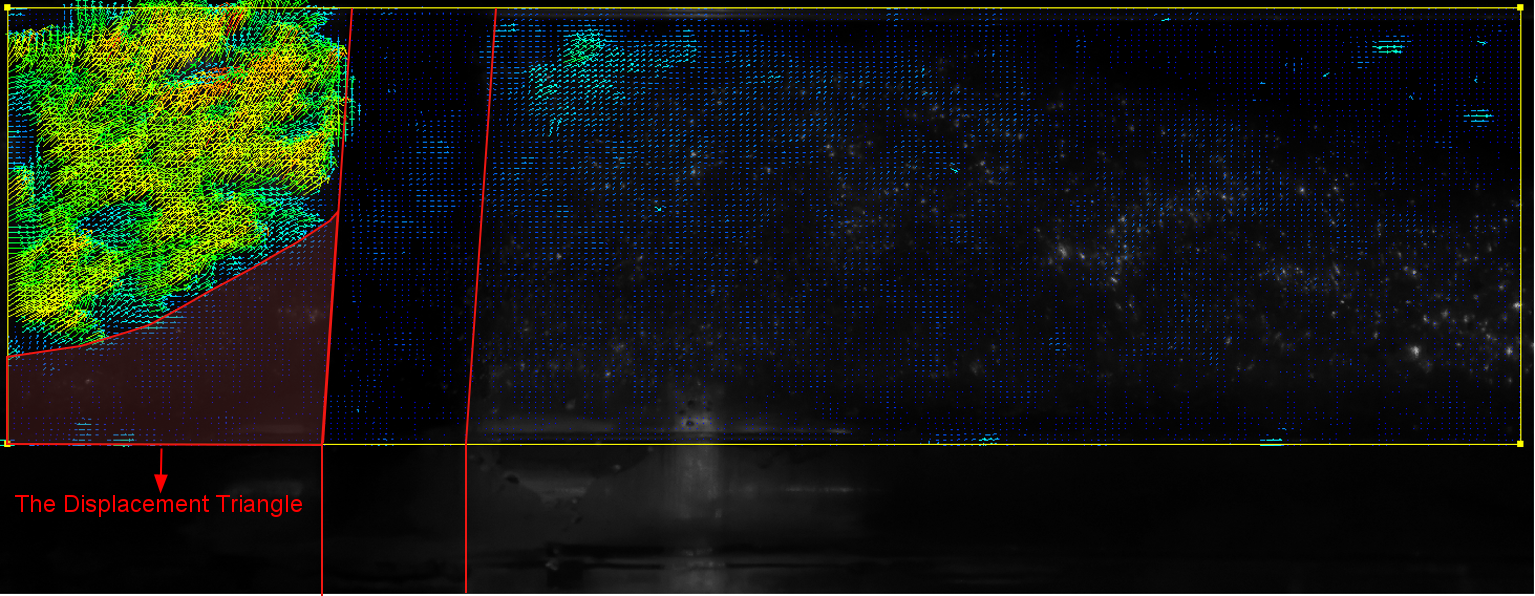

Supplement: S1 Appendix — Appendix Ⅰ S1 1st test image. Appendix Ⅰ S2 2nd test image. Appendix Ⅰ S3 3rd test image. Appendix Ⅰ S4 4th test image. Appendix Ⅰ S5 5th test image. Appendix Ⅰ S6 6th test image. Appendix Ⅰ S7 7th test image. Appendix Ⅰ S8 8th test image. Appendix Ⅰ S9 9th test image. Appendix Ⅰ S10 10th test image. Appendix Ⅰ S11 11th test image. Appendix Ⅰ S12 12th test image. Appendix Ⅰ S13 13th test image. Appendix Ⅰ S14 14th test image. Appendix Ⅰ S15 15th test image. Appendix Ⅰ S16 16th test image. Appendix Ⅰ S17 17th test image. Appendix Ⅰ S18 18th test image. Appendix Ⅰ S19 19th test image. Appendix Ⅰ S20 20th test image. (ZIP) [file pone.0309727.s002.zip › Appendix I S17.tif]

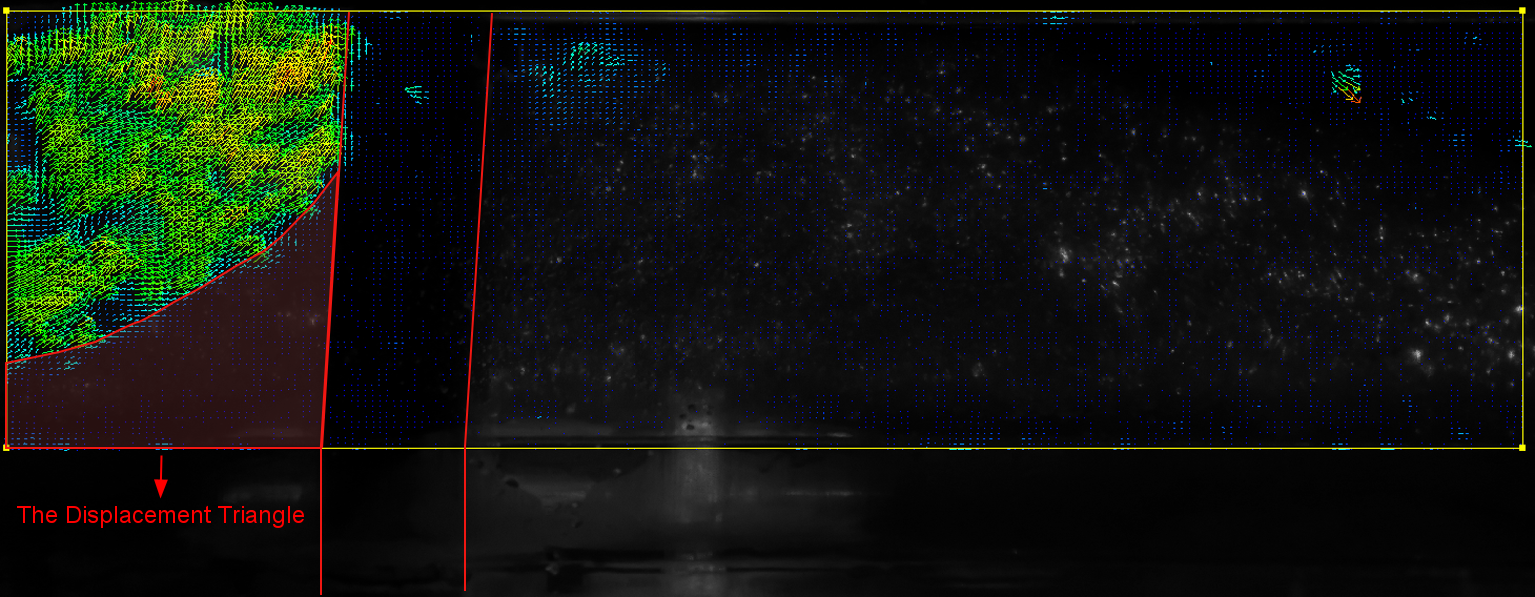

Supplement: S1 Appendix — Appendix Ⅰ S1 1st test image. Appendix Ⅰ S2 2nd test image. Appendix Ⅰ S3 3rd test image. Appendix Ⅰ S4 4th test image. Appendix Ⅰ S5 5th test image. Appendix Ⅰ S6 6th test image. Appendix Ⅰ S7 7th test image. Appendix Ⅰ S8 8th test image. Appendix Ⅰ S9 9th test image. Appendix Ⅰ S10 10th test image. Appendix Ⅰ S11 11th test image. Appendix Ⅰ S12 12th test image. Appendix Ⅰ S13 13th test image. Appendix Ⅰ S14 14th test image. Appendix Ⅰ S15 15th test image. Appendix Ⅰ S16 16th test image. Appendix Ⅰ S17 17th test image. Appendix Ⅰ S18 18th test image. Appendix Ⅰ S19 19th test image. Appendix Ⅰ S20 20th test image. (ZIP) [file pone.0309727.s002.zip › Appendix I S18.tif]

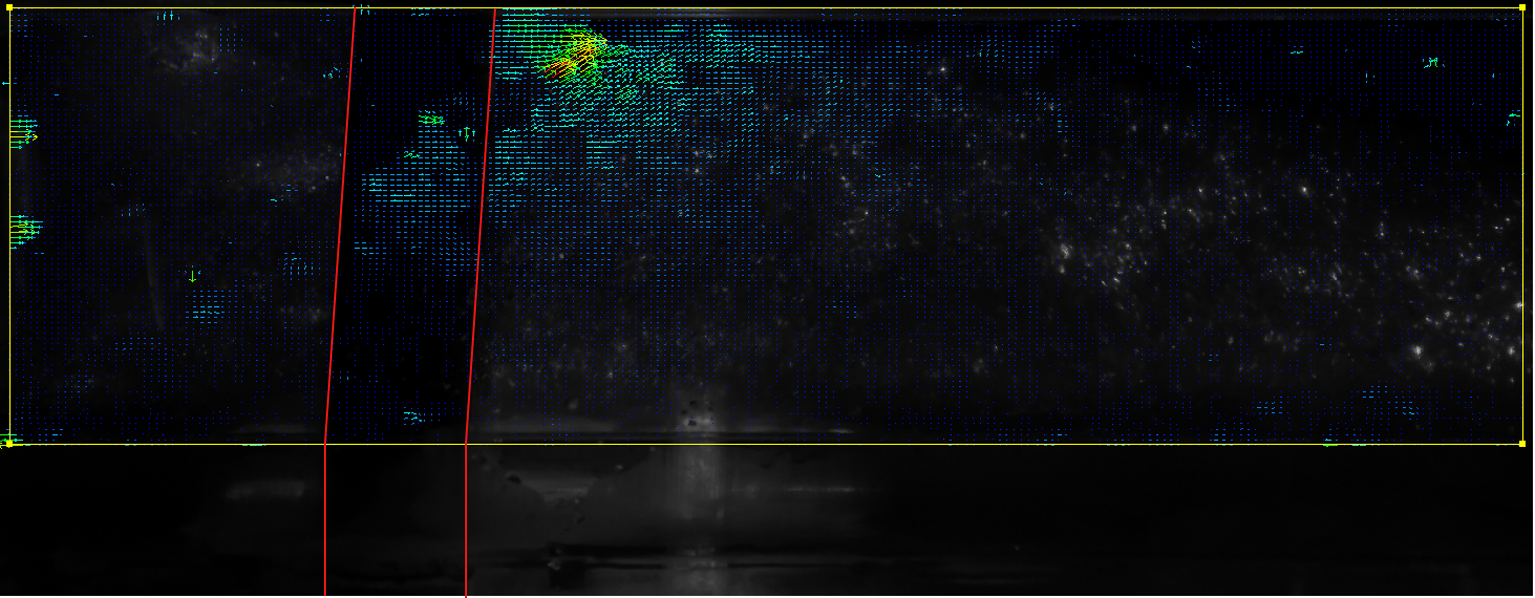

Supplement: S1 Appendix — Appendix Ⅰ S1 1st test image. Appendix Ⅰ S2 2nd test image. Appendix Ⅰ S3 3rd test image. Appendix Ⅰ S4 4th test image. Appendix Ⅰ S5 5th test image. Appendix Ⅰ S6 6th test image. Appendix Ⅰ S7 7th test image. Appendix Ⅰ S8 8th test image. Appendix Ⅰ S9 9th test image. Appendix Ⅰ S10 10th test image. Appendix Ⅰ S11 11th test image. Appendix Ⅰ S12 12th test image. Appendix Ⅰ S13 13th test image. Appendix Ⅰ S14 14th test image. Appendix Ⅰ S15 15th test image. Appendix Ⅰ S16 16th test image. Appendix Ⅰ S17 17th test image. Appendix Ⅰ S18 18th test image. Appendix Ⅰ S19 19th test image. Appendix Ⅰ S20 20th test image. (ZIP) [file pone.0309727.s002.zip › Appendix I S19.tif]

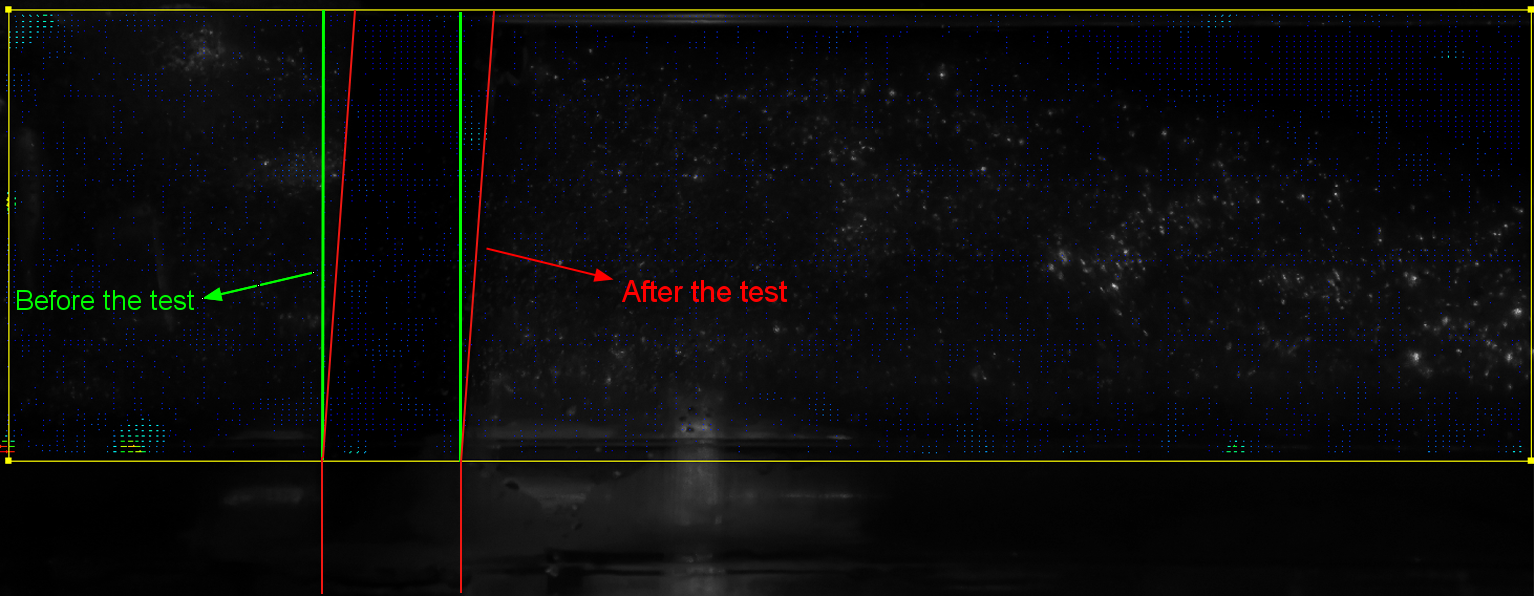

Supplement: S1 Appendix — Appendix Ⅰ S1 1st test image. Appendix Ⅰ S2 2nd test image. Appendix Ⅰ S3 3rd test image. Appendix Ⅰ S4 4th test image. Appendix Ⅰ S5 5th test image. Appendix Ⅰ S6 6th test image. Appendix Ⅰ S7 7th test image. Appendix Ⅰ S8 8th test image. Appendix Ⅰ S9 9th test image. Appendix Ⅰ S10 10th test image. Appendix Ⅰ S11 11th test image. Appendix Ⅰ S12 12th test image. Appendix Ⅰ S13 13th test image. Appendix Ⅰ S14 14th test image. Appendix Ⅰ S15 15th test image. Appendix Ⅰ S16 16th test image. Appendix Ⅰ S17 17th test image. Appendix Ⅰ S18 18th test image. Appendix Ⅰ S19 19th test image. Appendix Ⅰ S20 20th test image. (ZIP) [file pone.0309727.s002.zip › Appendix I S20.tif]

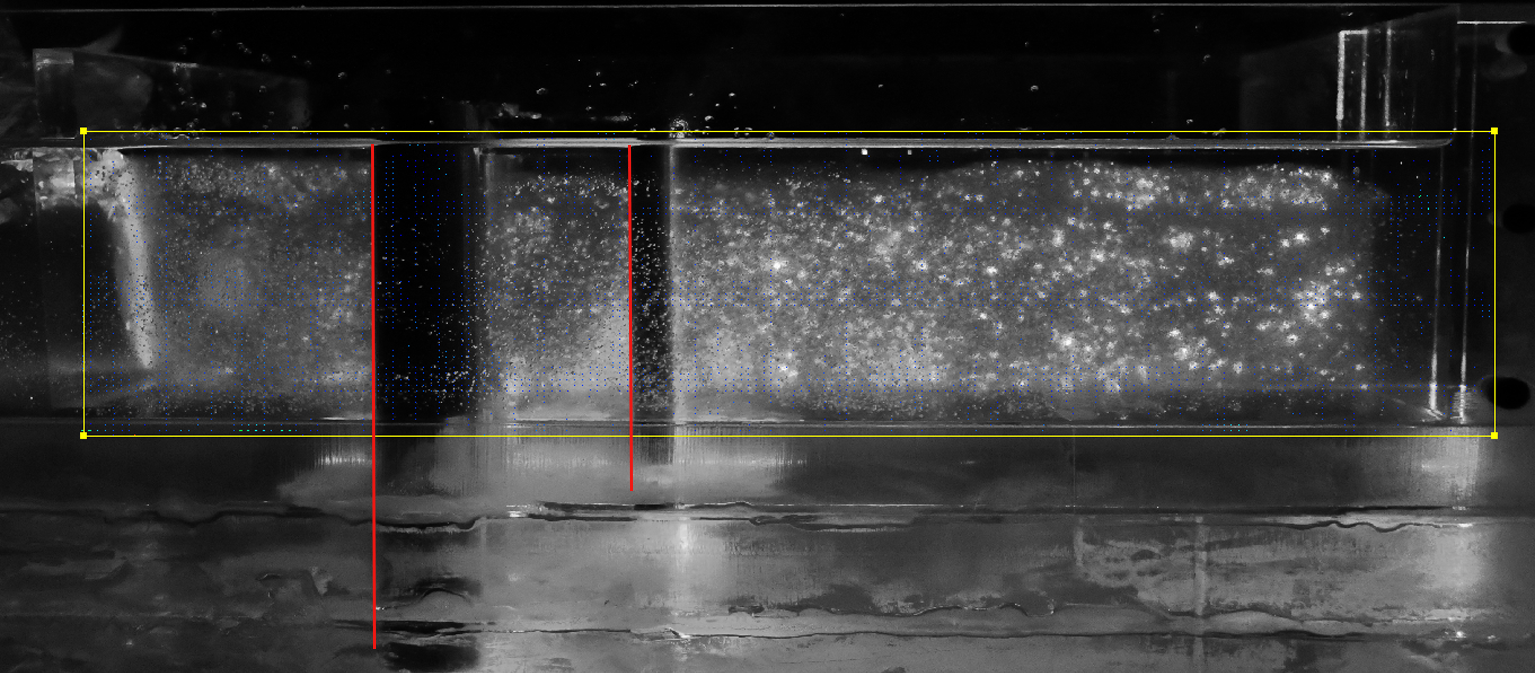

Supplement: S2 Appendix — Appendix Ⅱ S1 1st test image. Appendix Ⅱ S2 2nd test image. Appendix Ⅱ S3 3rd test image. Appendix Ⅱ S4 4th test image. Appendix Ⅱ S5 5th test image. (ZIP) [file pone.0309727.s003.zip › Appendix II S1.tif]

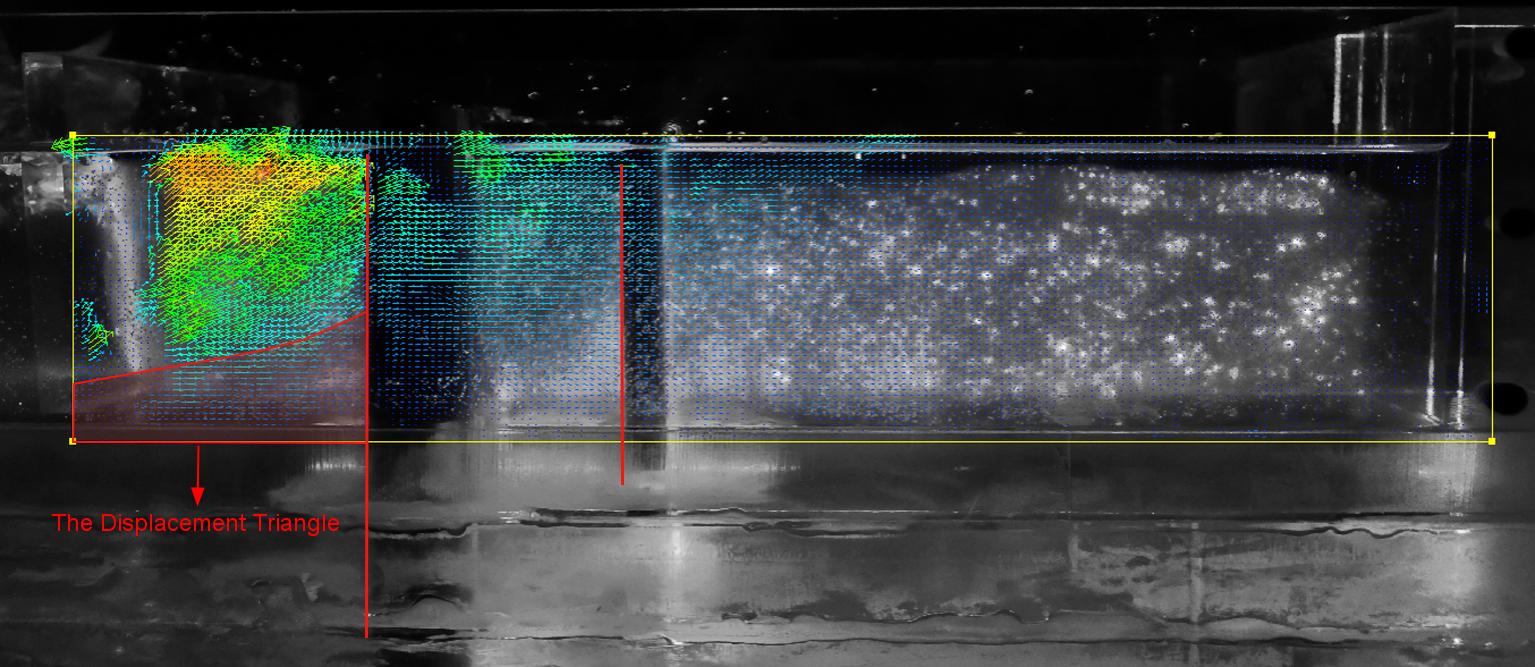

Supplement: S2 Appendix — Appendix Ⅱ S1 1st test image. Appendix Ⅱ S2 2nd test image. Appendix Ⅱ S3 3rd test image. Appendix Ⅱ S4 4th test image. Appendix Ⅱ S5 5th test image. (ZIP) [file pone.0309727.s003.zip › Appendix II S2.tif]

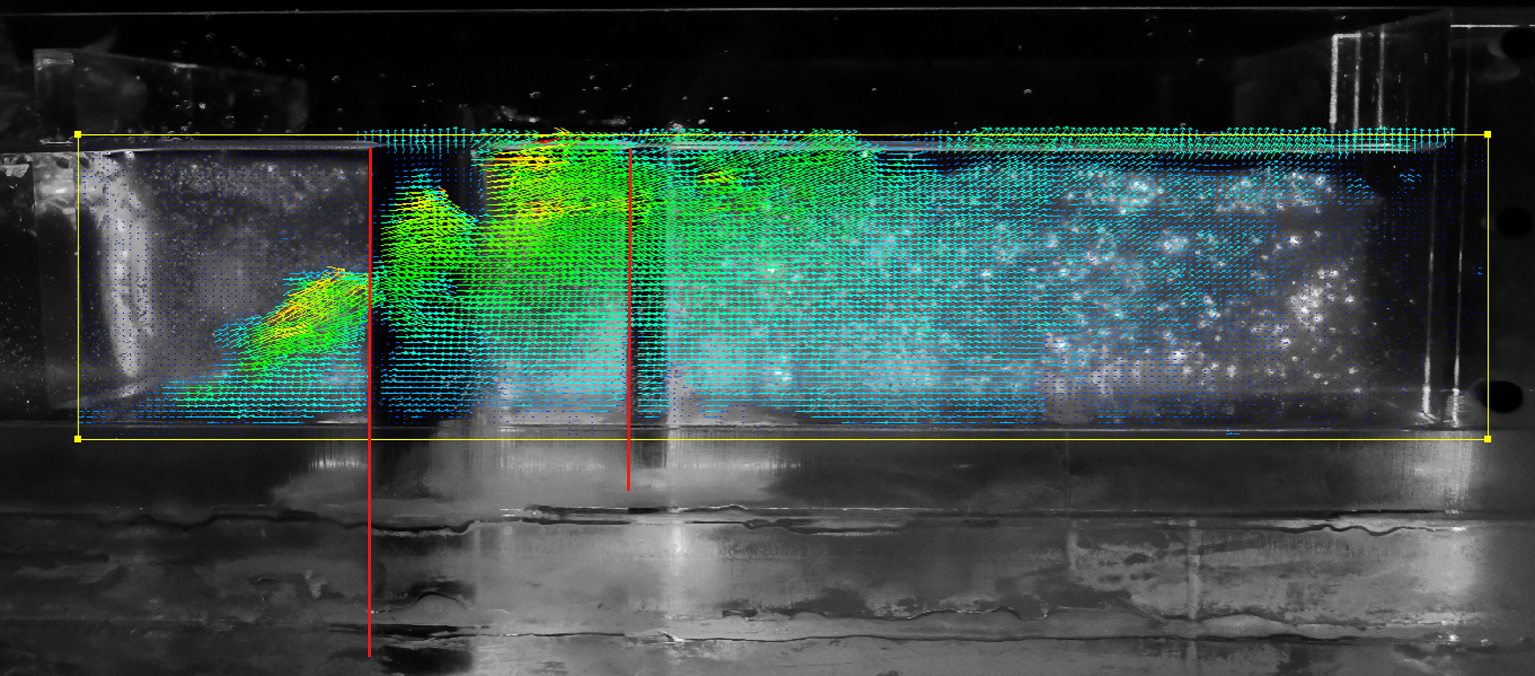

Supplement: S2 Appendix — Appendix Ⅱ S1 1st test image. Appendix Ⅱ S2 2nd test image. Appendix Ⅱ S3 3rd test image. Appendix Ⅱ S4 4th test image. Appendix Ⅱ S5 5th test image. (ZIP) [file pone.0309727.s003.zip › Appendix II S3.tif]

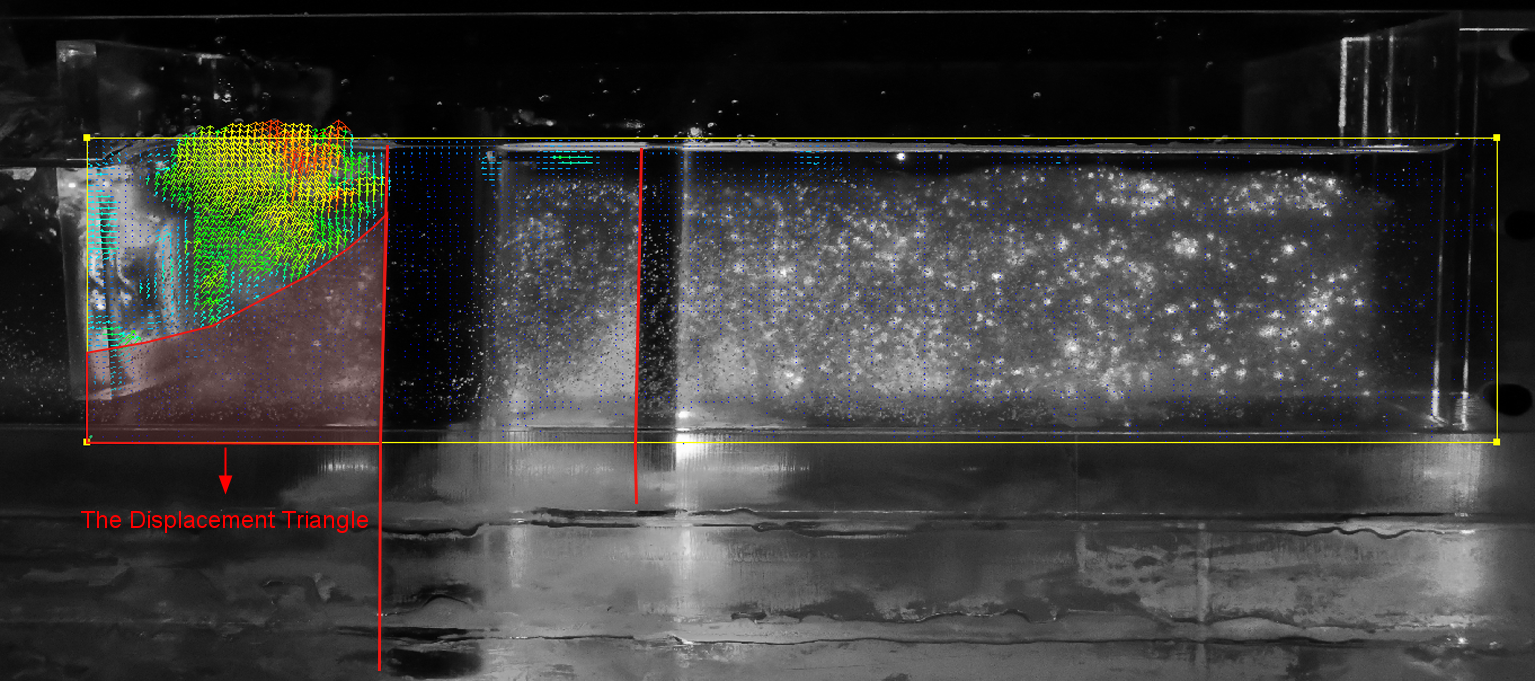

Supplement: S2 Appendix — Appendix Ⅱ S1 1st test image. Appendix Ⅱ S2 2nd test image. Appendix Ⅱ S3 3rd test image. Appendix Ⅱ S4 4th test image. Appendix Ⅱ S5 5th test image. (ZIP) [file pone.0309727.s003.zip › Appendix II S4.tif]

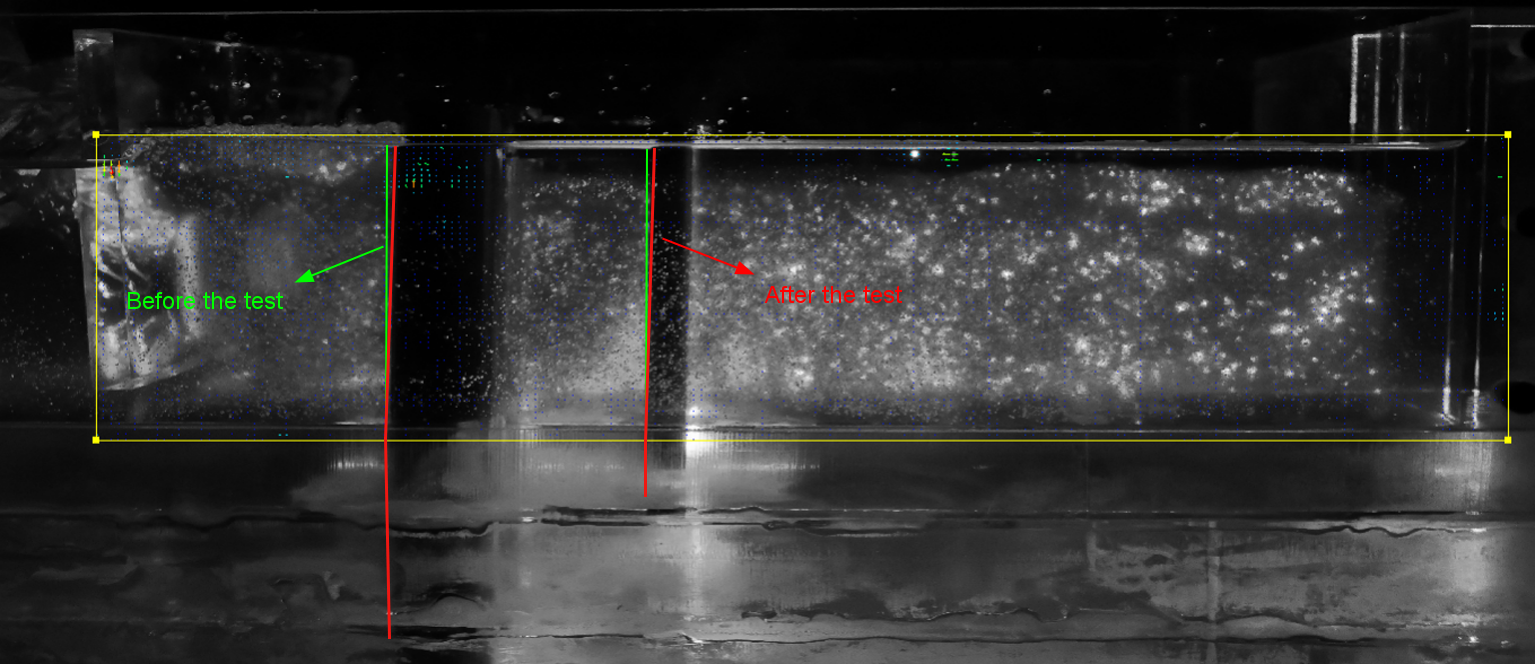

Supplement: S2 Appendix — Appendix Ⅱ S1 1st test image. Appendix Ⅱ S2 2nd test image. Appendix Ⅱ S3 3rd test image. Appendix Ⅱ S4 4th test image. Appendix Ⅱ S5 5th test image. (ZIP) [file pone.0309727.s003.zip › Appendix II S5.tif]
